# Pulse Oximetry Versus Arterial Co-Oximetry During Exercise: A Systematic Review of Agreement and Sensor Technology Reporting Gaps

**DOI:** 10.3390/bios16100565

**Published:** 2026-10-09

**Authors:** Shu Xie, Xueming Wen

**Affiliations:** School of Exercise and Health, Shanghai University of Sport, 650 Qingyuanhuan Road, Yangpu District, Shanghai 200438, China; xieshu@sus.edu.cn

**Keywords:** arterial co-oximetry, biosensor validation, exercise testing, motion artifact, optical biosensors, pulse oximetry, systematic review

## Abstract

Pulse oximetry is an optical biosensor for peripheral oxygen-saturation monitoring, but its agreement with arterial co-oximetry during exercise remains uncertain. We searched three databases through 30 August 2026 and conducted supplementary Embase searching in September. Sixteen studies yielded 46 device- or condition-level outcomes. Across four peak/maximal cohorts, mean bias ranged from −0.88 to +2.63 percentage points. Three reported paired SDs ranged from 2.75 to 4.60 points; a fourth was conditionally reconstructed. Primary synthesis was structured and descriptive. Post hoc random-effects mean-bias analyses of three reported-SD cohorts and a conditional fourth were imprecise and clinically heterogeneous, and did not characterize individual agreement. A descriptive technology audit found complete device identification in 14 studies and sensor-site reporting in 15, but no study fully reported numerical emitter wavelengths. Averaging/update behavior was fully reported in two studies, algorithm/post-processing detail in three, motion handling in six, and failure/missingness in seven. The author-agreed appraisal was interpreted descriptively without validated aggregate risk or certainty grades. These data do not establish a universal correction factor or a transportable substitution rule. No prospective registration, incomplete supplementary-search export reconciliation and reference-construct ambiguity limit interpretation. More complete sensor, reference-method and agreement reporting is needed.

## 1. Background

Pulse oximetry is an optical biosensor that estimates peripheral oxygen saturation (SpO2) from wavelength-dependent pulsatile light absorption. The reported value is produced by a measurement chain that includes emitters and photodetectors, tissue and sensor site, probe–tissue contact, local perfusion, motion, signal averaging, and proprietary calibration and filtering [1,2,3,4,5,6,7,8]. During exercise, each component may change while direct arterial co-oximetry remains intermittent and technically demanding. A useful continuous monitor is therefore not automatically interchangeable with an arterial reference.

Agreement requires more than correlation or a small group mean difference. The distribution of paired differences and its limits determine uncertainty for an individual measurement [9,10,11,12]. In the largest included peak-exercise cohort, mean SpO2 exceeded the source-reported arterial saturation by 2.63 percentage points, but the limits of agreement extended from −2.9 to +8.1 [13]. Four participants in another cohort had SpO2 above 88% while the paired source-reported SaO2 was 88% or lower [14]. The acceptability of such disagreement depends on the decision and its consequences; no context-independent margin can be assumed.

Historical reviews recognized exercise-related effects of motion, perfusion, hypoxemia, and device characteristics [15,16,17]. A 2024 review addressed skin tone but did not establish exercise-specific effects [18]. These reviews did not combine an exercise-window restriction, direct arterial co-oximetry, sign-harmonized paired outcomes, dependence-aware synthesis, and structured technology reporting. Current US Food and Drug Administration draft guidance emphasizes objectively measured skin pigmentation and reporting of motion, perfusion, sensor site, and device configuration [19]. It remains nonbinding and is not an exercise-validation standard.

We reviewed paired SpO2 and direct arterial co-oximetry measurements obtained during dynamic exercise or within 2 min after cessation in adults. The review addressed the direction and magnitude of agreement and whether the available evidence could support a generalizable substitution rule. The protocol was not prospectively registered. A separate, exploratory technology-reporting audit was added after the initial synthesis to describe the device information available for interpreting disagreement. This post hoc audit did not change eligibility or the agreement outcomes and cannot establish which technical features caused differences between studies.

## 2. Methods

### 2.1. Protocol and Eligibility

We followed the Preferred Reporting Items for Systematic Reviews and Meta-Analyses (PRISMA) 2020 statement [20]. Database searching preceded the operational protocol freeze on 29 August 2026. The review was not prospectively registered, and subsequent decisions were recorded as dated amendments. Eligible full reports enrolled adults aged 18 years or older. They had to provide a paired comparison between pulse-oximeter SpO2 and a direct arterial co-oximetry oxygen-saturation endpoint during dynamic exercise or within 2 min after cessation. The comparison could be interpreted quantitatively or qualitatively. Mixed-age reports required separable adult results. We excluded rest-only or static-hypoxia studies and reports using venous, capillary, calculated, or unconfirmed blood-gas saturation. Reports without recoverable numerical agreement statistics could enter qualitative synthesis but not quantitative preparation; reports without an interpretable paired exercise-window comparison were excluded. Post hoc corrected indices were retained only as secondary technology-history evidence. Instrument capability alone was insufficient to infer co-oximetry. This clarification was documented after the operational protocol freeze. A later source-based full-text reassessment found conflicting reference-method descriptions for one provisionally included report [21]. The authors confirmed its exclusion from the 16-study original-database evidence set because direct arterial co-oximetry of the saturation endpoint used for the SpO2 comparison could not be established. Direct arterial co-oximetry was required to anchor the comparison to an arterial measurement rather than saturation calculated from blood-gas variables. This criterion does not imply an error-free reference: analyzer configuration, the saturation construct, and temporal pairing still require scrutiny. The 2 min post-exercise boundary was an operational eligibility rule, not a claim that recovery measurements are physiologically equivalent to measurements during exercise. Exercise, recovery, and combined-period observations therefore require separate interpretation; the boundary itself was not validated by this review.

### 2.2. Literature Search

We searched PubMed, Web of Science Core Collection, and SPORTDiscus with Full Text without language, year, or document-type restrictions. All three original databases were updated through 30 August 2026. Search concepts covered pulse oximetry, exercise, and arterial sampling or co-oximetry. Complete strategies, dates, filters, yields, and export hashes appear in Supplementary File S1 (e-Appendix S1) and Supplementary File S2 (e-Table S1). A post hoc supplementary Embase.com search was performed on 29 September 2026. The narrower search displayed 1196 article records and 231 clinical-trial records; a broader mapped variant displayed 1255 and 263, respectively. These searches were not added together. The exact Embase syntax and export audit are in Supplementary File S1 (e-Appendix S1). The authors selected search #225 (1196 article records) as the final supplementary search; #207 is retained only as a historical export audit.

On August 31, we used OpenAlex as a discovery graph for backward and forward citation searching. Seeds comprised 17 provisionally included reports, one of which was later excluded after full-text reference-method reassessment, two historical exercise reviews, one broad adult meta-analysis, and a 2024 skin-tone review [15,16,17,18]. The 21 seeds yielded 1506 unique graph records. Of these, 164 matched the database corpus and 1342 new records underwent independent title/abstract screening by two reviewers; after consensus, no record was promoted to full-text retrieval. Detailed methods and dispositions appear in Supplementary File S1 (e-Appendix S2). Database overlap and recovery of known studies were descriptive coverage checks, not evidence that the search was exhaustive. OpenAlex citation discovery was supplementary to the three-database searches and could not compensate for databases not searched. Embase was searched later as a supplementary source, but the available exports do not support a complete merged identification and screening flow. Scopus and CINAHL were not searched.

### 2.3. Selection and Abstraction

Original exports were retained and normalized. Duplicate merging used DOI, PMID, and conservative exact-title rules; later variants were linked before study counting. Two reviewers independently screened the original three-database and citation-search titles and abstracts against the same eligibility criteria and independently assessed each retrieved full text. Disagreements were resolved through discussion and recorded as consensus decisions. AI-assisted triage organized records and proposed dispositions but did not replace either reviewer’s final judgment. Reviewer-level disagreement counts were not retained, so inter-rater agreement was not calculated. For the later Embase subset, both authors report a consensus title/abstract exclusion of a supplied 725-record queue and seven separate reports. Separate reviewer-level decision forms for this subset were not supplied; this provenance differs from the retained original-database screening record.

Potentially eligible or uncertain records proceeded to full-text review. Each consensus exclusion received one primary reason, and unavailable reports were classified as not retrieved rather than ineligible. Study and outcome forms captured participants, exercise protocol, device and site, arterial reference, timing, signal failures, paired participants and observations, and agreement results.

Bias was sign-harmonized as SpO2 minus the source-reported arterial co-oximetry oxygen-saturation endpoint; positive values denote pulse-oximeter overestimation. Source-reported SaO2 and fractional HbO2 were recorded separately. A source label of SaO2 was not taken as proof of a particular functional or fractional calculation. Source direction, endpoint construct, SD, confidence interval, and genuine limits of agreement were retained separately. For a two-sided, unadjusted Student t confidence interval [L, U] for a paired-mean difference based on *n* independent participant-level differences, the conditional reconstruction is SDdifference = (U − L) × sqrt(*n*)/(2 × t0.975, *n* − 1). This uses the interval for the paired differences, not intervals for the two separate measurement means; an additional between-method correlation is not required under these assumptions. The expression is not directly applicable to an adjusted, bootstrap, or repeated-measures model interval without its variance structure. Because the exact interval construction in the 2024 peak cohort was not established, its reconstructed SD was treated as conditional and excluded from the reported-only SD summary. A descriptive sensitivity comparison retained all four reported mean differences while comparing dispersion summaries with and without this reconstruction. Multiple devices, conditions, and repeated time points remained grouped within studies; this organization did not constitute a fitted statistical adjustment for dependence. The 46 outcome rows were not treated as 46 independent studies. Graphical or numerically nonrecoverable results remained qualitative. Data extraction and risk-of-bias assessment were performed by both reviewers, with final forms reflecting agreement after discussion. AI assistance supported record parsing, reconciliation, consistency checks, drafting, and reproducible table and visualization coding, but was not treated as an author or human reviewer.

Two otherwise eligible mixed-age reports were excluded because adult results were inseparable [22,23]. A post hoc qualitative sensitivity analysis tested whether their exercise comparisons could alter the pooling decision or central conclusion. Neither report entered the primary count or quantitative synthesis.

### 2.4. Technology-Focused Secondary Extraction

Following the Academic Editor’s pre-check, we performed an editor-requested post hoc technology-reporting audit of 17 provisionally included reports; final tabulations were restricted to 16 after reference-method reassessment. We extracted device manufacturer, model, and generation; optical geometry; sensor site; wavelength information; signal averaging or update behavior; motion-artifact handling; perfusion or signal-quality rules; probe fixation and ambient-light control; algorithm, calibration, or post-processing detail; and signal failure or missingness. Only information stated in the included report was coded. We did not infer unreported wavelengths, geometry, averaging, or proprietary algorithms from a device name or external manual.

Each domain was coded as reported when the article supplied device-specific operational detail, partial when it mentioned the domain without sufficient device-specific detail, or NR when it did not report the domain. Numerical emitter wavelengths were required for full wavelength reporting. Each code retained a source-text locator. The audit was descriptive, did not change eligibility or primary agreement outcomes, and was not used for causal attribution or device ranking.

### 2.5. Methodological Appraisal and Synthesis

Both authors checked and agreed on the final 192 item responses for the 16 included studies. Separate independent rating forms were not retained. The historical 12-item framework drew on QUADAS-2 domains, but QUADAS-2 concerns diagnostic accuracy rather than continuous agreement [24]. We therefore use the retained evidence as a descriptive methodological appraisal, distinguishing potential bias mechanisms, reporting or construct ambiguity, and eligibility checks. We do not calculate an overall risk-of-bias grade or use an any-no algorithm in the current synthesis. Missing reporting indicates uncertainty rather than proof of bias. Supplementary Files S2 and S3 provide the unchanged author-agreed responses, source locations and an interpretive codebook; original aggregate categories are retained only in the provenance archive. This descriptive framework is not an externally validated continuous-agreement appraisal tool.

The primary synthesis describes paired-mean differences in percentage points, paired SDs and individual limits of agreement where reported. The original review-specific compatibility rule required three independent studies within a common clinical and measurement stratum; this is not a universal statistical requirement. SWiM-guided direction-and-magnitude synthesis avoids significance vote counting [25]. We added a post hoc exploratory random-effects mean-bias analysis of three independent peak/maximal cohorts with reported paired SDs, and a four-cohort sensitivity analysis adding the conditional 2024 SD. One estimate per cohort was used; correlated devices, stages and overlapping subgroups were not treated as independent studies. Sampling variance was SD squared divided by the participant count, with REML between-study variance and modified Hartung–Knapp confidence intervals using k−1 degrees of freedom. Disease, device/site and arterial-construct differences limit the interpretation of the average. Pooling mean bias does not estimate individual agreement or a transferable correction factor. With only three or four heterogeneous cohorts, meta-regression and small-study tests were not performed. Supplementary File S8 reports inputs, assumptions and reproducible calculations.

We describe study limitations, inconsistency, indirectness and imprecision for each synthesis context. The historical GRADE-informed matrix [26] inherited unvalidated aggregate appraisal categories and is not used as a formal certainty assessment. Supplementary File S4 presents a narrative interpretation of these limitations without overall certainty grades.

### 2.6. Use of AI-Assisted Tools

During preparation of this review, the authors used the OpenAI Codex desktop (gpt-5.6-sol) agent to assist with bibliographic record organization, consistency checks, drafting, formatting, and reproducible table and data-visualization coding. The authors reviewed and edited all outputs, verified source-based claims and data, made all eligibility, appraisal, synthesis, and interpretation decisions, and take full responsibility for the content of the publication.

## 3. Results

**Figure 1:**
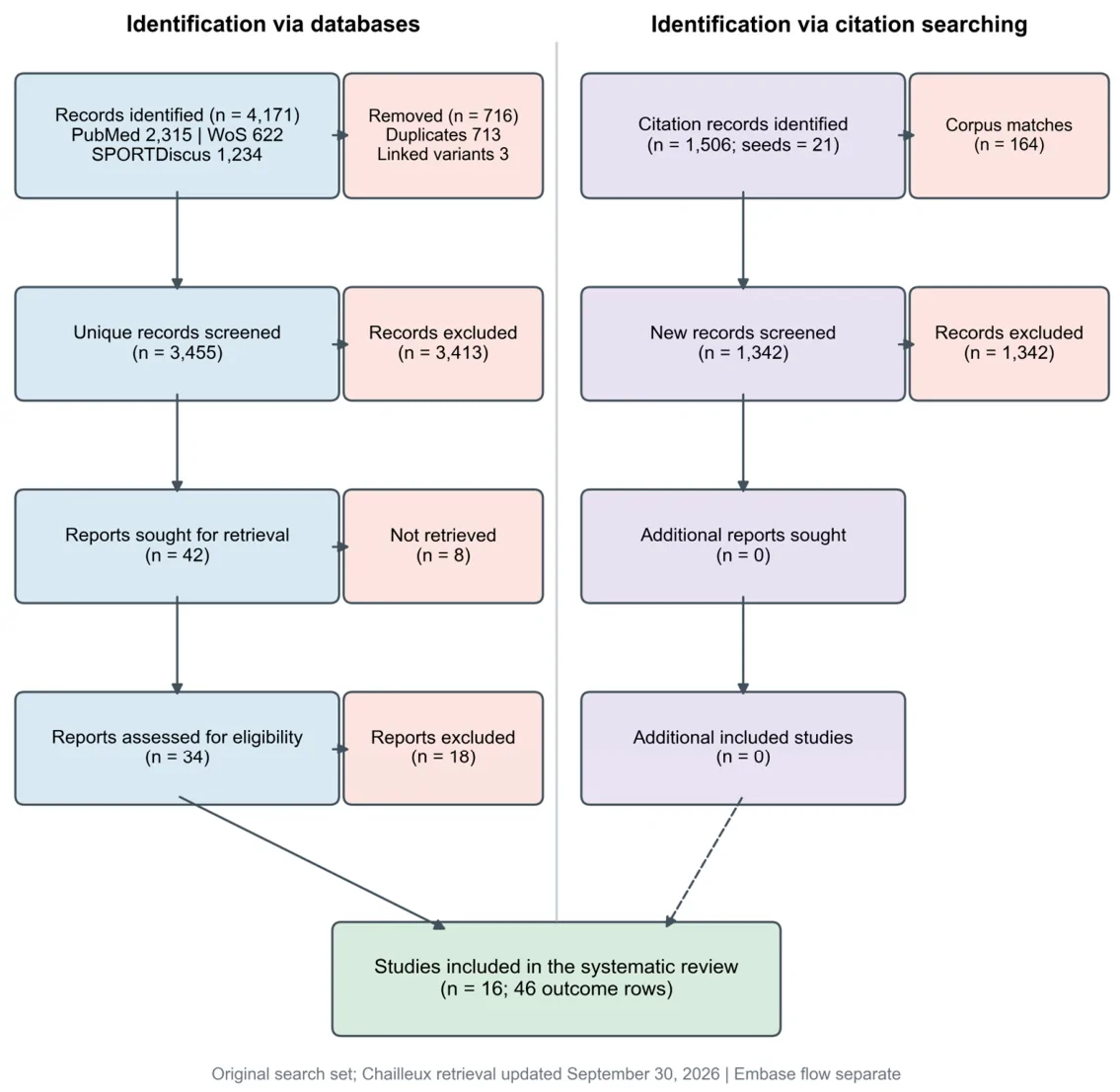
Study identification and selection. Original PubMed, Web of Science, and SPORTDiscus identification and citation discovery are shown. The supplementary Embase search is reported separately in Supplementary Files S1 and S8, including six records unavailable because of export failure. Of 1506 citation records, 164 were already represented in the database corpus. Two reviewers independently screened the original records and assessed retrieved full texts; citation screening yielded no additional reports. Eight reports were not retrieved and remain of unknown eligibility. Source data are in Supplementary File S6.

The original three-database searches identified 4171 records. Automated deduplication removed 713, and three later-linked variants were removed before study counting, leaving 3455 records screened. We excluded 3413 at title/abstract review and sought 42 reports. Eight reports were not retrieved, 34 were assessed, 18 were excluded, and 16 studies were included (Figure 1; Supplementary File S2: e-Table S2). Mollard et al. [21] was reclassified as a full-text exclusion after the authors confirmed that its incompatible reference-method descriptions do not establish direct arterial co-oximetry of the saturation endpoint used for the SpO2 comparison. On 30 September 2026, both authors confirmed that the previously unretrieved Chailleux report (PMID 2740587) had been obtained and excluded after full-text assessment under E04: the index did not meet the pulse-oximetry SpO2 criterion. This author-confirmed update changes retrieval and exclusion counts, not the included study set.

The final supplementary Embase search (#225, 29 September 2026) displayed 1196 article records. The supplied article export contained 1190 recoverable unique identifiers. The authors confirmed that the six-record shortfall resulted from an export failure. These six records were not available for screening and were not counted as duplicates, title/abstract exclusions or full-text exclusions. The authors confirmed completion of screening of the supplied records. Search #207 is retained only as audit history and is not added to the final search denominator. The 231 clinical-trial registry records are reported separately from article records. The author-confirmed exclusion queue contained 725 records. After nine links to the original corpus and five within-queue duplicates were removed, 711 distinct records remained; seven separately supplied, report-distinct records were also excluded at title/abstract screening. This 718-record audited subset is not a full-text exclusion count or the denominator of the entire Embase search. Figure 1 retains the original database and citation-search branches; supplementary Embase accounting is reported separately in Supplementary Files S1 and S8. No additional eligible study was identified among the supplied records.

Citation searching identified 1506 unique graph records. Of these, 164 were already represented in the database corpus, and 1342 were screened as a separate other-methods branch. The final two-reviewer consensus disposition identified no additional report for retrieval. Citation searching recovered all included studies and two reports already classified as not retrieved.

The 16 studies were published from 1985 through 2024 and enrolled eight to 751 participants. Nine contributed core numerical evidence attributable to the exercise window, four supplied indirect summaries that mixed exercise with other periods or conditions, and three supplied qualitative or secondary-index evidence. Populations included healthy volunteers, military recruits, endurance athletes, and adults evaluated for COPD, interstitial lung disease, beryllium sensitization or disease, or mixed indications [13,14,27,28,29,30,31]. Studies used cycle ergometry, treadmill walking, or invasive cardiopulmonary exercise testing. Finger, earlobe, and forehead sensors were represented. Device generations, sites, signal-handling rules, oxygenation conditions, and functional, fractional, or incompletely specified arterial endpoints varied. Several reports combined repeated observations or multiple configurations from the same cohort (Table 1; Supplementary File S2: e-Table S3).

### Sensor Technology and Reporting Completeness

**Figure 2:**
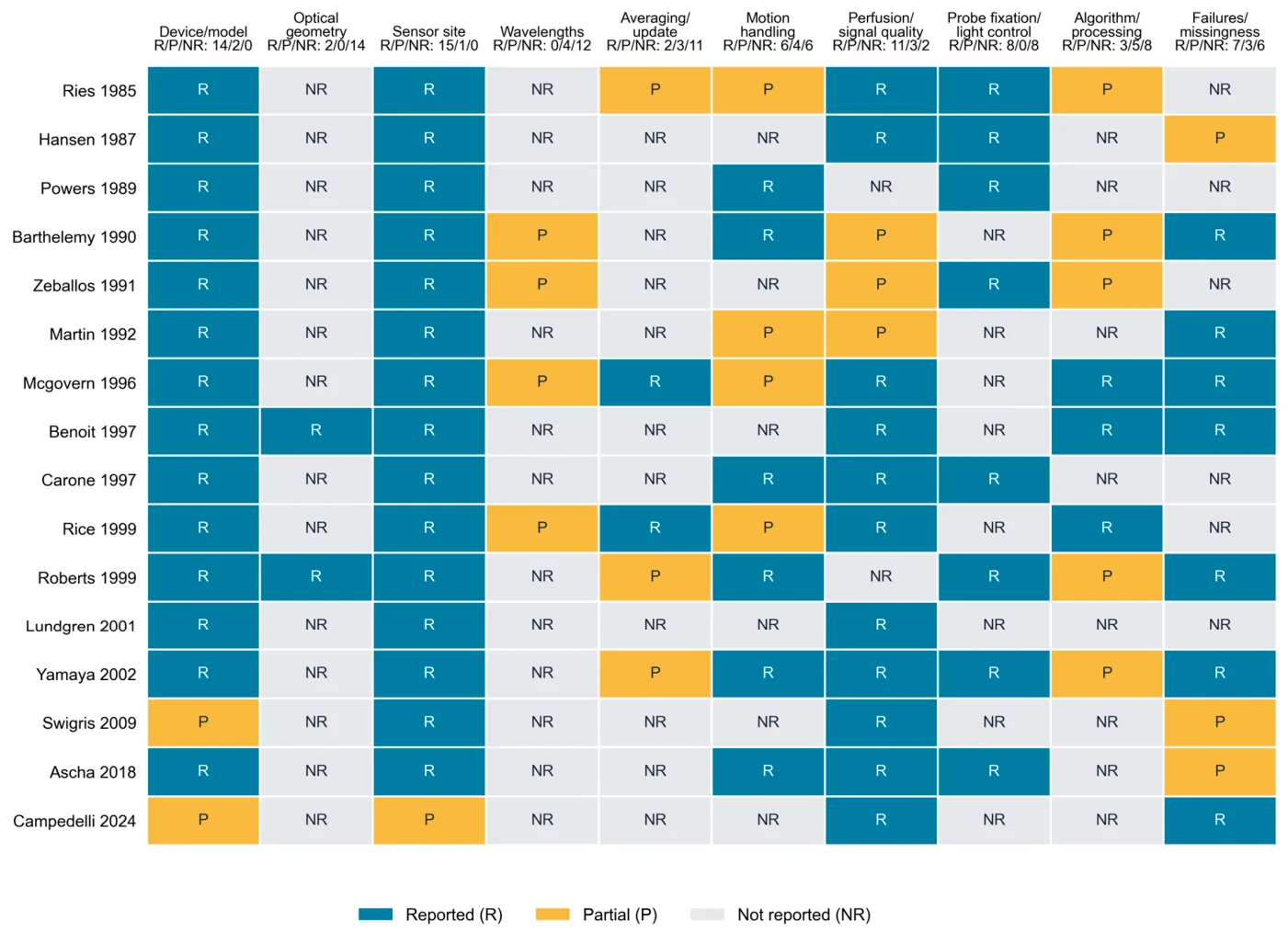
Study-level reporting of pulse-oximeter technology domains. The reporting audit is based on the 16 included studies [13,14,27,28,29,30,31,32,33,34,35,36,37,38,39,40]. Rows are ordered by publication year. R indicates device-specific operational detail; P indicates that a domain was mentioned but lacked sufficient device-specific detail; NR indicates not reported. Numerical wavelengths were required for R in the wavelength column. Model names were not used to infer geometry, wavelengths, averaging, or proprietary algorithms. Column headings give reported/partial/NR counts. Source-coded details and evidence locators are in Supplementary File S7.

Device identification was complete in 14 of 16 reports and partial in two; sensor site was complete in 15 and partial in one. Optical geometry was explicit in two reports. No report gave numerical emitter wavelengths; four partially described wavelength count or a general wavelength principle. Averaging or update behavior was complete in two reports and partial in three. Motion handling was complete in six and partial in four, whereas probe fixation or ambient-light control was complete in eight. Algorithm or post-processing detail was complete in three and partial in five. Signal failure or missingness was complete in seven and partial in three. Perfusion or signal-quality handling was complete in 11 and partial in three, but often consisted of a local intervention or quality rule rather than a perfusion-sensitivity experiment (Figure 2; Supplementary File S7).

Technology reporting was usually intervention-specific rather than architecture-complete. Examples included dual forehead probes with waveform and heart-rate concordance [13], a forehead-versus-digit comparison under identical Nellcor signal-detection algorithms [32], headband, splint, or ambient-light controls [28,32,33,35,37,40], perfusion enhancement or quality thresholds [27,31,33,34,35,38], and quantified signal loss [29,32,34,36,39,40]. No report covered every domain. Device, site, population, era, oxygenation, and handling strategies were usually confounded, so these observations did not support causal attribution or a device ranking.

**Figure 3:**
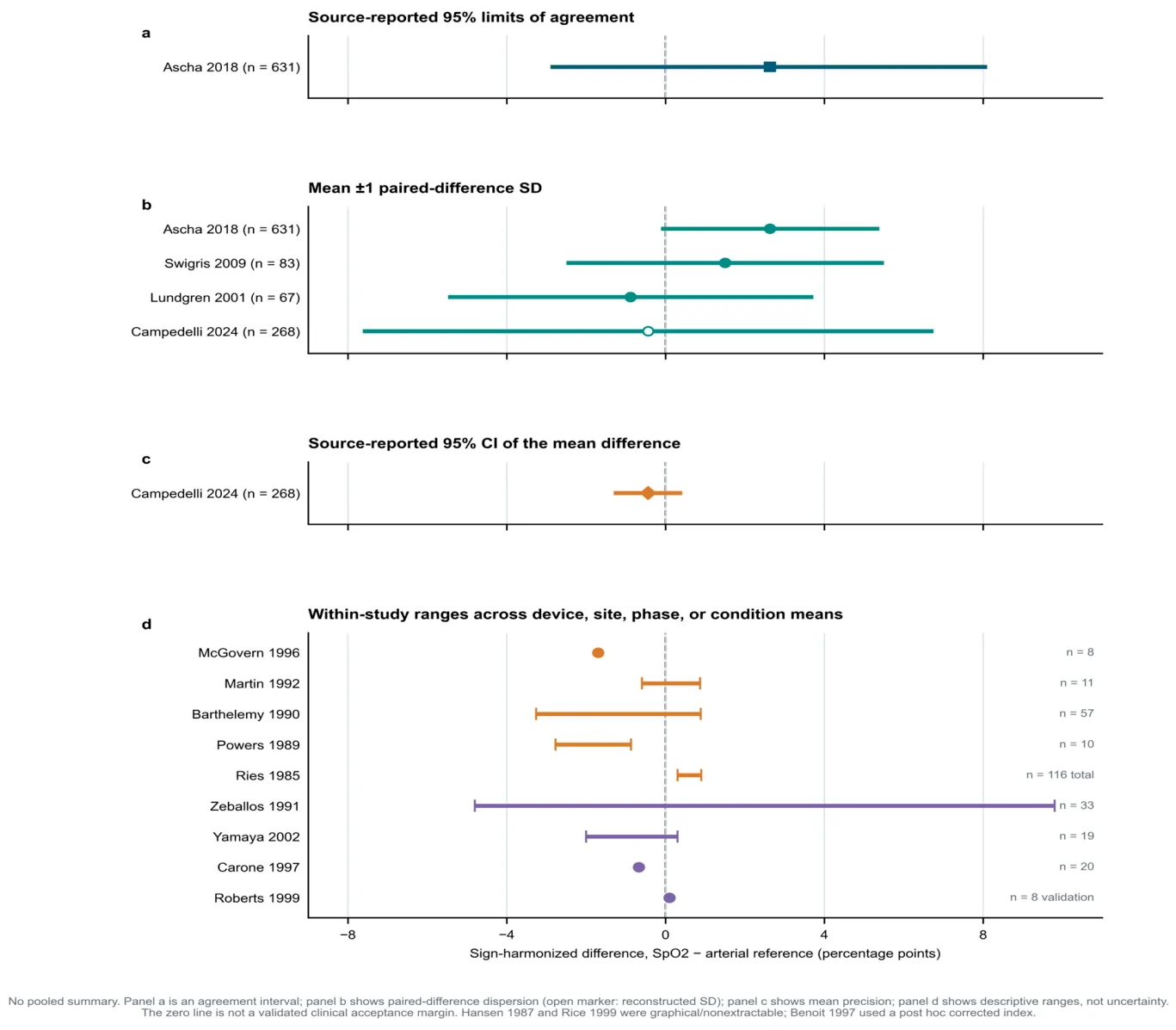
Non-pooled direction and magnitude of exercise agreement. Bias is sign-harmonized as SpO2 minus the source-reported arterial co-oximetry oxygen-saturation endpoint [13,14,27,28,29,30,31,32,33,34,35,36,37,38,39,40]. (**a**) The source-reported 95% limits of agreement for Ascha 2018. (**b**) Mean ± 1 paired-difference SD for three reported peak/maximal estimates, with the open Campedelli 2024 marker shown only as a conditional sensitivity comparison. Its SD assumes that the reported mean confidence interval is an ordinary unadjusted paired t interval with *n* = 268; it is excluded from the reported-only SD range. All four estimates use one peak/maximal pair per analyzed participant. (**c**) The source-reported 95% confidence interval for the Campedelli 2024 mean difference, shown separately because it represents precision of the mean rather than individual agreement. (**d**) Single reported means or within-study ranges across device, site, phase, or condition means for historical repeated comparisons (amber) and mixed-period or condition summaries (purple); these ranges are descriptive, not uncertainty intervals. Positive values indicate higher SpO2 than the arterial endpoint. The dashed zero line denotes equal readings, not a validated clinical acceptance margin. Hansen 1987 and Rice 1999 were graphical or numerically nonrecoverable, and Benoit 1997 used a post hoc corrected index; no pooled estimate, weight, or heterogeneity statistic is shown. Machine-readable source data are provided in Supplementary File S6.

Four clinical studies reported one peak or maximal-exercise pair per analyzed participant. Ascha et al. reported a sign-harmonized mean paired difference of +2.63 percentage points among 631 peak tests (SD, 2.75; limits of agreement, −2.9 to +8.1) [13]. Swigris et al. reported +1.50 among 83 adults with systemic-sclerosis-associated interstitial lung disease (SD, 4.00) [14]. Lundgren et al. reported −0.88 among 67 beryllium-sensitized adults (SD, 4.60) [31]. Campedelli et al. reported −0.44 among 268 complete peak pairs after sign harmonization, with a 95% confidence interval of −1.31 to +0.42 [39]. The source describes a confidence interval of the mean but also refers to it as a limit of agreement; we retained it as a mean confidence interval and did not use it as an individual agreement interval. Assuming an ordinary paired t interval gives a conditional SD of approximately 7.19 percentage points. Without that reconstruction, the three reported SDs range from 2.75 to 4.60, rather than 2.75 to 7.19. Retaining all four reported means leaves the bias range unchanged at −0.88 to +2.63. Thus, the reconstruction materially affects the upper dispersion estimate, but is not needed to demonstrate biases in both directions. Figure 3 distinguishes these quantities; none is a pooled interval.

Five historical exercise-only studies contributed repeated or correlated device comparisons. McGovern et al. reported −1.70 points (SD, 2.90) across 49 exercise pairs from eight participants with severe COPD [30]. Across concurrent or overlapping configurations, Martin et al. reported −0.59 to +0.87, and Barthélémy et al. reported −3.26 to +0.89 [29,36]. Powers et al. reported −2.77 to −0.87 at highest work, whereas Ries et al. reported +0.30 to +0.90 [27,37]. Multiple devices shared participants, and repeated-pair covariance was unavailable. Martin and Powers also targeted fractional oxyhemoglobin; these rows therefore remained within study clusters.

Four indirect studies reported summaries that mixed rest, exercise, recovery, altitude, oxygenation, or threshold-defined conditions. Standardized means extended from under-reading to over-reading. At simulated 4000 m, two ear devices yielded −4.8 and +9.8; another experiment reported −2.0 to +0.3 across three configurations [28,32]. Carone et al. reported −0.67 with limits of agreement from −6.88 to +5.55 across combined treadmill time points [35]. The agreement denominator was not reported. Roberts et al. combined 65 repeated rest, exercise, and recovery pairs from eight athletes [40]. Three further studies were supportive rather than core: Hansen and Rice provided graphical or numerically nonrecoverable comparisons, whereas Benoit analyzed a post hoc corrected rather than raw SpO2 index [33,34,38]. Complete device- and condition-level results are provided in Supplementary File S2 (e-Table S4).

Individual disagreement was more clinically informative than group mean bias in several reports. At peak exercise, 78 of 631 participants (12.4%) had SpO2 at least five points above SaO2 [13]. Four of 83 systemic-sclerosis participants had SpO2 above 88% despite SaO2 of 88% or lower [14]. Roberts et al. reported a near-zero aggregate mean (+0.1) but limits from −3.7 to +3.9 across repeated observations [40]. These findings illustrate study-specific threshold discordance; they do not define a universal 88% interchangeability margin.

Handling of repeated measures, multiple devices/sites and mixed periods was Yes in three studies, No in nine and Unclear in four in the retained author-agreed item record. These responses describe the reported methods; a No is not uniformly equivalent to demonstrated bias. Other limitations include changing device/site configurations, corrected index values and incompletely specified functional or fractional arterial targets. Three peak/maximal cohorts had reported paired SDs, and one had only a conditional reconstruction. Their compatibility is detailed in Supplementary File S4. The exploratory reported-SD analysis of three peak/maximal cohorts gave a mean bias of 1.14 percentage points (modified Hartung–Knapp 95% CI −3.29 to 5.57; Q-based I^2^ 95.3%). Adding the conditional Campedelli SD gave 0.75 (95% CI −1.90 to 3.41; I^2^ 96.4%). These small-k estimates are post hoc, clinically heterogeneous and dependent on reported cohort denominators; they do not resolve saturation-construct ambiguity or identify individual limits of agreement. The four-study result additionally assumes an ordinary paired-mean confidence interval for the 2024 reconstruction. No common clinical correction is recommended (Supplementary File S8).

Evidence interpretation remains limited by nonexchangeable measurement contexts and incompletely reported variance or dependence. Escourrou and Orenstein were excluded because adult data were inseparable [22,23]. No request for separable adult data was made to these authors. Their post hoc qualitative consideration did not establish a compatible stratum: Escourrou lacked exercise-only signed bias with paired variance; Orenstein remained a mixed-age repeated-measure cohort with correlated device estimates (Supplementary File S1). These exclusions may reduce completeness. The evidence neither establishes cross-context interchangeability nor proves universal device failure.

## 4. Discussion

The findings are twofold. First, the available comparisons do not establish that a single correction or substitution rule transfers across devices, sites, populations, oxygenation ranges, and arterial endpoint constructs. Signed mean differences occurred in both directions. Excluding the conditional SD reconstruction reduces the reported dispersion range but does not alter those mean differences or the published limits of agreement in Ascha et al. [13]. Second, reports rarely described the optical and processing architecture in enough detail to attribute between-study differences to a specific feature. The reporting audit is exploratory and cannot rank technologies. These limitations do not imply that every contemporary pulse oximeter fails in every exercise setting.

Mean accuracy and individual agreement answer different questions. Ascha et al. reported a mean difference of +2.63 points but limits of agreement from −2.9 to +8.1 [13]. Carone et al. reported a mean of −0.67 with limits from −6.88 to +5.55 [35]. Roberts et al. combined a near-zero aggregate mean with limits from −3.7 to +3.9 [40]. A small average difference can therefore coexist with important uncertainty near a decision boundary. Bland–Altman principles and later reporting guidance support retaining paired dispersion and genuine limits of agreement rather than treating correlation or mean bias as interchangeability [9,10,11,12].

### 4.1. Technology-Related Interpretation

Pulse oximetry is a measurement chain rather than a single sensor value. Emitter wavelengths and optical geometry determine tissue interrogation; site, contact, ambient light, and perfusion shape the acquired photoplethysmographic signal; motion suppression, averaging, calibration, and proprietary algorithms determine the displayed SpO2 [1,2,3,4,5,6,7,8,41,42,43]. Exercise perturbs several links simultaneously. A device name alone therefore cannot identify the operative configuration or processing version.

Several included studies made individual parts of this chain visible. Yamaya et al. compared forehead and digit probes under identical Nellcor signal-detection algorithms and used synchronized acquisition and signal-quality criteria [32]. Barthélémy et al. compared eight finger oximeters and recorded invalid pulse waves or display failures [36]. Other studies described headbands, splints, cable restraint, ambient-light shielding, ear rubbing, or signal-quality thresholds [27,28,32,33,34,35,37,38,40]. These controls are informative, but no report covered every domain. Device, site, cohort, oxygenation, and handling usually changed together, which prevents causal attribution of disagreement to one technology feature.

The audit exposed a reporting problem rather than proof that a feature was absent. No included report supplied numerical emitter wavelengths. Only two fully described averaging or update behavior, three provided sufficient algorithm or post-processing detail, and seven fully quantified signal failure or missingness. External manuals, patents, and assumptions based on model names were deliberately excluded because they may not match the hardware, software, firmware, or settings used in the original experiment. Reporting completeness must not be interpreted as performance quality.

Applicability to current devices and diverse patients remains uncertain. Much of the evidence used legacy equipment, small selected cohorts, and incomplete demographic reporting. Controlled and clinical evidence shows that pigmentation-related bias can vary with oxygenation range, probe type, and device [18,44,45,46,47,48,49]. The exercise literature cannot estimate that modifier. The US Food and Drug Administration’s January 2025 draft guidance remains nonbinding and emphasizes objective skin-pigmentation assessment and reporting of device configuration, motion, sensor site, and low-perfusion performance [19]. These priorities reinforce the identified gaps but cannot supply missing exercise data.

### 4.2. Implications for Optical Biosensor Design and Validation

At minimum, exercise-validation reports should identify manufacturer, model, hardware or software version, optical geometry, emitter wavelengths, detector arrangement, anatomical site, attachment and ambient-light control, averaging or update interval, motion-filtering strategy, perfusion or signal-quality thresholds, site-substitution rules, calibration or algorithm version, and every failed or missing reading. These descriptors should be linked to each device–site–population combination and to the analyzed arterial pair rather than reported only for the study as a whole.

Validation should proceed in stages. Bench and controlled-hypoxia work can isolate optical design and oxygenation range; standardized motion and perfusion challenges can characterize artifact handling; dynamic-exercise studies must then test the complete wearable system against simultaneous arterial co-oximetry [41,42,43,50]. Current devices should be evaluated in diverse, prospectively enrolled adults with objective skin-pigmentation measurement, prespecified site substitutions, versioned algorithms, and complete failure accounting. Repeated observations and multiple devices require multilevel or repeated-measures models. Reports should provide paired bias, paired SD, genuine limits of agreement, threshold discordance, and device-specific results.

The review did not evaluate the effectiveness of a clinical troubleshooting pathway, validate trend monitoring, or establish thresholds for arterial confirmation. The agreement findings therefore should not be converted into a clinical decision algorithm. Their implication is narrower: interpretation of reported exercise agreement requires attention to the measurement context and the distinction between average bias and individual disagreement.

### 4.3. Strengths and Limitations

Strengths include a strict direct-reference criterion, three updated databases, citation searching, independent duplicate screening and full-text assessment, dual-reviewer extraction and appraisal, sign harmonization, and retention of failed or numerically nonrecoverable outcomes. Study-level organization made repeated outcomes visible, and compatibility tables documented the original decision not to pool; neither establishes that all possible pooled analyses would be misleading. The exploratory reporting audit adds source-located, study-level coding of 10 technology domains and makes every reported, partial, and NR judgment inspectable.

Limitations include retrospective protocol freezing, no prospective registration, eight unretrieved reports and six Embase article records unavailable for screening because of export failure. The eligibility of those six records is unknown, so the supplementary search cannot be claimed as fully screened. Scopus and CINAHL were not searched, and unique reports may remain missed. Reviewer-level disagreement records were not retained. The technology audit was added after editorial pre-check and is explicitly post hoc and descriptive, not a change to primary eligibility or a validated device-performance score. The review-specific appraisal is not externally validated, so raw item evidence replaces aggregate risk and certainty grades. The exploratory mean-bias analyses include only three or four heterogeneous cohorts and cannot characterize individual agreement. The conditional 2024 SD assumes an unconfirmed interval-construction method; excluding it does not resolve other limitations. Mixed-age adult data were not requested from authors. AI-assisted technical work remained subordinate to both authors’ source checks and final decisions.

## 5. Conclusions

Across 16 studies, exercise SpO2 both overestimated and underestimated arterial co-oximetry endpoints, and small mean differences did not ensure close individual agreement. Post hoc peak/maximal mean-bias analyses were imprecise and clinically heterogeneous; they do not establish a transferable correction or individual interchangeability. The descriptive technology audit identified incomplete optical, processing and failure-reporting information. Final interpretation remains constrained by the search-accounting and methodological limitations described above.

The available evidence does not establish a universal correction factor nor a transportable substitution rule or device ranking. Future studies should pair simultaneous arterial co-oximetry with complete, versioned reporting of the emitter–detector system, site and fixation, signal averaging, motion and perfusion handling, algorithms, site substitutions, and failed readings. Such reporting would improve assessment of whether observed differences reflect sensor design, participant characteristics, or study procedures.

## Figures and Tables

**Table 1:** Included studies and source-based methodological limitations. Adult direct-co-oximetry eligibility is distinguished from compatibility for quantitative synthesis.

Study/Reference	Population and Timing	Pulse-Oximeter/Site	Arterial Endpoint	Principal Limitation; Full Evidence in Supplementary Files S2 and S3
Ascha 2018 [13]	Mixed clinical cardiopulmonary exercise testing referrals; 751 enrolled, 631 complete peak pairs; incremental cycle; peak	Nonin Avant 4000; forehead; contemporary	ABL800 co-oximetry	Complete-pair restriction could affect representativeness, but the retained outcome is one contemporaneous peak pair per analyzed participant.
Swigris 2009 [14]	Systemic-sclerosis-associated interstitial lung disease; *n* = 83; maximal cycle; maximum exercise	Changing devices; mostly finger, earlobe backup; mixed legacy era	Arterial co-oximetry	Device/site heterogeneity precludes a stable index-test estimate even though the reference is direct.
Zeballos 1991 [28]	Healthy Black male recruits; *n* = 33; multistage cycle at sea level/2300/4000 m; rest, exercise, recovery combined	HP 47201A and Biox IIA; earlobe; historical	IL282 functional SaO2	No study outcome is restricted to the protocol’s exercise-only analytical unit.
Yamaya 2002 [32]	Healthy, athlete, and clinical groups; *n* = 19; maximal cycle in normoxia/hypoxia; some hyperoxia	Three configurations; forehead/finger; historical	IL682 co-oximetry	The all-data primary estimates cannot be treated as independent participant-level observations.
Hansen 1987 [33]	Consecutive cardiopulmonary referrals; *n* = 14; incremental cycle; serial through 2 min recovery	Biox IIA; earlobe; historical	IL282 co-oximetry	Non-extractability limits precision synthesis but does not itself establish biased measurement.
Benoit 1997 [34]	Healthy men; *n* = 10; constant-load cycle in normoxia/hypoxia	Datex Satlite; earlobe; post hoc corrected index; historical	OSM3 co-oximetry	Retained only as technology-history/sensitivity evidence; not valid for the raw-SpO2 primary comparison.
McGovern 1996 [30]	Severe COPD; *n* = 8; progressive cycle; exercise pairs	Ohmeda 3700; finger; historical	IL482 co-oximetry	The raw device result is eligible, but repeated pairs cannot be treated as 49 independent observations.
Carone 1997 [35]	COPD on long-term oxygen therapy; *n* = 20; repeated 6 min treadmill walks with oxygen titration	Biox 3760; earlobe; historical	IL282 co-oximetry	The 47 sixth-minute clinical-cutoff pairs are not the Bland–Altman agreement denominator.
Martin 1992 [29]	Elite cyclists; *n* = 11; high-intensity/incremental cycle; exercise-only subset	Three Ohmeda 3740 units; ears/finger; historical	IL282 fractional HbO2	Direct fractional HbO2 is not silently interchangeable with functional SaO2 for the review’s primary target.
Barthélémy 1990 [36]	Clinical exercise laboratory; *n* = 57; moderate cycle; exercise-only	Eight devices; finger; historical	OSM3 functional SaO2	No prespecified single index device and no within-subject covariance are available.
Powers 1989 [37]	Healthy men; *n* = 10; incremental cycle in normoxia/hypoxia; highest work	Ohmeda/Biox; finger/ears; historical	IL282 fractional HbO2	A condition-specific estimate is structurally usable only after choosing one configuration; fractional HbO2 creates a reference-definition applicability concern.
Ries 1985 [27]	Pulmonary-laboratory patients; *n* = 116 total; treadmill/cycle across overlapping phases; exercise-only rows	HP 47201A and Biox IIA; earlobe; historical	IL282 co-oximetry	Exercise-only direction is available, but the variance and independence conditions for pooling are not.
Rice 1999 [38]	Competitive cyclists; *n* = 15; incremental cycle; serial exercise	Criticare US-504; earlobe; historical	ABL520 saturation measure	External technical verification supported eligibility; the article’s graphical result remains qualitative.
Lundgren 2001 [31]	beryllium sensitization/chronic beryllium disease referrals; *n* = 168; incremental cycle; maximum exercise	Two device generations; earlobe; mixed historical era	Two co-oximeter platforms	The beryllium sensitization estimate has a paired SD, but an unstratified mixed index technology limits a common target effect.
Campedelli 2024 [39]	Consecutive invasive-cardiopulmonary exercise testing referrals; 296 enrolled, 268 complete peak pairs; upright ramp cycle; peak primary	Stryker system; finger with earlobe/forehead fallback; contemporary	ABL90 Flex Plus co-oximetry	The point estimate is usable descriptively; the reported spread is not treated as a trusted paired-difference variance.
Roberts 1999 [40]	Athletes; 20 parent, 8 arterial-validation participants; supramaximal cycle; rest/exercise/recovery	Nellcor N200; forehead; historical	IL482 co-oximetry	The minimum-SaO2 row is conditionally defined and must not be combined independently with the aggregate or threshold rows.

## Data Availability

Data, unchanged author-agreed appraisal responses, source locations, extraction records, technology coding and exploratory analysis inputs are available in Supplementary Files S1–S8. Supplementary File S6 contains machine-readable source tables and provenance records; it does not contain participant-level data or copyrighted full texts. The Embase audit explicitly distinguishes supplied export records, platform counts and the author-confirmed screened subset.

## Supplementary Materials

The following supporting information can be downloaded at: https://www.mdpi.com/article/10.3390/bios16100565/s1, Supplementary File S1. Supplementary methods and search strategies. File name: Supplementary_S1_Methods.pdf. Format: PDF. e-Appendices S1–S4: exact original and post hoc Embase searches, citation-search methods, review-defined appraisal and synthesis rules, and mixed-age sensitivity; independent supplementary references. Supplementary File S2. Search, screening, extraction and descriptive methodological appraisal. File: Supplementary_S2_eTables_S1–S6.pdf. e-Tables S1–S6 contain the original search strategies, 34 full-text decisions, eight unretrieved reports, 16 studies, 46 outcomes and 192 unchanged author-agreed item responses with evidence locations. Supplementary File S3. Methodological appraisal codebook and interpretive limits. File: Supplementary_S3_Methodological_Appraisal.pdf. Describes item functions and source-based concerns without validated aggregate risk grades. Supplementary File S4. Compatibility, synthesis and interpretation limitations. File: Supplementary_S4_eTables_S7–S10.pdf. No formal overall certainty grades are assigned. Supplementary File S5. Citation-search disposition summary. File name: Supplementary_S5_Citation_Summary.pdf. Format: PDF. e-Table S11: disposition of 1506 citation-graph records, including 1342 new records screened. Supplementary File S6. Machine-readable source tables and provenance archive. File: Supplementary_S6_Source_Data.zip. Includes current table data, figure sources, author-agreed historical appraisal records, Embase accounting and exploratory analysis inputs, code and checksums. Historical aggregate categories are not current synthesis grades. Supplementary File S7. Technology-reporting audit. File name: Supplementary_S7_Technology_Audit.pdf. Format: PDF, with an accompanying machine-readable CSV. Study-level Reported, Partial, and NR codes for device/model, optical geometry, sensor site, wavelengths, averaging or update behavior, motion handling, perfusion or signal quality, fixation or ambient-light control, algorithm or post-processing detail, and signal failure or missingness; extracted details, evidence locators, and audit notes. e-Table S12: Domain-level reporting summary. Counts of Reported, Partial, and Not Reported (NR) classifications across 10 technology-reporting domains in the 16 included studies, with operational coding criteria. e-Table S13: Study-level status matrix. Reporting status for each of the 10 domains in each included study, comprising 160 study-domain judgments. R = Reported; P = Partial; NR = Not Reported. e-Table S14: Source-located technology extraction. Extracted device, sensor-site, and technology-reporting details for the included studies, with source-text locators and audit notes. Unreported properties were not inferred from device model names or external materials. Supplementary File S8. Post hoc mean-bias sensitivity analysis and supplementary-search accounting. File: Supplementary_S8_Revision_Analyses.pdf. Assumptions, small-k estimates, conditional SD reconstruction and the six-record export failure are reported explicitly.

## List of Abbreviations

COPD, chronic obstructive pulmonary disease; GRADE, Grading of Recommendations Assessment, Development and Evaluation; HbO2, fractional oxyhemoglobin relative to total hemoglobin; NR, not reported; PRISMA, Preferred Reporting Items for Systematic Reviews and Meta-Analyses; QUADAS-2, Quality Assessment of Diagnostic Accuracy Studies 2; SaO2, source-reported arterial oxygen saturation; SD, standard deviation; SpO2, peripheral oxygen saturation measured by pulse oximetry; SWiM, Synthesis Without Meta-analysis.
