# Supplementary material for: Pulse Oximetry Versus Arterial Co-Oximetry During Exercise: A Systematic Review of Agreement and Sensor Technology Reporting Gaps
Source: Biosensors (Basel). 2026 Oct 9;16(10):565. doi: 10.3390/bios16100565 (PMC13702209; doi:10.3390/bios16100565)
Supplement: Supplementary file 1 [file biosensors-16-00565-s001.zip › Supplementary_S1_Methods.pdf]

Supplementary File S1. Supplementary methods

Pulse Oximetry Versus Arterial Co-oximetry During Exercise: A Systematic Review of Agreement and Sensor-Technology Reporting Gaps

Shu Xie; Xueming Wen

Revision methods addendum. All deviations from the original synthesis are identified; no prospective registration is claimed.

Supplementary Methods

Eligibility and estimand

Eligible reports included adults aged 18 years or older and provided a paired comparison between pulse-oximeter-derived SpO_2 and a direct arterial co-oximetry oxygen-saturation endpoint during dynamic exercise or within two minutes after exercise cessation that could be interpreted quantitatively or qualitatively. Reports based only on calculated saturation, blood-gas values without confirmed co-oximetry, arterialized/capillary/venous blood, rest-only measurements, or no interpretable paired exercise-window comparison were excluded. Mixed-age reports were eligible only when adult results were separable. Reports without recoverable numerical agreement statistics were retained only for qualitative synthesis; post hoc corrected indices were retained only as secondary technology-history evidence. This reporting clarification is documented in protocol amendment v0.6 and did not change the included set.

Agreement was sign-harmonized as SpO_2 minus the source-reported arterial co-oximetry oxygen-saturation endpoint in percentage points. Negative values indicate pulse-oximeter under-reading relative to the arterial reference. SaO_2 was used where a source reported arterial saturation in those terms; fractional HbO_2 was named separately. When a source

reported the opposite sign direction, its sign was reversed while the reported SD or limits of agreement were retained on the same scale. Confidence intervals, SD-based intervals, and limits of agreement were not relabeled as one another.

Database searching and screening

PubMed, Web of Science Core Collection, and SPORTDiscus with Full Text were searched without language, year, or document-type restrictions and updated to a common end date of August 30, 2026. A post hoc Embase search was performed on 29 September 2026, with an author-confirmed export failure affecting six article records, which were not available for screening. Complete original strategies, Embase syntax, source counts, and file-integrity evidence are provided in e-Appendix S1.

The original three-database searches identified 4171 records. Automated deduplication removed 713 records and three later-linked duplicate or variant reports were removed before study counting, leaving 3455 records for title/abstract review. Of these, 3413 were excluded and 42 reports were sought. Eight reports were not retrieved, 34 were assessed, 18 were excluded, and 16 studies were included. A report not retrieved was not assigned a full-text exclusion reason and remains of unknown eligibility. Complete citations, documented legal/open/institutional route checks, routes not completed, and plausible impacts are reported in e-Table S2, Part B. Four reports were prioritized for interlibrary loan, but no request was submitted and authors were not contacted before the retrieval phase closed. A further PubMed and University of Adelaide repository check on September 1, 2026, located citation and abstract metadata for Wood et al. but no lawful full report. Its abstract describes maximal cycling with arterial co-oximetry and could plausibly alter the peak/maximal evidence set if later retrieved and eligible.

Two reviewers independently screened all 3455 database records against the same eligibility criteria and independently assessed the 33 originally retrieved full-text reports. Disagreements were resolved by discussion and the consensus disposition was entered in the final screening record. AI-assisted triage was used to organize records and propose dispositions but did not replace either reviewer's final judgment. Reviewer-level disagreement counts were not retained, so kappa and percentage agreement were not calculated. The later Chailleux retrieval and E04 exclusion were confirmed by both authors on 30 September 2026; the revised assessed total is 34.

Supplementary citation searching

Backward and forward citation searching was completed on August 31, 2026, using 21 resolved seeds. It identified 1506 unique citation-graph records, of which 164 were already represented in the database corpus and 1342 were new records. Two reviewers independently screened the 1342 new records; the consensus disposition excludes all of them at title/abstract review and identifies no additional report for retrieval. Methods, yields, exclusions, limitations, and hashes are provided in e-Appendix S2. AI assistance is disclosed, but the final dispositions were made by the two human reviewers.

Full-text decisions and extraction safeguards

We retain source-based item evidence rather than validated continuous-agreement risk grades.

The primary dataset contains 16 study rows and 46 device/condition-level outcome rows. The latter are correlated or conditional observations rather than 46 independent study effects. Nine studies provided core numerical results attributable to the exercise window (G1 and G2), four provided indirect summaries mixing exercise with other periods or conditions (G3), and three provided qualitative or secondary-index evidence (G4).

Methodological appraisal, synthesis, and certainty

Aggregate any-No concern categories are not used in the current synthesis. The unchanged historical records remain available for provenance.

Post hoc mixed-age sensitivity

Escourrou et al. (1990) and Orenstein et al. (1993) remained excluded because adult-only results were inseparable. Qualitative sensitivity analysis did not create a compatible pooling stratum and did not alter the central conclusion. Details are provided in e-Appendix S4.

e-Appendix S1. Complete Database Search Strategies

Search coverage and updates

The strategies below reproduce the frozen searches and their common-date update through 2026-08-30. All three databases were rerun without date, language, or document-type restrictions. PubMed added three records; Web of Science Core Collection and SPORTDiscus with Full Text showed no count increase.

PubMed

- Initial search date: 2026-08-24
- Initial export: 2312 unique PMIDs
- Update date: 2026-08-30
- Update result: 2315 PMIDs; three added, zero original PMIDs absent
- Filters: none

```
("Oximetry"[Mesh] OR oximetr*[tiab] OR "oxygen saturation"[tiab] OR SpO2[tiab])
AND ("Exercise Test"[Mesh] OR "Exercise"[Mesh] OR "Physical Exertion"[Mesh] OR
exercis*[tiab] OR exert*[tiab] OR "walk test"[tiab] OR "walk tests"[tiab] OR
treadmill*[tiab] OR cycl*[tiab] OR ergomet*[tiab] OR CPET[tiab])
AND ("Blood Gas Analysis"[Mesh] OR cooximetr*[tiab] OR "co-oximetr*"[tiab] OR
SaO2[tiab] OR "arterial oxygen saturation"[tiab] OR "arterial saturation"[tiab]
OR "arterial blood"[tiab] OR "arterial blood gas"[tiab] OR "arterial blood
gases"[tiab] OR "arterial sample"[tiab] OR "arterial samples"[tiab] OR "arterial
catheter"[tiab] OR "arterial catheterization"[tiab] OR "arterial line"[tiab])
```

The NBIB export did not embed the expression. The exact query was recovered from the dated original result URL and validated by reproducing all 2312 original PMIDs.

Web of Science Core Collection

- Editions: All
- Initial search date: 2026-08-24
- Initial result/export: 622 records
- Update date: 2026-08-30
- Update result: 622 records
- Filters: none

```
TS=(oximetr* OR "oxygen saturation" OR SpO2)
AND TS=(exercis* OR exert* OR "walk test" OR "walk tests" OR treadmill* OR cycl*
OR ergomet* OR CPET)
AND TS=(cooximetr* OR "co-oximetr*" OR SaO2 OR "arterial oxygen saturation" OR
"arterial saturation" OR "arterial blood" OR "arterial blood gas*" OR "arterial
sample*" OR "arterial catheter*" OR "arterial line")
```

SPORTDiscus with Full Text via EBSCOhost

- Initial search/export date: 2026-08-29
- Initial result/export: 1234 records
- Update date: 2026-08-30
- Update result: 1234 records; no count increase
- Filters: none recorded; publication date, language, and document type were unrestricted

```
TX ("pulse oximetr*" OR "pulse oximeter*" OR oximetr* OR "oxygen saturation" OR
SpO2)
AND TX (exercis* OR exert* OR "walk test*" OR treadmill* OR cycl* OR ergomet* OR
CPET OR "cardiopulmonary exercise test*")
AND TX (cooximetr* OR "co-oximetr*" OR SaO2 OR "arterial oxygen saturation" OR
"arterial saturation" OR "arterial blood" OR "arterial blood gas*" OR "arterial
sample*" OR "arterial catheter*" OR "arterial line")
```

Source-file integrity

**Source export**	**Records**	**SHA-256**
PubMed NBIB export	2312	5A2F8278645982C92B8BAF79E69DFAD10BCB6F132 A06A60B807C7515A79EE6AC
Web of Science export, records 1-500	500	3AF7D79C59C1132676B1B190D27F20A00A034A19E 9E7DDDE1F39F6430893C32D

Source export	Records	SHA-256
Web of Science export, records 501-622	122	ACD9A9392D408574C4F57F4BF829CD5B115CC9EB B3EC28D338220FD046F63C8C
SPORTDiscus RIS export	1234	EEAA0CF8045562F4E33423E436E0325442B9C79F01 A90194CAC5DD5C7686B15D

Post hoc Embase search and accounting boundary

Search date: 2026-09-29. Embase.com, advanced search. The narrower #225 syntax was: ('pulse oximetry'/exp OR oximetr*:ti,ab,kw OR oxymetr*:ti,ab,kw OR spo2:ti,ab,kw OR 'oxygen saturation':ti,ab,kw) AND ('exercise'/exp OR exercis*:ti,ab,kw OR exert*:ti,ab,kw OR treadmill*:ti,ab,kw OR cycl*:ti,ab,kw OR ergomet*:ti,ab,kw OR cpet:ti,ab,kw OR 'walk test*':ti,ab,kw) AND ('blood gas analysis'/exp OR cooximetr*:ti,ab,kw OR 'co-oximetr*':ti,ab,kw OR sao2:ti,ab,kw OR 'arterial blood':ti,ab,kw OR 'arterial sample*':ti,ab,kw OR 'arterial saturation':ti,ab,kw).

The final supplementary Embase search (#225, 29 September 2026) displayed 1196 article records. The supplied article export contained 1190 recoverable unique identifiers. The authors confirmed that the six-record shortfall resulted from an export failure. These six records were not available for screening and were not counted as duplicates, title/abstract exclusions or full-text exclusions. The authors confirmed completion of screening of the supplied records. Search #207 is retained only as audit history and is not added to the final search denominator. The 231 clinical-trial registry records are reported separately from article records. A broader mapped variant (#207) displayed 1255 articles and 263 trial records; its RIS held 1247 complete article IDs and 262 unique trial identifiers. A cross-export union recovered 1254 unique article identifiers. These historical nonidentical-query counts are not final #225 yields and are not summed.

The supplied 725-record provisional unmatched queue was reported by both authors as excluded after title/abstract screening. Bookkeeping identified nine links to the original

corpus and five within-queue duplicate records, leaving 711 distinct new records in that queue. Seven other report-distinct records, absent from the 725-row queue, were also reported excluded by both authors. The resulting 718 provisional exclusions describe only this audited subset, not all Embase records; they are not full-text exclusions. The separate reviewer-level decision forms were not supplied. Figure 1 retains the original three-database and citation-search flow. Supplementary Embase accounting is reported separately, including the six author-confirmed export failures. These are export-stage losses, not unretrieved full-text reports or human screening exclusions.

e-Appendix S2. Backward and Forward Citation Searching

Search date and scope

Backward and forward citation searching was completed on August 31, 2026. The seed set comprised 17 provisionally included reports, one subsequently excluded after full-text reference-method reassessment, two historical exercise-specific reviews, one broad adult pulse-oximetry meta-analysis, and one 2024 systematic review of skin-tone-related pulse-oximetry performance. OpenAlex was used only to discover citation relationships; its metadata were not used to establish study eligibility.

The 21 seeds were:

- 17 provisional study reports; the final included table has 16;
- Mengelkoch et al. (1994) [1];
- Gaskin and Thomas (1995) [2];
- Jensen et al. (1998) [3]; and
- Martin et al. (2024) [4].

Discovery and reconciliation

For each resolved seed, cited references and later citing records were retrieved. Records were deduplicated by OpenAlex work identifier and reconciled against the frozen database corpus by normalized DOI followed by exact normalized title. Two reviewers independently screened records not already represented in the database corpus under the unchanged title/abstract eligibility criteria and resolved disagreements by discussion. Citation-search records were retained as a separate PRISMA “other methods” branch and did not change the database-search denominator. The machine-readable disposition identifies the two-reviewer consensus as the final reviewer status and retains AI assistance only as triage/reconciliation provenance.

Yield

Stage	No.
Citation-search seeds	21
Seeds unresolved	0
Unique citation-graph records	1506
Backward only	445
Forward only	1006
Found in both directions	55
Reconciled to database corpus by DOI	145
Reconciled by exact title	19
New records screened	1342
New records excluded at title/abstract review	1342
Additional reports sought	0
Additional eligible studies	0

The comprehensive disposition comprised 17 provisional reports (16 ultimately included and one later excluded), 145 existing exclusions, two reports already classified as not retrieved, and 1342 outside-corpus title/abstract exclusions. Citation searching recovered all 17 provisional reports; 16 remained included after the later reference-method reassessment. The two not-retrieved records were Scoggin et al. (1977), Clinical evaluation of a new ear

oximeter, [5] and Wood et al. (1997), Accuracy of two pulse oximeters during maximal cycling exercise. [6] Their eligibility remains unknown because a lawful full report was not obtained.

Outside-corpus exclusions

Primary exclusion code	Reason	No.
E03_NO_ELIGIBLE_EXERCISE_TIMEPOINT	No eligible dynamic-exercise or immediate-postexercise comparison identified	461
E04_NO_PULSE_OXIMETRY_SPO2_INDEX	Pulse-oximeter SpO2 not identified as the index test	710
E05_NO_DIRECT_ARTERIAL_COOXIMETRY_REFERENCE	No paired direct arterial co-oximetry reference identified	34
E07_INELIGIBLE_REPORT_TYPE	Review, editorial, conference abstract, dissertation, or other ineligible report type	137
**Total**		**1342**

OpenAlex citation links and abstracts can be incomplete, and lexical triage can miss concepts absent from the available title or abstract. AI-assisted triage organized the queue but did not replace the two reviewers' independent decisions. The final consensus disposition promoted no record to full-text retrieval. Reviewer-level disagreement counts were not retained, so inter-rater agreement is not reported. This completed disposition does not remove the discovery limitations of OpenAlex or substitute for records that may be exclusive to databases not searched. A later Embase search was performed, but its incomplete exports were not merged into the original three-database flow.

Reproducibility hashes

Artifact	SHA-256
Citation-search seed registry	AE8E5C152D825842E49FF2DE8EC4D0A5FD519A68A95DD2CF3EA101E6ED78584E
Complete citation-graph discovery set	B68DC86AD8AACB7CD8C923CB0CB5B42204DD6E58A8CE0199CCCF6FFEDECD7BDC
Candidate screening set	383C585E5A8BFA23360DE680E73B966DB7C9A4ACDDAF584E424BAE2C4F60BBDA
Initial disposition ledger	4C9CFFEE59B0512CE7AAEA0DFFE68310B5D28E9846BAA322743A5CAB36E54B7F

Artifact	SHA-256
Complete two-reviewer consensus disposition	0737F6805D36CFD98674F5F5D6BD0052A12C42324940B8C7359A32D6214C3462

e-Appendix S3. Appraisal, Synthesis Compatibility, and Confidence

Review-specific methodological appraisal and tool-fit limits

Both authors checked and agreed the final 192 item responses for the 16 included studies. Separate independent rating forms were not retained. The historical 12-item framework drew on QUADAS-2 domains, but QUADAS-2 concerns diagnostic accuracy rather than continuous agreement. We therefore use the retained evidence as a descriptive methodological appraisal, distinguishing potential bias mechanisms, reporting or construct ambiguity, and eligibility checks. We do not calculate an overall risk-of-bias grade or use an any-No algorithm in the current synthesis. Missing reporting indicates uncertainty rather than proof of bias. Supplementary Files S2 and S3 provide the unchanged author-agreed responses, source locations and an interpretive codebook; original aggregate categories are retained only in the provenance archive. This descriptive framework is not an externally validated continuous-agreement appraisal tool.

The historical aggregation algorithm and its downstream confidence labels are archived, not current risk-of-bias or certainty assessments.

The complete signaling instrument and all 192 retained item-level responses are provided in e-Table S6.

Compatibility gate and decision not to pool

The primary synthesis describes paired mean differences in percentage points, paired SDs and individual limits of agreement where reported. The original review-specific compatibility

rule required three independent studies within a common clinical and measurement stratum; this is not a universal statistical requirement. SWiM-guided direction-and-magnitude synthesis avoids significance vote counting. We added a post hoc exploratory random-effects mean-bias analysis of three independent peak/maximal cohorts with reported paired SDs, and a four-cohort sensitivity analysis adding the conditional 2024 SD. One estimate per cohort was used; correlated devices, stages and overlapping subgroups were not treated as independent studies. Sampling variance was SD squared divided by the participant count, with REML between-study variance and modified Hartung–Knapp confidence intervals using $k-1$ degrees of freedom. Disease, device/site and arterial-construct differences limit the interpretation of the average. Pooling mean bias does not estimate individual agreement or a transferable correction factor. With only three or four heterogeneous cohorts, meta-regression and small-study tests were not performed. Supplementary File S8 reports inputs, assumptions and reproducible calculations.

Peak/maximal clinical compatibility addendum

All four peak/maximal studies had a reported or reconstructed paired SD, but this numerical threshold did not create a common target effect.

Study	Population	Device/site	Exercise time point	Reported arterial co-oximetry endpoint	Paired SD status	Decisive reason not combined
Ascha 2018	Mixed clinical cardiopulmonary exercise-testing referrals; 631 complete peak pairs	Nonin Avant 4000; forehead	Peak, final 1-2 minutes before stopping	ABL800 source-reported SaO2; functional/fractional construct not specified	2.75 percentage points	No set of three exchangeable device-site-population targets; contemporary forehead referral cohort differs from the other candidates

Study	Population	Device/site	Exercise time point	Reported arterial co-oximetry endpoint	Paired SD status	Decisive reason not combined
Swigris 2009	Systemic sclerosis-associated interstitial lung disease; 83 participants	Changing platforms; mostly finger with earlobe backup	Maximum exercise	Source-reported SaO2; functional/fractional construct not specified	4.00 percentage points	Changing index/reference platforms, site backup, altitude-specific context, and source-specific methodological limitations prevent a stable common target
Lundgren 2001	Beryllium-sensitized subgroup; 67 participants	Two historical generations; earlobe	Maximum exercise	Source-reported SaO2 from two co-oximeter generations; functional/fractional construct not specified	4.60 percentage points	Both index and reference platforms changed; the beryllium-sensitized earlobe target is not exchangeable with at least two other candidates
Campedelli 2024	Invasive cardiopulmonary exercise-testing referrals; 268 complete peak pairs	Stryker system; finger with earlobe/forehead fallback	Peak exercise	ABL90 Flex Plus source-reported SaO2/oxyhaemoglobin; functional/fractional calculation not explicitly defined	Approximately 7.19 percentage points, reconstructed from the reported 95% CI of the paired mean and n=268	Despite a recoverable paired SD, sensor-site fallback, a distinct invasive-exercise population, and an incompletely specified saturation construct prevent membership in a three-study exchangeable technical-clinical stratum

The same comparison is supplied as e-Table S8.

Structured synthesis groups

Group	Evidence structure	Studies	Principal reason not pooled
G1	Peak/maximal clinical cardiopulmonary exercise testing with one reported pair per participant	4	No pooled primary common target; post hoc mean-bias sensitivity is provided in Supplementary File S8.
G2	Historical exercise-only device comparisons with repeated observations	5	Within-study dependence, legacy devices/sites, mixed saturation definitions, missing covariance
G3	Mixed rest/exercise/recovery or condition-combined summaries	4	Primary exercise-window estimand not separable; repeated and condition-mixed data
G4	Qualitative or secondary-index evidence	3	Raw paired agreement not extractable or index was post hoc corrected

e-Appendix: Narrative interpretation of evidence limitations

For each synthesis context, consider the specific design and reporting limitations, inconsistent signed differences, clinical and reference-construct indirectness, and unavailable or imprecise variance estimates. No formal GRADE certainty category is assigned.

Group	Interpretation without formal certainty grades
G1	Three reported-SD peak cohorts, conditional fourth; different clinical/technology contexts and unknown arterial constructs. Exploratory mean-bias analysis is in Supplementary File S8.
G2	Repeated stages/devices with unrecovered covariance; device and saturation-construct indirectness.

G3	Mixed timing/conditions, uncertain exercise-only denominator and dependence.
G4	Graphical or corrected-index outputs; recoverable raw paired variance unavailable.

Failure to establish interchangeability is not proof that every device fails in every setting.

e-Appendix S4. Post Hoc Mixed-Age Sensitivity Analysis

The adult-only eligibility rule was unchanged. Two otherwise relevant reports were excluded because adult results could not be separated from results in participants younger than 18 years. They were examined qualitatively after internal peer review to test whether their inclusion could alter the no-pooling decision or the central interpretation.

**Report**	**Population and adult separability**	**Exercise-period evidence**	**Why it remains outside the primary synthesis**
Escourrou et al., 1990 [10]	101 pulmonary patients aged 17-81 years; number and results of minors not reported separately	Three devices in separate patient groups; combined rest/exercise difference range -9 to +16 percentage points and mean 1.1 ± 3.0, with direction insufficiently clear for safe conversion; rest-to-exercise change correlation was reported	No adult-only result; no device-specific exercise-only signed bias and paired-difference variance
Orenstein et al., 1993 [11]	Nine patients with cystic fibrosis; ages 13, 14, 15, 22, 23, 23, 23, 28, and 29 years; three minors and six adults, without adult-only estimates	At peak exercise, the sign-harmonized mean paired difference (SpO_2 - SaO_2) was +3.2 for Biox, +1.0 for Nellcor, and -0.3 percentage points for Hewlett-Packard; reported precision values were 7.1, 7.5, and 2.5, respectively	One mixed-age cohort with three correlated devices and 50 repeated pairs; missing readings; no adult-only estimates

Mixed-age reports were excluded when adult data were inseparable. No author request for separable data was made; qualitative discussion does not change formal eligibility.

This sensitivity analysis was post hoc and was checked and agreed by both authors.

Supplementary file map

e-Tables S1–S6 are in Supplementary File S2; the descriptive appraisal codebook is in Supplementary File S3; e-Tables S7–S10 are in Supplementary File S4; the citation ledger is in Supplementary File S5. Machine-readable source records are in Supplementary File S6; technology coding is in Supplementary File S7 and revision analyses/search accounting in Supplementary File S8.

Reproducibility and author-verification boundary

AI-assisted technical processing did not supply missing study methods, author responses or screening decisions. The authors supplied the agreed screening and appraisal decisions.

Supplementary references

1. Mengelkoch LJ, Martin D, Lawler J. A review of the principles of pulse oximetry and accuracy of pulse oximeter estimates during exercise. Phys Ther. 1994;74(1):40-49. doi:10.1093/ptj/74.1.40

2. Gaskin L, Thomas J. Pulse oximetry and exercise. Physiotherapy. 1995;81(5):254-261. doi:10.1016/S0031-9406(05)66819-8

3. Jensen LA, Onyskiw JE, Prasad NGN. Meta-analysis of arterial oxygen saturation monitoring by pulse oximetry in adults. Heart Lung. 1998;27(6):387-408. doi:10.1016/S0147-9563(98)90086-3

4. Martin D, Johns C, Sorrell L, et al. Effect of skin tone on the accuracy of the estimation of arterial oxygen saturation by pulse oximetry: a systematic review. Br J Anaesth. 2024;132(5):945-956. doi:10.1016/j.bja.2024.01.023

5. Scoggin C, Nett L, Petty TL. Clinical evaluation of a new ear oximeter. Heart Lung. 1977;6(1):121-126.

6. Wood RJ, Gore CJ, Hahn AG, et al. Accuracy of two pulse oximeters during maximal cycling exercise. Aust J Sci Med Sport. 1997;29(2):47-50.

7. Whiting PF, Rutjes AWS, Westwood ME, et al. QUADAS-2: a revised tool for the quality assessment of diagnostic accuracy studies. Ann Intern Med. 2011;155(8):529-536. doi:10.7326/0003-4819-155-8-201110180-00009

8. Campbell M, McKenzie JE, Sowden A, et al. Synthesis without meta-analysis (SWiM) in systematic reviews: reporting guideline. BMJ. 2020;368:l6890. doi:10.1136/bmj.l6890

9. Guyatt GH, Oxman AD, Vist GE, et al. GRADE: an emerging consensus on rating quality of evidence and strength of recommendations. BMJ. 2008;336(7650):924-926. doi:10.1136/bmj.39489.470347.AD

10. Escourrou PJ, Delaperche MF, Visseaux A. Reliability of pulse oximetry during exercise in pulmonary patients. Chest. 1990;97(3):635-638. doi:10.1378/chest.97.3.635

11. Orenstein DM, Curtis SE, Nixon PA, Hartigan ER. Accuracy of three pulse oximeters during exercise and hypoxemia in patients with cystic fibrosis. Chest. 1993;104(4):1187-1190. doi:10.1378/chest.104.4.1187

The primary synthesis describes paired mean differences in percentage points, paired SDs and individual limits of agreement where reported. The original review-specific compatibility rule required three independent studies within a common clinical and measurement stratum; this is not a universal statistical requirement. SWiM-guided direction-and-magnitude synthesis avoids significance vote counting. We added a post hoc exploratory random-effects mean-bias analysis of three independent peak/maximal cohorts with reported paired SDs, and a four-cohort sensitivity analysis adding the conditional 2024 SD. One estimate per cohort

was used; correlated devices, stages and overlapping subgroups were not treated as independent studies. Sampling variance was SD squared divided by the participant count, with REML between-study variance and modified Hartung–Knapp confidence intervals using k−1 degrees of freedom. Disease, device/site and arterial-construct differences limit the interpretation of the average. Pooling mean bias does not estimate individual agreement or a transferable correction factor. With only three or four heterogeneous cohorts, meta-regression and small-study tests were not performed. Supplementary File S8 reports inputs, assumptions and reproducible calculations.

Author-confirmed revision: Chailleux 1989, PMID 2740587, was subsequently obtained and excluded under E04_NO_PULSE_OXIMETRY_SPO2_INDEX. This is an author-reported full-text decision, not a new independent source verification by the assistant. The original search branch now has 42 reports sought, eight not retrieved, 34 assessed and 18 excluded; 16 studies remain included. Final supplementary Embase search: #225, 1196 articles. #207 is audit history, not an additional search denominator.
