# Supplementary material for: Pulse Oximetry Versus Arterial Co-Oximetry During Exercise: A Systematic Review of Agreement and Sensor Technology Reporting Gaps
Source: Biosensors (Basel). 2026 Oct 9;16(10):565. doi: 10.3390/bios16100565 (PMC13702209; doi:10.3390/bios16100565)
Supplement: Supplementary file 1 [file biosensors-16-00565-s001.zip › Supplementary_S2_eTables_S1–S6.pdf]

Supplementary File S2

Pulse Oximetry Versus Arterial Co-oximetry During Exercise: A Systematic Review of Agreement and Sensor-Technology Reporting Gaps

Shu Xie; Xueming Wen

e-Table S1. Search provenance and reproducible strategies

The three original databases were updated through August 30, 2026, without language, year, or document-type restrictions. The later Embase audit is detailed separately in Supplementary File S1 and is not part of this table's final-yield column.

Database	Platform/edition	Search dates	Final yield	Restrictions	Exact strategy	Export integrity
PubMed	National Library of Medicine	Initial: Aug 24, 2026; update: Aug 30, 2026	2,315	No language, year, or document-type restrictions	("Oximetry"[Mesh] OR oximetr*[tiab] OR "oxygen saturation"[tiab] OR SpO2[tiab]) AND ("Exercise Test"[Mesh] OR "Exercise"[Mesh] OR "Physical Exertion"[Mesh] OR exercis*[tiab] OR exert*[tiab] OR "walk test"[tiab] OR "walk tests"[tiab] OR treadmill*[tiab] OR cycl*[tiab] OR ergomet*[tiab] OR CPET[tiab]) AND ("Blood Gas Analysis"[Mesh] OR cooximetr*[tiab] OR "co-oximetr*"[tiab] OR SaO2[tiab] OR "arterial oxygen saturation"[tiab] OR "arterial saturation"[tiab] OR "arterial blood"[tiab] OR "arterial blood gas"[tiab] OR "arterial blood gases"[tiab] OR "arterial sample"[tiab] OR "arterial samples"[tiab] OR "arterial catheter"[tiab] OR "arterial catheterization"[tiab] OR "arterial line"[tiab])	Initial NBIB: 2,312 records; SHA-256 5A2F8278645982C92B8BAF79E69DFAD10BCB6F132A06A60B807C7515A79EE6AC; update added 3
Web of Science Core Collection	All editions	Initial: Aug 24, 2026; update: Aug 30, 2026	622	No language, year, or document-type restrictions	TS=(oximetr* OR "oxygen saturation" OR SpO2) AND TS=(exercis* OR exert* OR "walk test" OR "walk tests" OR treadmill* OR cycl* OR ergomet* OR CPET) AND TS=(cooximetr* OR "co-oximetr*" OR SaO2 OR "arterial oxygen saturation" OR "arterial saturation" OR "arterial blood" OR "arterial blood gas*" OR "arterial sample*" OR "arterial catheter*" OR "arterial line")	500-record batch SHA-256 3AF7D79C59C1132676B1B190D27F20A00A034A19E9E7DDDE1F39F6430893C32D; 122-record batch SHA-256 ACD9A9392D408574C4F57F4BF829CD5B115CC9EBB3EC28D338220FD046F63C8C
SPORTDiscus with Full Text	EBSCOhost	Initial: Aug 29, 2026; update: Aug 30, 2026	1,234	No language, year, or document-type restrictions	TX ("pulse oximetr*" OR "pulse oximeter*" OR oximetr* OR "oxygen saturation" OR SpO2) AND TX (exercis* OR exert* OR "walk test*" OR treadmill* OR cycl* OR ergomet* OR CPET OR "cardiopulmonary exercise test*") AND TX (cooximetr* OR "co-oximetr*" OR SaO2 OR "arterial oxygen saturation" OR "arterial saturation" OR "arterial blood" OR "arterial blood gas*" OR "arterial sample*" OR "arterial catheter*" OR "arterial line")	RIS SHA-256 EEAA0CF8045562F4E33423E436E0325442B9C79F01A90194CAC5DD5C7686B15D

e-Table S2, Part A. Full-text eligibility decisions

Thirty-three reports were assessed in the original review. The authors subsequently confirmed Mollard 2010 as a full-text exclusion under the direct arterial co-oximetry criterion; no separate second independent reassessment is claimed.

Record ID	Author-year	Title	Final decision	Primary exclusion reason	Decisive evidence note
PUBMED:0011	Ascha, 2018	Pulse Oximetry and Arterial Oxygen Saturation during Cardiopulmonary Exercise Testing.	Included	Not applicable	Full text confirms adult cardiopulmonary exercise testing on cycle ergometer, forehead SpO2, radial-artery ABG with ABL800 FLEX co-oximetry, measurements during the last 1-2 minutes before stopping, and paired exercise data with bias/limits of agreement.
PUBMED:0440	Brown, 1993	Re-examination of the incidence of exercise-induced hypoxaemia in highly trained subjects.	Excluded	E14_REFERENCE_STANDARD_NOT_CONFIRMABLE_ON_FULLTEXT	Full text confirms simultaneous arterial blood sampling and ear oximetry during cycling but does not identify a co-oximeter or another definitive direct arterial saturation method. The report did not establish that the arterial saturation endpoint was obtained by direct multiwavelength co-oximetry and was excluded under the protocol-defined reference-standard criterion.
PUBMED:0778	Swigris, 2009	Exercise peripheral oxygen saturation (SpO2) accurately reflects arterial oxygen saturation (SaO2) and predicts mortality in systemic sclerosis.	Included	Not applicable	Full text confirms adult systemic sclerosis-associated interstitial lung disease maximal cycle cardiopulmonary exercise testing, radial arterial line, concurrent finger/ear pulse oximetry, arterial blood drawn every minute and analysed with a co-oximeter, with signed SpO2-SaO2 exercise agreement data.
PUBMED:1345	Elkjær, 2024	Validity and reliability of measurement of peripheral oxygen saturation during the 6-Minute Walk Test in patients with systemic sclerosis.	Excluded	E14_REFERENCE_STANDARD_NOT_CONFIRMABLE_ON_FULLTEXT	Full text confirms an adult 6-minute walk test, an arterial line, and approximately 20-second postexercise paired results. It names an ABL800 blood-gas analyzer but does not explicitly establish co-oximetry. The report was excluded under the protocol-defined reference-standard criterion.
PUBMED:2309	Duknic, 2021	Comparison of Repetitive Cardiac Output Measurements at Rest and End-Exercise by Direct Fick Using Pulse Oximetry vs. Blood Gases in Patients With Pulmonary Hypertension.	Excluded	E12_PAIRED_METHOD_DATA_NOT_EXTRACTABLE	Full text compares cardiac-output methods (direct Fick using pulse oximetry versus blood gases), not an extractable paired SpO2-SaO2 method-comparison outcome; saturation data serve only as formula inputs.
PUBMED:0912	Alkhoury, 2021	Heart-rate tuned comb filters for processing photoplethysmogram (PPG) signals in pulse oximetry.	Excluded	E05_NO_DIRECT_ARTERIAL_COOXIMETRY_REFERENCE	Full text describes a motion-artifact filtering/algorithm study using synthetic signals and prototype dual-pulse-oximeter plus ECG recordings in 14 exercising adults; it contains no arterial sample, co-oximetry reference, or paired SpO2-SaO2 comparison.
PUBMED:0978	Williams, 2024	A 76-Year-Old-Man With Dyspnea and Abnormal Oximetry Run.	Excluded	E05_NO_DIRECT_ARTERIAL_COOXIMETRY_REFERENCE	Clinical case report gives a 6-minute-walk SpO2 nadir and separately describes diagnostic catheterisation oximetry; it provides no concurrent exercise arterial co-oximetry or paired SpO2-SaO2 method-comparison data.

Record ID	Author-year	Title	Final decision	Primary exclusion reason	Decisive evidence note
PUBMED:0028	Smyth, 1986	Ear oximetry during combined hypoxia and exercise.	Excluded	E10_REFERENCE_METHOD_NOT_COOXIMETRY_ON_FULLTEXT	Full text used arterial samples but analysed them on a Corning 175 blood-gas analyser whose saturation is calculated from blood-gas values rather than direct co-oximetry; the reference standard is ineligible.
PUBMED:0054	Zeballos, 1991	Reliability of noninvasive oximetry in black subjects during exercise and hypoxia.	Included	Not applicable	Thirty-three adult men performed steady-state cycle exercise under normoxia and hypoxia; radial arterial samples were concurrent with ear oximetry and analysed using an IL 282 co-oximeter; signed paired bias data are reported.
PUBMED:0060	Yamaya, 2002	Validity of pulse oximetry during maximal exercise in normoxia, hypoxia, and hyperoxia.	Included	Not applicable	Nineteen adults completed maximal cycle exercise under normoxia, hypoxia or hyperoxia; radial arterial samples were analysed with an IL 682 co-oximeter and three device-specific paired bias/precision estimates are reported.
PUBMED:0080	Hansen, 1987	Validity of ear oximetry in clinical exercise testing.	Included	Not applicable	Fourteen clinical exercise-test patients had paired ear oximetry and arterial IL 282 four-wavelength co-oximetry. Exercise time-course results are graphical rather than a tabulated global agreement estimate.
PUBMED:0084	Norton, 1992	Accuracy of pulse oximetry during exercise stress testing.	Excluded	E12_PAIRED_METHOD_DATA_NOT_EXTRACTABLE	An OSM3 hemoximeter was used only in five participants; the paper explicitly states that further statistical analysis of direct hemoximeter saturation was not computed because of the small number, leaving no extractable paired SpO2-direct SaO2 comparison.
PUBMED:0115	Benoit, 1997	Accuracy of pulse oximetry during intense exercise under severe hypoxic conditions.	Included	Not applicable	Ten adult men completed intense normoxic and hypoxic cycle exercise with simultaneous radial arterial OSM3 co-oximetry. The reported agreement estimates use post-hoc corrected SpO2 and are therefore retained as secondary/sensitivity evidence.
PUBMED:0152	McGovern, 1996	Comparison of oxygen saturation by pulse oximetry and co-oximetry during exercise testing in patients with COPD.	Included	Not applicable	Eight adults with severe COPD completed progressive cycling; radial arterial samples at each workload were analysed using an IL 482 co-oximeter and 49 paired exercise observations yielded bias and precision.
PUBMED:0183	Carone, 1997	Comparison of invasive and noninvasive saturation monitoring in prescribing oxygen during exercise in COPD patients.	Included	Not applicable	Twenty adults with COPD on long-term oxygen therapy completed treadmill walking; radial arterial samples at rest and during walking were analysed using an IL 282 co-oximeter and Bland-Altman bias/limits are reported.
PUBMED:0197	Escourrou, 1990	Reliability of pulse oximetry during exercise in pulmonary patients.	Excluded	E08_ADULT_DATA_NOT_SEPARABLE	The otherwise relevant study reports 101 patients aged 17-81 years and does not provide a separable adult-only dataset. Under the frozen eligibility criterion of age 18 years or older it cannot enter the adult primary synthesis.
PUBMED:0227	Martin, 1992	Validity of pulse oximetry during exercise in elite endurance athletes.	Included	Not applicable	Eleven adult male cyclists completed high-intensity and incremental cycle tests; radial arterial samples were analysed by IL 282 co-oximetry and paired exercise bias/SD are reported for bilateral-ear and finger probes.
PUBMED:0258	Barthélémy, 1990	Accuracy of pulse oximetry during moderate exercise: a comparative study.	Included	Not applicable	Fifty-seven adults completed moderate cycle exercise with radial arterial samples analysed on an OSM3 co-oximeter. Eight finger pulse-oximeter comparisons have extractable signed mean error and SD.

Record ID	Author-year	Title	Final decision	Primary exclusion reason	Decisive evidence note
PUBMED:0292	Breuer, 1990	Comparative analysis of arterial oxygen saturations during exercise by pulse oximetry, photometric measurements, and calculation procedures.	Excluded	E05_NO_DIRECT_ARTERIAL_COOXIMETRY_REFERENCE	Reference samples were arterialized earlobe blood rather than direct arterial blood. The OSM2 measurement therefore fails the direct arterial reference-standard requirement.
PUBMED:1298	Elliott, 2015	AltitudeOmics: impaired pulmonary gas exchange efficiency and blunted ventilatory acclimatization in humans with patent foramen ovale after 16 days at 5260 m.	Excluded	E12_PAIRED_METHOD_DATA_NOT_EXTRACTABLE	The report measured arterial SaO2 by OSM3 co-oximetry during exercise but assessed finger SpO2 only at acute-mountain-sickness assessment. It reports no concurrent exercise SpO2-SaO2 pairs or extractable agreement outcome.
PUBMED:1631	Chan, 1997	Restraint position and positional asphyxia.	Excluded	E12_PAIRED_METHOD_DATA_NOT_EXTRACTABLE	Fifteen healthy men aged 18-40 years exercised with a radial arterial line and co-oximetry, but the reported result is limited to significance statements and a pulse-oximeter-only trace. It supplies no extractable paired SpO2-SaO2 agreement estimate, limits of agreement, table, or individual data.
PUBMED:0468	Wijshoff, 2013	Improving pulse oximetry accuracy by removing motion artifacts from photoplethysmograms using relative sensor motion: a preliminary study.	Excluded	E05_NO_DIRECT_ARTERIAL_COOXIMETRY_REFERENCE	This treadmill-motion study compares a proposed forehead pulse-oximetry algorithm with another pulse-oximetry signal/algorithm. It contains no direct arterial blood sampling or arterial co-oximetry reference.
PUBMED:0375	Powers, 1989	Accuracy of pulse oximetry to estimate HbO2 fraction of total Hb during exercise.	Included	Not applicable	Ten healthy adult men performed incremental cycle exercise; simultaneous radial-arterial samples were analysed by a four-wavelength IL 282 co-oximeter. Table 1 reports device-specific exercise mean differences and SDs for three pulse-oximeter configurations.
PUBMED:0370	Ries, 1985	Accuracy of two ear oximeters at rest and during exercise in pulmonary patients.	Included	Not applicable	Prospective pulmonary-laboratory study of 116 adults across three phases. Exercise ear-oximeter readings were simultaneous with radial arterial samples analysed by IL 282 co-oximetry; exercise-specific group means and regression SEE values are extractable.
PUBMED:0226	Carlin, 1994	The effects of exercise testing on the prescription of oxygen therapy.	Excluded	E12_PAIRED_METHOD_DATA_NOT_EXTRACTABLE	The adult COPD exercise study used radial arterial sampling and IL 282 co-oximetry, but reports correlations and clinical threshold classification rather than a usable paired bias, paired SD, limits of agreement, tabulated pairs, or digitizable agreement data.
PUBMED:0223	Rice, 1999	Exercise-induced hypoxaemia in highly trained cyclists at 40% peak oxygen uptake.	Included	Not applicable	Fifteen adult male cyclists had simultaneous radial-arterial sampling and ear oximetry during incremental cycling. The report identifies SaO2 measurement on an ABL 520 and presents a graphical exercise comparison; eligibility is confirmed, while the graphical result remains non-poolable.
PUBMED:0205	Orenstein, 1993	Accuracy of three pulse oximeters during exercise and hypoxemia in patients with cystic fibrosis.	Excluded	E08_ADULT_DATA_NOT_SEPARABLE	The report has direct arterial OSM3 co-oximetry and concurrent exercise comparisons, but its nine-person cystic-fibrosis cohort includes three participants younger than 18 years, aged 13, 14, and 15 years, plus six adults. Adult-only data are not separable.
PUBMED:0122	Lundgren, 2001	Indirect and direct gas exchange at maximum exercise in beryllium sensitization and disease.	Included	Not applicable	Clinical cycle-cardiopulmonary exercise testing data from 168 adults used a radial arterial line and direct co-oximetry. Maximum-exercise signed underestimation with SD is reported for the beryllium-sensitization group, with additional chronic-beryllium-disease group means.

Record ID	Author-year	Title	Final decision	Primary exclusion reason	Decisive evidence note
PUBMED:0118	Mollard, 2010	Validity of arterialized earlobe blood gases at rest and exercise in normoxia and hypoxia.	Excluded	E05_NO_DIRECT_ARTERIAL_COOXIMETRY_REFERENCE_CONFIRMED	Methods 2.3.2 says multi-wavelength co-oximetry, but Table 1 footnote says SO2 was calculated from PO2 and pH. The reference saturation used in the SpO2 comparison cannot be confirmed as directly measured. The authors confirmed exclusion under the reference-method criterion on 30 September 2026.
PUBMED:0091	Campedelli, 2024	Non-arterial line cardiac output calculation misclassifies exercise pulmonary hypertension and increases risk of data loss particularly in black, scleroderma and Raynaud's patients during invasive exercise testing.	Included	Not applicable	Retrospective adult invasive cardiopulmonary exercise testing cohort. Radial arterial blood gases were measured by ABL 90 Flex Plus co-oximetry and SpO2 was recorded at the exact blood-draw time; peak-exercise paired mean differences are reported.
PUBMED:0073	Roberts, 1999	Erythropoietin concentration and arterial haemoglobin saturation with supramaximal exercise.	Included	Not applicable	Eight adult athletes underwent radial arterial sampling during supramaximal cycling, with direct four-wavelength IL 482 co-oximetry and synchronized forehead pulse oximetry. Bland-Altman agreement and condition-specific differences are reported; repeated samples are retained as non-independent.
PUBMED:0213	Stege, 2009	Accuracy of transcutaneous carbon dioxide tension measurements during cardiopulmonary exercise testing.	Excluded	E14_REFERENCE_STANDARD_NOT_CONFIRMABLE_ON_FULLTEXT	Full text confirms adults, incremental cycle exercise, radial-artery samples, and repeated paired observations. The laboratory analyzer is identified only as Roche Omni S blood-gas analysis, without confirmation of a co-oximetry panel or direct hemoglobin-fraction measurement. The report was excluded under the protocol-defined reference-standard criterion.
PMID:42664382	Hansen et al., 2026	Pulse Oximetry and Arterial Blood Gas Agreement in an Outpatient Black Lung Clinic: Implications for Clinical Assessment and Decision Making.	Excluded	E14_REFERENCE_STANDARD_NOT_CONFIRMABLE_ON_FULLTEXT	Full report describes ABG-derived SaO2 but gives no co-oximeter model, co-oximetry panel, or direct hemoglobin-fraction evidence; postexercise pairing is reported only as within minutes.

Record ID	Author-year	Title	Final decision	Primary exclusion reason	Decisive evidence note
PUBMED:0404	Chailleux et al., 1989	[Reliability of transcutaneous PO2 measurements and ear oximetry at different levels of exercise].	EXCLUDE	E04_NO_PULSE_OXIMETRY_SPO2_INDEX	Both authors confirmed subsequent full-text retrieval and exclusion because the index did not meet the pulse-oximetry SpO2 criterion (30 September 2026). Author-confirmed decision; full-text file/page locator not supplied to the assistant.

e-Table S2, Part B. Reports sought but not retrieved

Eligibility remains unknown for these eight reports. Unretrieved reports are not treated as full-text exclusions.

Record ID	Complete citation	Priority	Routes checked	Routes not completed	Reason unavailable	Eligibility status	Plausible impact	Reopening rule
PUBMED: 0426	Wood RJ, Gore CJ, Hahn AG, et al. Accuracy of two pulse oximeters during maximal cycling exercise. Australian Journal of Science and Medicine in Sport. 1997;29(2):47-50.	A	PubMed metadata and full-text-link check; no PMID-linked DOI or PMCID; University of Adelaide repository metadata/abstract checked but no downloadable bitstream; institutional/Ovid lead resolved to a secondary abstracts page rather than the article	Interlibrary-loan request not submitted; authors not contacted	No lawful full-text file was identified before the retrieval phase was closed by author decision	Unknown; not treated as excluded	High: the abstract describes adult maximal cycling with arterial co-oximetry and could add a direct peak/maximal exercise study, alter the G1 compatibility assessment, and change the included-study count	Retrieve lawfully, reassess eligibility in duplicate/consensus, and rerun extraction, appraisal, synthesis, PRISMA, manuscript, and gates
PUBMED: 0469	Stanghelle JK, Christensen CC, Haanaes OC. Invasive or transcutaneous measurements of oxygen saturation and blood gases? An evaluation of pulse oximetry and transcutaneous measurement of PO2 and PCO2 during rest and exercise. Tidsskrift for den Norske laegeforening. 1993;113(8):967-970.	A	PubMed metadata and full-text-link check; no PMID-linked DOI or PMCID; journal/archive and legal-access routes checked without locating a file	Interlibrary-loan request not submitted; authors not contacted	No lawful full-text file was identified before the retrieval phase was closed by author decision	Unknown; not treated as excluded	Moderate to high: the title directly concerns invasive versus noninvasive measurement during rest and exercise, but population, co-oximetry confirmation, timing, and paired statistics are unknown	Retrieve lawfully and reopen full downstream adjudication if eligible

Record ID	Complete citation	Priority	Routes checked	Routes not completed	Reason unavailable	Eligibility status	Plausible impact	Reopening rule
PUBMED: 0489	Perez-Padilla JR, Bracamonte-Peraza R, Manrique G. Accuracy of an ear oximeter Biox-III and its sensitivity to carboxyhemoglobin in Mexico City. Archivos del Instituto de Cardiologia de Mexico. 1986;56(4):303-307.	A	PubMed metadata and full-text-link check; no PMID-linked DOI or PMCID; legal open-access and institutional-route check found no file	Interlibrary-loan request not submitted; authors not contacted	No lawful full-text file was identified before the retrieval phase was closed by author decision	Unknown; not treated as excluded	Moderate: the report may include a Biox-III arterial comparison and carboxyhemoglobin effects, but exercise timing and extractable paired agreement are unknown	Retrieve lawfully and reopen full downstream adjudication if eligible
PUBMED: 0188	Jeyasingham K. Some studies in ear oximetry. Ceylon Med J. 1962;7:23-31.	B	PubMed metadata and full-text-link check; no PMID-linked DOI or PMCID; legal open-access route check found no file	Not prioritized for interlibrary loan; authors not contacted	Very old broad report with no exercise-specific eligibility established from indexed metadata	Unknown; not treated as excluded	Low but nonzero: eligibility, exercise timing, direct arterial co-oximetry, and paired agreement cannot be determined from metadata	Retrieve if lawfully supplied and reassess eligibility
PUBMED: 0527	Scebat L. Data given by the study of blood oximetry. Rev Prat. 1972;22(7):1129-1134 passim.	B	PubMed metadata and full-text-link check; no PMID-linked DOI or PMCID; legal open-access route check found no file	Not prioritized for interlibrary loan; authors not contacted	Broad/unclear report type with no exercise-specific eligibility established from indexed metadata	Unknown; not treated as excluded	Low but nonzero: the title does not establish an eligible exercise comparison, but eligibility remains unknown without full text	Retrieve if lawfully supplied and reassess eligibility
PUBMED: 0564	Chechani V, Scott G. Pulse oximetry in evaluation of oxygen needs of patients with chronic obstructive pulmonary disease (COPD). Mo Med. 1991;88(11):757-761.	B	PubMed metadata and full-text-link check; no PMID-linked DOI or PMCID; legal open-access route check found no file	Not prioritized for interlibrary loan; authors not contacted	No lawful full-text file was identified and indexed metadata did not confirm a paired direct co-oximetry comparison	Unknown; not treated as excluded	Moderate: a COPD exercise/titration context is plausible, but paired direct co-oximetry and the eligible time window are unconfirmed	Retrieve if lawfully supplied and reassess eligibility
PUBMED: 0606	Genger K, Forche G, Zenker G. Ambulatory pulse oximetry in patients with stress hypertension. Wien Med Wochenschr Suppl. 1989;104:suppl 46.	B	PubMed metadata and full-text-link check; no PMID-linked DOI or PMCID; legal open-access route check found no file	Not prioritized for interlibrary loan; authors not contacted	Exercise and direct arterial reference eligibility were not established from indexed metadata	Unknown; not treated as excluded	Low but nonzero: the report may involve exertion, but direct co-oximetry and paired agreement are unconfirmed	Retrieve if lawfully supplied and reassess eligibility
PUBMED: 0795	Scoggin C, Nett L, Petty TL. Clinical evaluation of a new ear oximeter. Heart Lung. 1977;6(1):121-126.	B	PubMed metadata and full-text-link check; no PMID-linked DOI or PMCID; legal open-access route check found no file	Not prioritized for interlibrary loan; authors not contacted	Broad clinical-evaluation report with no exercise/direct-reference eligibility established from indexed metadata	Unknown; not treated as excluded	Low to moderate: an arterial validation may be present, but exercise timing and an eligible paired co-oximetry endpoint are unknown	Retrieve if lawfully supplied and reassess eligibility

e-Table S3. Included-study extraction

Study-level extraction after the author-confirmed Mollard exclusion; 16 studies are retained.

Study	Citation	Design and population	Sample characteristics	Exercise protocol and time point	Index device and site	Arterial reference and sampling	Primary agreement summary	Additional extractable results	Data-quality notes
ASCHA_2018	Ascha M, Bhattacharyya A, Ramos JA, Tonelli AR. Pulse Oximetry and Arterial Oxygen Saturation during Cardiopulmonary Exercise Testing. Med Sci Sports Exerc. 2018;50(10):1992-1997. doi:10.1249/MSS.0000000000001658.	Retrospective clinical cardiopulmonary exercise testing cohort; 751 adults referred for cardiopulmonary exercise testing at Cleveland Clinic Respiratory Institute (January 2010-January 2015); predominantly unexplained dyspnoea/pre-operative assessment.	751 enrolled; 631 exercise pairs; 54.1 ± 16.1 years; 355 male (47%); 396 female (53%)	Electrically braked incremental cycle cardiopulmonary exercise testing; protocol tailored for approximately 8-12 minutes; peak sampling in the final 1-2 minutes before stopping.; Rest and peak exercise; SpO2 recorded at ABG acquisition.	Nonin Avant 4000 pulse oximeter; two forehead probes, waveform-quality check and heart-rate synchronization.; Forehead	ABL800 FLEX Blood Gas Analyzer (Radiometer) using co-oximetry to determine SaO2 from arterial blood gas samples.; Individual radial-artery punctures while seated on the bicycle; no indwelling arterial catheter.	Sign-harmonized mean paired difference 2.6 percentage points; paired SD 2.75; limits -2.9 to 8.1	At peak exercise, 631/751 had both ABG and SpO2; 78/631 (12.4%) had SpO2 ≥5 percentage points above SaO2. Carboxyhaemoglobin was independently associated with larger positive discrepancy.	Numeric direction verified from reported SpO2−SaO2 values and Bland-Altman results. The concluding prose contains potentially ambiguous/discordant wording, so analysis must retain the explicit numeric direction.
SWIGRIS_2009	Swigris JJ, Zhou X, Wamboldt FS, et al. Exercise peripheral oxygen saturation (SpO2) accurately reflects arterial oxygen saturation (SaO2) and predicts mortality in systemic sclerosis. Thorax. 2009;64(7):626-630. doi:10.1136/thx.2008.111393.	Retrospective analysis of prospectively collected cardiopulmonary exercise testing data; 83 adults with systemic-sclerosis-related interstitial lung disease at a Denver ILD centre.	83 enrolled; Not separately reported; results presented for the cohort at maximal exercise. exercise pairs; 49.5 ± 11.5 years; 17 male (20%); 66 female (80%)	Maximal cycle cardiopulmonary exercise testing (Vmax 29): 3 minutes at 60 rpm, then work incremented each minute; target 6-12 minutes.; Baseline and maximum exercise agreement reported.	Peripheral pulse oximeter; index-finger probe opposite radial line, earlobe if no adequate signal; >95% used finger.; Finger (primary); earlobe backup	Arterial blood analysed with a co-oximeter plus blood-gas analyser to measure SaO2.; Radial arterial line; samples at baseline and every minute during exercise.	Sign-harmonized mean paired difference 1.5 percentage points; paired SD 4.0	At maximum exercise, the signed mean (SD) difference was 1.5 (4.0) percentage points; absolute mean (SD) difference 2.98 (2.98). Four subjects had SpO2 >88% but SaO2 ≤88%.	Use the signed 1.5 (4.0) value for bias/precision. Devices changed over the three-decade data-collection period; setting was Denver altitude; retrospective study.
ZEBALLOS_1991	Zeballos RJ, Weisman IM. Reliability of noninvasive oximetry in black subjects during exercise and hypoxia. Am Rev Respir Dis. 1991;144(6):1240-1244. doi:10.1164/ajrccm/144.6.1240.	Prospective repeated-measures method-comparison study; 33 healthy young Black male Army recruits tested under simulated sea level, 2300 m or 4000 m conditions.	33 enrolled; HP 279 and Biox IIA 242 paired observations across rest; exercise; and recovery; condition-specific counts not reported. exercise pairs; 19.0 ± 1.7 years; 33 male	Electronically braked cycle ergometry; 5-minute steady-state stages; normoxia or hypoxic gas mixtures; samples at rest; final minute of every stage; and 2-minute recovery.; Rest; multistage exercise; and 2-minute recovery. Published pooled estimates combine all three periods.	Hewlett-Packard 47201A and Biox IIA ear oximeters.; Earlobes	IL 282 spectrophotometric co-oximeter; functional SaO2 derived from arterial O2Hb/available Hb.; 20-gauge radial arterial catheter; concurrent sample/ear-oximetry readings.	Qualitative or secondary evidence	Six device-by-altitude estimates are in outcome_level_extraction_v0_1_20260830.csv. Pooled condition summaries include both exercise and non-exercise periods.	Repeated measures per participant and mixed rest/exercise/recovery summaries. Do not treat 279 or 242 observations as independent people.
YAMAYA_2002	Yamaya Y, Bogaard HJ, Wagner PD, Niizeki K, Hopkins SR. Validity of pulse oximetry during maximal exercise in normoxia, hypoxia, and hyperoxia. J Appl	Prospective repeated-measures device-comparison study; 9 healthy adults; 6 trained athletes; and 4 adults with chronic disease (COPD or chronic heart failure).	19 enrolled; N-395/RS-10 161; N-395/D-25 157; Ivy 2000 160 paired observations. exercise pairs; Group mean ages 33.5; 28.8; and 56.7 years; Sex	Incremental maximal cycle testing in normoxia/hypoxia; patient group also hyperoxia.; Authors' aggregate normoxia/hypoxia data contain repeated	Nellcor N-395/RS-10; Nellcor N-395/D-25; and Ivy 2000 with LNOP-Adt sensor.; Forehead for RS-10; digit for D-25 and Ivy	IL 682 co-oximeter with arterial SaO2 calculated from O2Hb; COHb; and MetHb fractions.; Radial arterial cannula; duplicate samples at rest and	Qualitative or secondary evidence	Three device-level bias/SD estimates are in outcome_level_extraction_v0_1_20260830.csv. Adequate pulse-rate-signal subset is separately reported	Repeated observations and mixed participant groups. Main device estimates use all available normoxia/hypoxia data and should be

Study	Citation	Design and population	Sample characteristics	Exercise protocol and time point	Index device and site	Arterial reference and sampling	Primary agreement summary	Additional extractable results	Data-quality notes
	Physiol (1985). 2002;92(1):162-168. doi:10.1152/japplphysiol.00409.2001.		distribution reported by group in Table 1	observations across the protocol.		during exercise stages.		but not selected as the primary result.	modelled only with within-study clustering or synthesized narratively.
HANSEN_1987	Hansen JE, Casaburi R. Validity of ear oximetry in clinical exercise testing. Chest. 1987;91(3):333-337. doi:10.1378/chest.91.3.333.	Prospective clinical method-comparison study; 14 consecutive adults with cardiopulmonary disease undergoing clinical exercise testing.	14 enrolled; Not reported; paired serial observations shown graphically. exercise pairs; Individual ages reported in a patient table; aggregate value not extracted.; Clinical cardiopulmonary population; sex distribution not extracted	Incremental cycle ergometry with unloaded phase and progressive work increments to symptom limitation.; Rest; unloaded exercise; late exercise; maximal exercise; and 2-minute recovery.	Biox IIA ear oximeter.; Earlobe	IL 282 four-wavelength co-oximeter for arterial oxyhemoglobin saturation.; Brachial arterial catheter; serial sampling approximately every 1-3 minutes.	Qualitative or secondary evidence	Figure 1 reports time-course group means ±SE and identifies clinically important falsely low/high readings. No tabulated overall bias or paired SD is available.	Eligible study retained for qualitative synthesis. Do not digitize graph values or create a global estimate without raw paired data.
BENOIT_1997	Benoit H, Costes F, Feasson L, Lacour JR, Roche F, Denis C, Geyssant A, Barthélémy JC. Accuracy of pulse oximetry during intense exercise under severe hypoxic conditions. Eur J Appl Physiol Occup Physiol. 1997;76(3):260-263. doi:10.1007/s004210050245.	Prospective repeated-measures method-comparison study; 10 healthy nonsmoking adult men.	10 enrolled; 133 paired observations in reported SaO2 strata: 74 above 75% and 59 at or below 75%. exercise pairs; 24.2 ± 2.8 years; 10 male	Two 30-minute constant-load cycle tests at 80% VO2max: normoxia and hypoxia with FIO2 0.104.; Reported stratum estimates are derived from the study's measurement set; exercise protocol includes repeated observations and may include resting samples.	Datex Satlite pulse oximeter with a post-hoc correction equation applied to SpO2 before comparison.; Earlobe	OSM3 co-oximeter integrated with an ABL520 analyser; functional arterial SaO2.; Radial arterial catheter; 4-6 samples at rest and during exercise; SpO2 recorded at blood sampling.	Qualitative or secondary evidence	Corrected-index high and low SaO2 stratum estimates are in outcome_level_extraction_v0_1_20260830.csv.	The published agreement analysis is for a post-hoc corrected SpO2 rather than raw device output. Retain for sensitivity/technology-history synthesis; exclude from raw-SpO2 primary pooling.
MCGOVERN_1996	McGovern JP, Sasse SA, Stansbury DW, Causing LA, Light RW. Comparison of oxygen saturation by pulse oximetry and co-oximetry during exercise testing in patients with COPD. Chest. 1996;109(5):1151-1155. doi:10.1378/chest.109.5.1151.	Prospective repeated-measures method-comparison study; 8 white men with severe COPD.	8 enrolled; 49 exercise pairs; 63.2 ± 9.6 years; 8 male	Progressive symptom-limited cycle ergometry; 5-15 W workload increments each minute.; All exercise data points; primary result is uncorrected for COHb.	Ohmeda 3700 pulse oximeter with waveform and signal-quality review.; Index finger opposite arterial line	IL 482 co-oximeter.; Radial arterial catheter; SpO2 recorded with arterial samples during the final 15 seconds of each stage.	Sign-harmonized mean paired difference -1.7 percentage points; paired SD 2.9	Source reports SaO2 − SpO2 = +1.7 (SD 2.90) for 49 exercise pairs; standardized here to SpO2 − SaO2 = -1.7. COHb-corrected sensitivity result is separately retained.	Repeated observations in only 8 participants; primary estimate is uncorrected device output. Do not treat 49 observations as independent people.
CARONE	Carone M, Patessio A, Appendini L, Purro A, Czernicka E,	Prospective repeated-test Bland-Altman method-comparison	20 enrolled; Not reported for the all-timepoint agreement	Repeated 6-minute treadmill walking tests with	Biox 3760 ear pulse oximeter.; Earlobe	IL 282 co-oximeter.; Radial arterial sampling at rest and	Sign-harmonized mean paired	All-timepoint Bland-Altman mean difference is -0.67	Main agreement set combines baseline and repeated walking

Study	Citation	Design and population	Sample characteristics	Exercise protocol and time point	Index device and site	Arterial reference and sampling	Primary agreement summary	Additional extractable results	Data-quality notes
_1997	Zanaboni S, Donner CF. Comparison of invasive and noninvasive saturation monitoring in prescribing oxygen during exercise in COPD patients. Eur Respir J. 1997;10(2):446-451. doi:10.1183/09031936.97.10020446.	study; 20 adults with COPD receiving long-term oxygen therapy and exercise desaturation.	set; 47 sixth-minute pairs were used only for the clinical cut-off analysis. exercise pairs; 62 ± 8 years; Sex distribution not extracted	progressively titrated supplemental oxygen.; Baseline and 2nd/4th/6th-minute walking values; repeated tests and oxygen titration.		every 2 minutes during treadmill tests.	difference -0.67 percentage points; paired SD 3.17; limits -6.88 to 5.55	(SD 3.17) percentage points; reported limits of agreement -6.88 to +5.55.	timepoints; number of pairs not explicitly stated. The 47 sixth-minute points must not be substituted for the all-timepoint agreement n.
MARTIN_1992	Martin D, Powers S, Cicale M, Collop N, Huang D, Criswell D. Validity of pulse oximetry during exercise in elite endurance athletes. J Appl Physiol (1985). 1992;72(2):455-458. doi:10.1152/jappl.1992.72.2.455.	Prospective repeated-measures device/site comparison; 11 Caucasian nonsmoking elite male cyclists.	11 enrolled; 232 paired exercise observations per probe. exercise pairs; 24.8 ± 3.4 years; 11 male	High-intensity constant-load cycling at 90-95% VO2max plus incremental tests; three participants also completed hypoxic incremental exercise.; Exercise subset reported separately; primary device/site estimates use 232 exercise observations.	Three Ohmeda 3740 pulse oximeters used simultaneously.; Both earlobes and index finger	IL 282 CO-oximeter measuring arterial oxyhemoglobin fraction of total hemoglobin.; Radial arterial catheter; samples at rest and during the final 30 seconds of each exercise stage.	Sign-harmonized mean paired difference 0.52 percentage points; paired SD 1.36	Finger probe primary exercise estimate is +0.52 (SD 1.36); bilateral ear-device outcomes are separately retained in outcome_level_extraction_v0_1_20260830.csv.	Repeated observations and three simultaneous devices. Outcomes compare SpO2 to fractional HbO2 rather than functional SaO2; preserve this reference-definition distinction in analysis.
BARTHELEMY_1990	Barthélémy JC, Geyssant A, Riffat J, Antoniadis A, Berruyer J, Lacour JR. Accuracy of pulse oximetry during moderate exercise: a comparative study. Scand J Clin Lab Invest. 1990;50(5):533-539. doi:10.1080/00365519009089168.	Prospective repeated-measures multi-device comparison; 57 adults undergoing moderate cycle exercise in a clinical exercise laboratory.	57 enrolled; Device-specific repeated paired observations: 33 to 153 per device. exercise pairs; 22-78 years; 54 male; 3 female	Moderate cycling; radial arterial samples were taken at 1-minute intervals during exercise.; Exercise-only sampling; table reports 75-100% SfO2 aggregate per device.	Eight finger pulse oximeters: Datex; Physiocontrol; Radiometer Oxi; Nellcor; Ohmeda; Novametrix; Kontron; and PPG-Hellige.; Finger	OSM3 co-oximeter measuring functional arterial saturation (SfO2).; Radial arterial catheter; simultaneous sampling and SpO2 during exercise.	Qualitative or secondary evidence	Eight device-specific mean-error and SD values are in outcome_level_extraction_v0_1_20260830.csv.	Multiple repeated observations per subject and multiple devices; preserve device-stratified outcomes and do not treat each measurement as an independent participant.
POWERS_1989	Powers SK, Dodd S, Freeman J, Ayers GD, Samson H, McKnight T. Accuracy of pulse oximetry to estimate HbO2 fraction of total Hb during exercise. J Appl Physiol (1985). 1989;67(1):300-304. doi:10.1152/jappl.1989.67.1.300.	Prospective repeated-measures multi-device method-comparison study; 10 healthy adult men; six exercised in normoxia and four in hypoxia; four participants were smokers.	10 enrolled; 70 repeated exercise samples per configuration; 10 participants at highest work rate per configuration. exercise pairs; 21-36 years; 10 male	Incremental cycle ergometry from 30 W with 15 W per minute increments to volitional fatigue; four participants exercised at PiO2 107 Torr.; Rest; lowest work rate; and highest work rate. Highest-work device estimates retained separately.	Ohmeda 3700 finger; Ohmeda 3700 ear; Biox IIa ear.; Finger and both ears	Four-wavelength IL 282 CO-oximeter directly measuring fractional arterial HbO2; functional SaO2 additionally calculated from co-oximeter fractions.; Radial arterial catheter; samples in the last 30 seconds of each work rate,	Qualitative or secondary evidence	Three highest-work device outcomes are retained in outcome_level_extraction_v0_1_20260830.csv; source values are reported as HbO2 minus SpO2 and have been direction-standardized.	Repeated observations and three simultaneous device/site configurations. Fractional HbO2 and calculated functional SaO2 must not be silently conflated.

Study	Citation	Design and population	Sample characteristics	Exercise protocol and time point	Index device and site	Arterial reference and sampling	Primary agreement summary	Additional extractable results	Data-quality notes
						synchronized to each device reading.			
RIES_1985	Ries AL, Farrow JT, Clausen JL. Accuracy of two ear oximeters at rest and during exercise in pulmonary patients. Am Rev Respir Dis. 1985;132(3):685-689. doi:10.1164/arrd.1985.132.3.685.	Prospective multi-phase repeated-measures method-comparison study; 116 adult pulmonary-laboratory patients; Phase 1 Hewlett-Packard, Phase 2 Biox, and Phase 3 concurrent devices.	116 total; exercise sample counts vary by phase enrolled; Exercise pairs: HP all phases 315; Biox phase 2 125; phase 3 54 per device. exercise pairs; Not reported in extractable form; Pulmonary patient cohort; sex distribution not extracted.	Standard clinical treadmill or cycle exercise testing at modest levels; steady-state or maximal symptom-limited protocols.; Exercise-only rows are available for each reported device/phase.	Hewlett-Packard 47201A and Biox IIA ear oximeters.; Earlobe	Four-wavelength IL 282 co-oximeter directly measuring arterial SaO2; calculated saturation was also described but not used as the reference.; Indwelling radial arterial catheter; simultaneous oximeter recording and arterial sampling within 5 seconds.	Qualitative or secondary evidence	Exercise group-mean differences and regression SEE values for four device/phase rows are retained in outcome_level_extraction_v0_1_20260830.csv.	Published precision is regression SEE, not the SD of paired differences. HP all-phase data overlap the Phase 3 HP subset; do not pool any reported rows as independent.
RICE_1999	Rice AJ, Scroop GC, Gore CJ, Thornton AT, Chapman MAJ, Greville HW, Holmes MD, Scicchitano R. Exercise-induced hypoxaemia in highly trained cyclists at 40% peak oxygen uptake. Eur J Appl Physiol. 1999;79:353-359. doi:10.1007/s004210050520.	Prospective repeated-measures exercise physiology study; 15 competitive adult male cyclists.	15 enrolled; Repeated arterial samples at rest and each 2-minute incremental exercise stage; count not reported. exercise pairs; Not reported in extractable form; 15 male	Cycle ergometry from 150 W with 25 W increments every 2 minutes to exhaustion.; Rest and progressive exercise; Figure 4 displays the comparison across work rates.	Criticare US-504 two-wavelength ear oximetry.; Earlobe	ABL 520 analyzer for arterial SaO2; method report treats SaO2 as directly measured and this was externally cross-checked as a direct oxygen-saturation measure.; Left radial arterial catheter; samples during the final 30 seconds of each stage and simultaneous ear oximetry.	Qualitative or secondary evidence	Figure 4 shows ear SpO2 significantly higher than temperature-adjusted arterial SaO2 at all exercise intensities, but provides no table or extractable pairwise bias/precision estimate.	Graphical-only comparison; no digitization planned under the frozen protocol. Repeated values and direct-reference-method detail should be retained for qualitative synthesis.
LUNDGREN_2001	Lundgren RA, Maier LA, Rose CS, Balkissoon RC, Newman LS. Indirect and direct gas exchange at maximum exercise in beryllium sensitization and disease. Chest. 2001;120(5):1702-1708. doi:10.1378/chest.120.5.1702.	Retrospective clinical cardiopulmonary exercise testing cohort; 168 adults evaluated for possible chronic beryllium disease: beryllium sensitization (beryllium sensitization) n=67 and chronic beryllium disease (chronic beryllium disease) n=101.	168 enrolled; One maximum-exercise measurement per participant; numerical bias/SD available for beryllium sensitization n=67. exercise pairs; beryllium sensitization median 53 years (range 27-80); chronic beryllium disease median 51 years (range 31-78).; beryllium sensitization 58 male; chronic beryllium disease 79 male.	Cycle ergometry: 3 minutes unloaded at 60 rpm then 1-minute increments to maximum capacity within 6-12 minutes.; Maximum exercise; beryllium sensitization signed underestimation and chronic beryllium disease group means reported.	Ohmeda 3740 Bioximeter (1985-1993) and SensorMedics Sat-Trak (1993-1998).; Earlobe	Radiometer ABL2 plus IL 282 co-oximeter (1985-1990), then IL 1620 blood-gas analyzer plus IL 682 co-oximeter (1990-1998); co-oximeter measured SaO2.; Indwelling radial arterial line; baseline and every minute during exercise.	Sign-harmonized mean paired difference -0.88 percentage points; paired SD 4.6	beryllium sensitization maximum-exercise SpO2 underestimation of SaO2: 0.88 (SD 4.6) percentage points; chronic beryllium disease group means yield SpO2 minus SaO2 of +0.3 with no paired-difference SD.	Cohort spans two oximeter generations and co-oximeter platforms. Use the beryllium sensitization n=67 signed result as the extractable primary outcome; chronic beryllium disease mean subtraction is descriptive only.

Study	Citation	Design and population	Sample characteristics	Exercise protocol and time point	Index device and site	Arterial reference and sampling	Primary agreement summary	Additional extractable results	Data-quality notes
CAMPEDELLI_2024	Campedelli L, Nouraie SM, Risbano MG. Non-arterial line cardiac output calculation misclassifies exercise pulmonary hypertension and increases risk of data loss particularly in black, scleroderma and Raynaud's patients during invasive exercise testing. Eur Respir J. 2024;64:2302232. doi:10.1183/13993003.02232-2023.	Retrospective invasive cardiopulmonary exercise testing cohort; 296 consecutive adults referred for exercise intolerance at the University of Pittsburgh; 268 had complete peak data and 265 were in the outlier-excluded sensitivity analysis.	296 enrolled; 268 peak-exercise paired observations; 265 in sensitivity analysis. exercise pairs; Not reported in extractable form; Not reported in extractable form	Upright cycle ramp iCPET at 5-20 W/min; radial arterial samples at rest, freewheel, every 2 minutes, and peak.; Peak exercise primary and outlier-excluded sensitivity analyses; all-stage repeated measures analysed separately.	Stryker Sustainability Solutions pulse oximeter.; Index finger primarily; earlobe or forehead if signal inadequate	ABL 90 Flex Plus (Radiometer) co-oximetry measuring arterial SaO2, oxyhaemoglobin, carboxyhaemoglobin, and methaemoglobin.; Radial arterial catheter; displayed SpO2 recorded at the exact blood-gas draw time.	Sign-harmonized mean paired difference -0.44 percentage points; paired SD 7.19	Peak source mean SaO2 minus SpO2 was +0.44 (95% CI -0.42 to +1.31) in n=268, corresponding to an approximately 7.19-point paired-difference SD reconstructed from the reported mean CI and pair count. The n=265 sensitivity analysis reported +0.11 (95% CI -0.67 to +0.90), corresponding to an approximately 6.49-point paired-difference SD; all-stage ICC was 0.25 then 0.24.	The authors explicitly describe the interval as the 95% CI of the paired mean difference but call it a limit of agreement. We reconstructed the paired-difference SD from the CI width and n using the two-sided Student t critical value; the CI was not treated as a Bland-Altman limit of agreement. Site changes after signal failure and missing SpO2 data can induce selection and heterogeneity.
ROBERTS_1999	Roberts D, Smith DJ. Erythropoietin concentration and arterial haemoglobin saturation with supramaximal exercise. J Sports Sci. 1999;17(6):485-493. doi:10.1080/026404199365795.	Prospective repeated-measures arterial-validation substudy; 20 athletes completed the parent study; eight volunteers underwent arterial catheterization for the SpO2 validation substudy.	20 enrolled; 65 repeated arterial/pulse-oximeter observations from 8 arterial-study athletes. exercise pairs; Validation participants sampled from athletic groups aged approximately 20-24 years.; Sex not reported in extractable form.	3-minute supramaximal cycling at 109 ± 2.8% maximal oxygen consumption, with serial sampling through recovery.; Rest, supramaximal exercise, and recovery; reported summaries contain overlapping repeated observations.	Nellcor N200 with RS-10 reflective sensor.; Forehead	Four-wavelength IL 482 co-oximeter directly measuring arterial oxyhaemoglobin saturation.; Radial arterial catheter; samples every 30 seconds initially, then every 15 seconds in the last minute and until baseline recovery, synchronized with recorded SpO2.	Sign-harmonized mean paired difference 0.1 percentage points; limits -3.7 to 3.9	Aggregate 65-observation Bland-Altman result standardizes to +0.1 percentage points with reversed limits -3.7 to +3.9. Table 5 preserves condition-specific overlapping summaries.	The reported SE/CI and limits do not support a clean paired-difference SD derivation. All rows are repeated samples from eight participants; condition thresholds overlap and cannot be entered as independent effects.

e-Table S4. Device- and condition-level agreement outcomes

The 46 rows are correlated results and must not be interpreted as 46 independent study effects.

Study / outcome	Synthesis role/status	Device / site	Arterial reference	Exercise time point or condition	N participants / pairs	Standardized bias	Reported precision or interval	Unit of analysis	Notes and source pages
ZEBALLOS_1991 / ZEBALLOS_1991_HP_SEA_LEVEL	PRIMARY_CANDIDATE; NOT_POOLABLE_MIXED_REST_EXERCISE_RECOVERY	Hewlett-Packard 47201A; Earlobe	IL 282 co-oximeter	rest; final minute of three 5-minute cycle stages; 2-minute recovery; simulated sea level	33 / NR	-0.4	95% confidence interval -0.7 to -0.1; SD 1.7	Repeated measures; condition-specific pair count not reported	Published aggregate includes rest; exercise; and recovery. 95% CI of mean bias reported as plus/minus 0.3 percentage points.; pp.1241-1243
ZEBALLOS_1991 / ZEBALLOS_1991_BIOX_SEA_LEVEL	PRIMARY_CANDIDATE; NOT_POOLABLE_MIXED_REST_EXERCISE_RECOVERY	Biox IIA; Earlobe	IL 282 co-oximeter	rest; final minute of three 5-minute cycle stages; 2-minute recovery; simulated sea level	33 / NR	2.1	95% confidence interval 1.8 to 2.4; SD 2.0	Repeated measures; condition-specific pair count not reported	Published aggregate includes rest; exercise; and recovery. 95% CI of mean bias reported as plus/minus 0.3 percentage points.; pp.1241-1243
ZEBALLOS_1991 / ZEBALLOS_1991_HP_2300M	PRIMARY_CANDIDATE; NOT_POOLABLE_MIXED_REST_EXERCISE_RECOVERY	Hewlett-Packard 47201A; Earlobe	IL 282 co-oximeter	rest; final minute of steady-state cycle stages; 2-minute recovery; simulated 2300 m	11 / NR	-0.8	95% confidence interval -1.3 to -0.3; SD 1.7	Repeated measures; condition-specific pair count not reported	Published aggregate includes rest; exercise; and recovery. 95% CI of mean bias reported as plus/minus 0.5 percentage points.; pp.1241-1243
ZEBALLOS_1991 / ZEBALLOS_1991_BIOX_2300M	PRIMARY_CANDIDATE; NOT_POOLABLE_MIXED_REST_EXERCISE_RECOVERY	Biox IIA; Earlobe	IL 282 co-oximeter	rest; final minute of steady-state cycle stages; 2-minute recovery; simulated 2300 m	11 / NR	3.5	95% confidence interval 2.6 to 4.4; SD 2.8	Repeated measures; condition-specific pair count not reported	Published aggregate includes rest; exercise; and recovery. 95% CI of mean bias reported as plus/minus 0.9 percentage points.; pp.1241-1243
ZEBALLOS_1991 / ZEBALLOS_1991_HP_4000M	PRIMARY_CANDIDATE; NOT_POOLABLE_MIXED_REST_EXERCISE_RECOVERY	Hewlett-Packard 47201A; Earlobe	IL 282 co-oximeter	rest; final minute of steady-state cycle stages; 2-minute recovery; simulated 4000 m	22 / NR	-4.8	95% confidence interval -6.4 to -3.2; SD 6.9	Repeated measures; condition-specific pair count not reported	Published aggregate includes rest; exercise; and recovery. 95% CI of mean bias reported as plus/minus 1.6 percentage points.; pp.1241-1243
ZEBALLOS_1991 / ZEBALLOS_1991_BIOX_4000M	PRIMARY_CANDIDATE; NOT_POOLABLE_MIXED_REST_EXERCISE_RECOVERY	Biox IIA; Earlobe	IL 282 co-oximeter	rest; final minute of steady-state cycle stages; 2-minute recovery; simulated 4000 m	22 / NR	9.8	95% confidence interval 8.0 to 11.6; SD 6.9	Repeated measures; condition-specific pair count not reported	Published aggregate includes rest; exercise; and recovery. 95% CI of mean bias reported as plus/minus 1.8 percentage points.; pp.1241-1243
YAMAYA_2002 / YAMAYA_2002_RS10_FOREHEAD	PRIMARY_CANDIDATE; CONDITIONAL_MULTILEVEL_ONLY	Nellcor N-395 with RS-10; Forehead	IL 682 co-oximeter	all normoxia/hypoxia observations across maximal cycle protocol; rest and exercise not separately summarized	19 / 161	0.3	SD 2.5	Repeated measures across subjects and protocols	Authors report bias plus/minus precision SD for all data. Analyses also provide an adequate pulse-rate-signal subset that is not selected as primary.; p.165
YAMAYA_2002 / YAMAYA_200	PRIMARY_CANDIDATE; CONDITIONAL_	Nellcor N-395 with D-25; Digit	IL 682 co-oximeter	all normoxia/hypoxia observations across maximal cycle protocol; rest and	19 / 157	-2.0	SD 7.3	Repeated measures across subjects and protocols	Authors report bias plus/minus precision SD for all data. Analyses also provide an adequate pulse-rate-signal subset

Study / outcome	Synthesis role/status	Device / site	Arterial reference	Exercise time point or condition	N participants / pairs	Standardized bias	Reported precision or interval	Unit of analysis	Notes and source pages
2_D25_FINGER	MULTILEVEL_ONLY			exercise not separately summarized					that is not selected as primary.; p.165
YAMAYA_2002 / YAMAYA_2002_IVY_FINGER	PRIMARY_CANDIDATE; CONDITIONAL_MULTILEVEL_ONLY	Ivy 2000 with LNOP-Adt; Digit	IL 682 co-oximeter	all normoxia/hypoxia observations across maximal cycle protocol; rest and exercise not separately summarized	19 / 160	-2.0	SD 5.2	Repeated measures across subjects and protocols	Authors report bias plus/minus precision SD for all data. Analyses also provide an adequate pulse-rate-signal subset that is not selected as primary.; p.165
HANSEN_1987 / HANSEN_1987_BIOX_TIMECOURSE	ELIGIBLE_NONPOOLABLE; NOT_POOLABLE_GRAPH_ONLY	Biox IIA; Earlobe	IL 282 four-wavelength co-oximeter	rest; unloaded cycling; late exercise; maximal exercise; 2-minute recovery	14 / NR	NR	NR	Repeated paired time-course observations presented only as group means plus/minus SE in a figure	Figure reports three clinically defined groups and no tabulated overall paired bias or SD. No digitized value is entered.; p.335
BENOIT_1997 / BENOIT_1997_CORRECTED_HIGH_SAO2	SECONDARY_ONLY_CORRECTED_INDEX; SECONDARY_ONLY_POSTHOC_CORRECTED_INDEX	Datex Satlite; Earlobe	OSM3 co-oximeter integrated with ABL520	intense constant-load cycling; SaO2 above 75%; measurements from normoxic/hypoxic protocol	10 / 74	2.05	SD 0.87	Repeated observations stratified by reference saturation	Index is post-hoc corrected SpO2 rather than raw device output.; p.261
BENOIT_1997 / BENOIT_1997_CORRECTED_LOW_SAO2	SECONDARY_ONLY_CORRECTED_INDEX; SECONDARY_ONLY_POSTHOC_CORRECTED_INDEX	Datex Satlite; Earlobe	OSM3 co-oximeter integrated with ABL520	intense constant-load cycling; SaO2 at or below 75%; measurements from normoxic/hypoxic protocol	10 / 59	1.80	SD 1.81	Repeated observations stratified by reference saturation	Index is post-hoc corrected SpO2 rather than raw device output.; p.261
MCGOVERN_1996 / MCGOVERN_1996_RAW_SPO2	PRIMARY_CANDIDATE; CONDITIONAL_MULTILEVEL_ONLY	Ohmeda 3700; Index finger	IL 482 co-oximeter	all exercise data points during progressive cycle test	8 / 49	-1.7	SD 2.90	Repeated observations in eight participants	Source defines bias as SaO2 minus SpO2. Standardized here to SpO2 minus SaO2.; p.1153
MCGOVERN_1996 / MCGOVERN_1996_COHB_CORRECTED	SECONDARY_ONLY_CORRECTED_INDEX; SECONDARY_ONLY_POSTHOC_CORRECTED_INDEX	Ohmeda 3700 corrected for COHb; Index finger	IL 482 co-oximeter	all exercise data points during progressive cycle test	8 / 49	-4.1	SD 2.23	Repeated observations in eight participants	Sensitivity analysis after mathematical correction of SpO2 for COHb; not raw device output.; p.1153
CARONE_1997 / CARONE_1997_BIOX_ALL_TIMEPOINTS	PRIMARY_CANDIDATE; CONDITIONAL_MULTILEVEL_ONLY	Biox 3760; Earlobe	IL 282 co-oximeter	baseline plus 2nd; 4th; and 6th minutes of repeated treadmill walking with supplemental oxygen titration	20 / NR	-0.67	95% confidence interval -1.12 to -0.22; SD 3.17; limits -6.88 to 5.55	Repeated measures and repeated tests; main pair count not reported	All-timepoint Bland-Altman estimate. The 47 sixth-minute pairs belong to a separate clinical cut-off analysis and are not used as the agreement n.; p.448

Study / outcome	Synthesis role/status	Device / site	Arterial reference	Exercise time point or condition	N participants / pairs	Standardized bias	Reported precision or interval	Unit of analysis	Notes and source pages
MARTIN_1992 / MARTIN_1992_LEFT_EAR_EXERCISE	PRIMARY_CANDIDATE; CONDITIONAL_MULTILEVEL_ONLY	Ohmeda 3740; Left earlobe	IL 282 co-oximeter fractional HbO2	exercise subset across high-intensity constant-load and incremental cycle tests	11 / 232	0.87	95% confidence interval 0.54 to 1.21; SD 2.60	Repeated observations per cyclist; simultaneous multi-site devices	Reference is fractional HbO2; retain distinction from functional SaO2.; p.456
MARTIN_1992 / MARTIN_1992_RIGHT_EAR_EXERCISE	PRIMARY_CANDIDATE; CONDITIONAL_MULTILEVEL_ONLY	Ohmeda 3740; Right earlobe	IL 282 co-oximeter fractional HbO2	exercise subset across high-intensity constant-load and incremental cycle tests	11 / 232	-0.59	95% confidence interval -0.90 to -0.28; SD 2.40	Repeated observations per cyclist; simultaneous multi-site devices	Reference is fractional HbO2; retain distinction from functional SaO2.; p.456
MARTIN_1992 / MARTIN_1992_FINGER_EXERCISE	PRIMARY_CANDIDATE; CONDITIONAL_MULTILEVEL_ONLY	Ohmeda 3740; Index finger	IL 282 co-oximeter fractional HbO2	exercise subset across high-intensity constant-load and incremental cycle tests	11 / 232	0.52	95% confidence interval 0.34 to 0.70; SD 1.36	Repeated observations per cyclist; simultaneous multi-site devices	Reference is fractional HbO2; retain distinction from functional SaO2. High-intensity subset n=75 gives -0.01 plus/minus 1.40.; p.456
BARTHELEMY_1990 / BARTHELEMY_1990_DATEX_75_100	PRIMARY_CANDIDATE; CONDITIONAL_MULTILEVEL_ONLY	Datex Satlite; Index finger	OSM3 co-oximeter functional SfO2	moderate cycle exercise; all observations with SfO2 75-100%	57 / 107	-0.61	SD 1.76	Repeated simultaneous exercise measurements; n is measurements	Table I upper panel.; p.535
BARTHELEMY_1990 / BARTHELEMY_1990_PHYSIOCONTROL_75_100	PRIMARY_CANDIDATE; CONDITIONAL_MULTILEVEL_ONLY	Physiocontrol 1600; Index finger	OSM3 co-oximeter functional SfO2	moderate cycle exercise; all observations with SfO2 75-100%	57 / 70	0.15	SD 1.78	Repeated simultaneous exercise measurements; n is measurements	Table I upper panel.; p.535
BARTHELEMY_1990 / BARTHELEMY_1990_RADIOMETER_75_100	PRIMARY_CANDIDATE; CONDITIONAL_MULTILEVEL_ONLY	Radiometer Oxi; Index finger	OSM3 co-oximeter functional SfO2	moderate cycle exercise; all observations with SfO2 75-100%	57 / 83	-1.54	SD 1.87	Repeated simultaneous exercise measurements; n is measurements	Table I upper panel.; p.535
BARTHELEMY_1990 / BARTHELEMY_1990_NELLCOR_75_100	PRIMARY_CANDIDATE; CONDITIONAL_MULTILEVEL_ONLY	Nellcor N200; Index finger	OSM3 co-oximeter functional SfO2	moderate cycle exercise; all observations with SfO2 75-100%	57 / 153	0.11	SD 3.16	Repeated simultaneous exercise measurements; n is measurements	Table I upper panel.; p.535
BARTHELEMY_1990 / BARTHELEMY_1990_OHMEDA_75_100	PRIMARY_CANDIDATE; CONDITIONAL_MULTILEVEL_ONLY	Ohmeda Biox 3700; Index finger	OSM3 co-oximeter functional SfO2	moderate cycle exercise; all observations with SfO2 75-100%	57 / 151	-2.26	SD 3.25	Repeated simultaneous exercise measurements; n is measurements	Table I upper panel.; p.535
BARTHELEMY_1990 / BARTHELEM	PRIMARY_CANDIDATE; CONDITIONAL_	Novametrix 505; Index finger	OSM3 co-oximeter functional SfO2	moderate cycle exercise; all	57 / 106	0.89	SD 3.55	Repeated simultaneous exercise	Table I upper panel.; p.535

Study / outcome	Synthesis role/status	Device / site	Arterial reference	Exercise time point or condition	N particip ants / pairs	Standa rdized bias	Reported precision or interval	Unit of analysis	Notes and source pages
Y_1990_NOV AMETRIX_75 _100	MULTILEVEL_O NLY			observations with SfO2 75-100%				measurements; n is measurements	
BARTHELEM Y_1990 / BARTHELEM Y_1990_KONT RON_75_100	PRIMARY_CAN DIDATE; CONDITIONAL_ MULTILEVEL_O NLY	Kontron 7840; Index finger	OSM3 co-oximeter functional SfO2	moderate cycle exercise; all observations with SfO2 75-100%	57 / 71	-0.33	SD 3.97	Repeated simultaneous exercise measurements; n is measurements	Table I upper panel.; p.535
BARTHELEM Y_1990 / BARTHELEM Y_1990_PPG_ HELLIGE_75_ 100	PRIMARY_CAN DIDATE; CONDITIONAL_ MULTILEVEL_O NLY	PPG-Hellige 4500; Index finger	OSM3 co-oximeter functional SfO2	moderate cycle exercise; all observations with SfO2 75-100%	57 / 33	-3.26	SD 6.28	Repeated simultaneous exercise measurements; n is measurements	Table I upper panel.; p.535
POWERS_198 9 / POWERS_198 9_OHMEDA37 00_FINGER_H IGHEST	PRIMARY_CAN DIDATE; CONDITIONAL_ MULTILEVEL_O NLY	Ohmeda 3700; Index finger	IL 282 co-oximeter; direct fractional HbO2	highest work rate of incremental cycling	10 / 10	-2.77	SD 2.58	One paired highest-work observation per participant; all devices measured simultaneously	Source direction is direct fractional HbO2 minus SpO2; other device rows are non-independent.; p.301, Table 1
POWERS_198 9 / POWERS_198 9_OHMEDA37 00_EAR_HIGH EST	PRIMARY_CAN DIDATE; CONDITIONAL_ MULTILEVEL_O NLY	Ohmeda 3700; Earlobe	IL 282 co-oximeter; direct fractional HbO2	highest work rate of incremental cycling	10 / 10	-0.87	SD 1.87	One paired highest-work observation per participant; all devices measured simultaneously	Source direction is direct fractional HbO2 minus SpO2; other device rows are non-independent.; p.301, Table 1
POWERS_198 9 / POWERS_198 9_BIOXIIA_E AR_HIGHEST	PRIMARY_CAN DIDATE; CONDITIONAL_ MULTILEVEL_O NLY	Biox IIa; Earlobe	IL 282 co-oximeter; direct fractional HbO2	highest work rate of incremental cycling	10 / 10	-1.67	SD 3.04	One paired highest-work observation per participant; all devices measured simultaneously	Source direction is direct fractional HbO2 minus SpO2; other device rows are non-independent.; p.301, Table 1
RIES_1985 / RIES_1985_HP _ALL_PHASE S_EXERCISE	PRIMARY_CAN DIDATE; NOT_POOLABL E_REPEATED_ MEASURES_NO _PAIR_SD	Hewlett-Packard 47201A; Earlobe	IL 282 co-oximeter; direct arterial SaO2	exercise; HP results pooled across the study's three phases	105 / 315	0.9	NR	Repeated exercise paired samples; HP all-phase cohort includes the Phase 3 HP subset	Reported exercise means: co-ox SaO2 91.3 and HP SpO2 92.2. Regression SEE 1.90 is not stored as paired-difference SD.; p.686, Table 1
RIES_1985 / RIES_1985_BI OX_PHASE2_ EXERCISE	PRIMARY_CAN DIDATE; NOT_POOLABL E_REPEATED_ MEASURES_NO _PAIR_SD	Biox IIA; Earlobe	IL 282 co-oximeter; direct arterial SaO2	exercise; Phase 2, without manufacturer's ear-probe retainer	36 / 125	0.4	NR	Repeated exercise paired samples in a Phase 2 subgroup	Reported exercise means: co-ox SaO2 91.5 and Biox SpO2 91.9. Regression SEE 2.76 is not stored as paired-difference SD.; p.687, Table 2

Study / outcome	Synthesis role/status	Device / site	Arterial reference	Exercise time point or condition	N participants / pairs	Standardized bias	Reported precision or interval	Unit of analysis	Notes and source pages
RIES_1985 / RIES_1985_HP_PHASE3_EXERCISE	SECONDARY_CONDITION; NOT_POOLABLE_REPEATED_MEASURES_NO_PAIR_SD	Hewlett-Packard 47201A; Earlobe	IL 282 co-oximeter; direct arterial SaO2	exercise; Phase 3, simultaneous two-device comparison	23 / 54	0.3	NR	Repeated exercise paired samples; simultaneous with Biox Phase 3 row	Reported exercise means: co-ox SaO2 89.3 and HP SpO2 89.6. Regression SEE 2.40 is not stored as paired-difference SD.; p.688, Table 3
RIES_1985 / RIES_1985_BIOX_PHASE3_EXERCISE	SECONDARY_CONDITION; NOT_POOLABLE_REPEATED_MEASURES_NO_PAIR_SD	Biox IIA; Earlobe	IL 282 co-oximeter; direct arterial SaO2	exercise; Phase 3, simultaneous two-device comparison	23 / 54	0.3	NR	Repeated exercise paired samples; simultaneous with HP Phase 3 row	Reported exercise means: co-ox SaO2 89.3 and Biox SpO2 89.6. Regression SEE 2.24 is not stored as paired-difference SD.; p.688, Table 3
RICE_1999 / RICE_1999_EAR_GRAPHICAL_EXERCISE	QUALITATIVE_ONLY; QUALITATIVE_GRAPHICAL_ONLY	Criticare US-504; Earlobe	ABL 520 arterial SaO2	rest and progressive incremental cycling; Figure 4	15 / NR	NR	NR	Repeated within-participant means shown graphically across workloads	Ear SpO2 was significantly higher than temperature-adjusted arterial SaO2 at every exercise intensity. No graph digitization or numeric effect extraction under the frozen protocol.; Fig. 4
LUNDGREN_2001 / LUNDGREN_2001_BES_MAX	PRIMARY_CANDIDATE; PRIMARY_STUDY_LEVEL	Ohmeda 3740 Bioximeter or SensorMedics Sat-Trak; Earlobe	IL 282 or IL 682 co-oximeter; direct arterial SaO2	maximum exercise; beryllium sensitization (beryllium sensitization)	67 / 67	-0.88	SD 4.6	One maximum-exercise pair per participant	Published mean (SD) underestimation was 0.88 (4.6) percentage points; standardization reverses the sign.; p.1702; Results
LUNDGREN_2001 / LUNDGREN_2001_CBD_MAX	SECONDARY_CONDITION; NOT_POOLABLE_NO_PAIR_SD	Ohmeda 3740 Bioximeter or SensorMedics Sat-Trak; Earlobe	IL 282 or IL 682 co-oximeter; direct arterial SaO2	maximum exercise; chronic beryllium disease (chronic beryllium disease)	101 / 101	0.3	NR	One maximum-exercise pair per participant	chronic beryllium disease peak group means were SpO2 92.3 (SD 3.9) and SaO2 92.0 (SD 4.0); the SD of the paired difference was not reported.; p.1705, Table 3
CAMPEDELLI_2024 / CAMPEDELLI_2024_PEAK_PRIMARY	PRIMARY_CANDIDATE; PRIMARY_STUDY_LEVEL	Stryker Sustainability Solutions pulse oximeter; Index finger primarily; earlobe or forehead if required	ABL 90 Flex Plus co-oximetry; direct arterial SaO2	peak exercise; complete peak data	268 / 268	-0.44	95% confidence interval -1.31 to 0.42; SD 7.19	One peak pair per participant	Source reports SaO2 - SpO2 = +0.44 (95% CI -0.42 to +1.31) and explicitly defines this as a CI of the mean. The sign-harmonized SpO2 - SaO2 CI is -1.31 to +0.42. Paired-difference SD approximately 7.19 was reconstructed from the CI width and n=268 using the two-sided Student t critical value; the source's label 'limit of agreement' was not adopted.; Results; Fig. 2
CAMPEDELLI_2024 / CAMPEDELLI_2024_PEAK_SENSITIVITY	SENSITIVITY_ONLY; SENSITIVITY_ONLY	Stryker Sustainability Solutions pulse oximeter; Index finger primarily; earlobe or forehead if required	ABL 90 Flex Plus co-oximetry; direct arterial SaO2	peak exercise; sensitivity analysis excluding three supra-physiological cardiac-output outliers	265 / 265	-0.11	95% confidence interval -0.90 to 0.67; SD 6.49	One peak pair per participant	Source reports SaO2 - SpO2 = +0.11 (95% CI -0.67 to +0.90). The sign-harmonized SpO2 - SaO2 CI is -0.90 to +0.67. Paired-difference SD

Study / outcome	Synthesis role/status	Device / site	Arterial reference	Exercise time point or condition	N participants / pairs	Standardized bias	Reported precision or interval	Unit of analysis	Notes and source pages
									approximately 6.49 was reconstructed from the CI width and n=265. This participant-overlapping sensitivity analysis is not an independent effect.; Results; Fig. 2
ROBERTS_1999 / ROBERTS_1999_ALL_REST_EXERCISE	PRIMARY_CANDIDATE; NOT_POOLABLE_MIXED_REST_EXERCISE	Nellcor N200 with RS-10 reflective sensor; Forehead	IL 482 four-wavelength co-oximeter; measured arterial oxyhaemoglobin saturation	combined rest, supramaximal exercise, and recovery	8 / 65	0.1	limits -3.7 to 3.9	Repeated arterial/pulse-oximeter pairs from eight athletes	Published overall Bland-Altman limits in source direction were -3.9 to +3.7; standardized orientation reverses them. The reported SE/CI and limits are not used to derive an SD.; p.489; Fig. 2
ROBERTS_1999 / ROBERTS_1999_REST	SECONDARY_CONTEXT_ONLY; OUTSIDE_PRIMARY_EXERCISE_TIMEPOINT	Nellcor N200 with RS-10 reflective sensor; Forehead	IL 482 four-wavelength co-oximeter; measured arterial oxyhaemoglobin saturation	rest	8 / 8	0.7	SD 1.4	One rest observation per arterial-validation participant	Retained as context only; the primary review synthesis is restricted to exercise or immediate recovery.; p.489, Table 5
ROBERTS_1999 / ROBERTS_1999_MINIMUM_SAO2	PRIMARY_CANDIDATE; CONDITIONAL_MULTILEVEL_ONLY	Nellcor N200 with RS-10 reflective sensor; Forehead	IL 482 four-wavelength co-oximeter; measured arterial oxyhaemoglobin saturation	minimum SaO2 during the supramaximal exercise protocol	8 / 8	-1.2	SD 1.1	One minimum-saturation value per arterial-validation participant	Same eight participants as other Table 5 rows; must not be combined independently with the aggregate or threshold rows.; p.489, Table 5
ROBERTS_1999 / ROBERTS_1999_SAO2_GT_91	SENSITIVITY_ONLY; CONDITIONAL_MULTILEVEL_ONLY	Nellcor N200 with RS-10 reflective sensor; Forehead	IL 482 four-wavelength co-oximeter; measured arterial oxyhaemoglobin saturation	observations with SaO2 >91% across protocol	8 / 41	0.6	SD 1.8	Repeated observations; conditionally selected by reference saturation	Overlaps with all other Table 5 categories and with overall agreement data.; p.489, Table 5
ROBERTS_1999 / ROBERTS_1999_SAO2_LE_91	SENSITIVITY_ONLY; CONDITIONAL_MULTILEVEL_ONLY	Nellcor N200 with RS-10 reflective sensor; Forehead	IL 482 four-wavelength co-oximeter; measured arterial oxyhaemoglobin saturation	observations with SaO2 <=91% across protocol	8 / 24	-1.4	SD 2.1	Repeated observations; conditionally selected by reference saturation	Overlaps with the SaO2 <=86% subgroup and with overall agreement data.; p.489, Table 5
ROBERTS_1999 / ROBERTS_1999_SAO2_LE_86	SENSITIVITY_ONLY; CONDITIONAL_MULTILEVEL_ONLY	Nellcor N200 with RS-10 reflective sensor; Forehead	IL 482 four-wavelength co-oximeter; measured arterial oxyhaemoglobin saturation	observations with SaO2 <=86% across protocol	8 / 7	-2.3	SD 3.1	Repeated observations; conditionally selected by reference saturation	Nested within the SaO2 <=91% row; do not treat as an independent effect.; p.489, Table 5
ASCHA_2018 / ASCHA_2018_PEAK	PRIMARY_CANDIDATE;	Nonin Avant 4000; Forehead	ABL800 FLEX Blood Gas Analyzer	peak exercise; SpO2 recorded at arterial blood-gas acquisition	631 / 631	2.63	SD 2.75; limits -2.9 to 8.1	One peak-exercise pair per participant	Reported paired mean (SD) difference; source master has a rounded 2.6-point value. Values

Study / outcome	Synthesis role/status	Device / site	Arterial reference	Exercise time point or condition	N particip ants / pairs	Standa rdized bias	Reported precision or interval	Unit of analysis	Notes and source pages
	PRIMARY_STU DY_LEVEL		(Radiometer) co-oximetry; direct arterial SaO2						are preserved at table precision.; Table 2; peak exercise
SWIGRIS_200 9 / SWIGRIS_200 9_MAX_EXER CISE	PRIMARY_CAN DIDATE; PRIMARY_STU DY_LEVEL	Peripheral pulse oximeter; model changed over the three-decade cohort; Index finger primary; earlobe backup	Arterial blood analysed with a co-oximeter plus blood-gas analyser; direct SaO2	maximum exercise; per-subject comparison	83 / 83	1.5	SD 4.0	One maximum-exercise pair per participant	Signed mean (SD) difference; device and co-oximeter models changed over the study period and earlobe was used if a finger signal was inadequate.; Results; Fig. 1

e-Table S5. Source-based descriptive methodological appraisal

No aggregate risk-of-bias grades are assigned. Source evidence and applicability are retained independently of the historical any-No algorithm.

Study	Applicability / context	Key source evidence	Evidence location	Interpretive limitation
ASCHA_2018	Population: Moderate; Index: Low; Reference: Moderate	Retrospective referral cohort; 631 of 751 had complete peak pairs; fixed forehead Nonin device with waveform/heart-rate synchronization; direct ABL800 co-oximetry; peak SpO2 recorded at arterial sampling. The report identifies	Methods; Table 2 Methods, Pulse Oximetry and Arterial Oxygenation Determinations (article p. 1993); Table 2	Complete-pair restriction could affect representativeness, but the retained outcome is one contemporaneous peak pair per analysed participant. The source confirms direct co-oximetry but leaves the

		ABL800 FLEX co-oximetry and calls the result SaO2, but does not define whether that saturation is functional or fractional.		functional/fractional target incompletely specified.
SWIGRIS_2009	Population: High; Index: High; Reference: Moderate	Retrospective single-centre systemic sclerosis-associated interstitial lung disease cohort at Denver altitude; pulse-oximeter and co-oximeter models changed over three decades and earlobe was used after inadequate finger signal; arterial samples were analysed by co-oximetry; one maximal-exercise comparison per subject. The report states that	Methods; Results; Fig. 1 Methods, Maximal cardiopulmonary exercise test (article p. 255); Fig. 1	Device/site heterogeneity precludes a stable index-test estimate even though the reference is direct. The source confirms direct co-oximetry but leaves the functional/fractional target incompletely specified.

		arterial blood was analysed with a co-oximeter for SaO2, but does not define whether the reported saturation is functional or fractional.		
ZEBALLOS_1991	Population: High; Index: Moderate; Reference: Low	Young Black male recruits; specified ear devices and IL282 functional co-oximetry; published device-by-altitude estimates combine rest, repeated exercise stages, and recovery; condition-specific pair counts are unavailable.	Methods; pp. 1241-1243	No study outcome is restricted to the protocol's exercise-only analytical unit.
YAMAYA_2002	Population: High; Index: Moderate; Reference: Low	Small mixed healthy/athlete/clinical sample; three device-site combinations;	Methods; p. 165	The all-data primary estimates cannot be treated as

		IL682 co-oximetry; reported estimates combine repeated rest/exercise and normoxia/hypoxia observations without recoverable within-person covariance.		independent participant-level observations.
HANSEN_1987	Population: Moderate; Index: Moderate; Reference: Low	Consecutive clinical exercise-test patients; single Biox IIA ear device; IL282 four-wavelength co-oximetry; serial results are graphical group means with SE rather than an extractable paired agreement analysis.	Methods; p. 335	Non-extractability limits precision synthesis but does not itself establish biased measurement.

BENOIT_1997	Population: High; Index: High; Reference: Low	Ten healthy men; radial arterial OSM3 co-oximetry; reported agreement uses a post-hoc corrected SpO2 equation rather than raw device output and contains repeated measurements in saturation strata.	Methods; p. 261	Retained only as technology-history/sensitivity evidence; not valid for the raw-SpO2 primary comparison.
MCGOVERN_1996	Population: High; Index: Moderate; Reference: Low	Eight severe-COPD participants; fixed Ohmeda finger device with signal-quality review; IL482 co-oximetry; 49 exercise pairs from eight participants with no recoverable clustering for the reported precision.	Methods and Results; p. 1153	The raw device result is eligible, but repeated pairs cannot be treated as 49 independent observations.

CARONE_1997	Population: High; Index: Moderate; Reference: Low	COPD patients receiving long-term oxygen therapy; fixed Biox ear device and IL282 co-oximetry; agreement set combines baseline, repeated walking time points, oxygen titration, and repeated tests; number of agreement pairs is not reported.	Methods and Results; p. 448	The 47 sixth-minute clinical-cutoff pairs are not the Bland-Altman agreement denominator.
MARTIN_1992	Population: High; Index: Moderate; Reference: High	Eleven elite male cyclists; specified simultaneous ear/finger devices; IL282 directly measured fractional oxyhaemoglobin; 232 repeated exercise observations per	Methods and Results; p. 456	Direct fractional HbO2 is not silently interchangeable with functional SaO2 for the review's primary target.

		probe and three correlated device-site outcomes.		
BARTHELEMY_1990	Population: Moderate; Index: High; Reference: Low	Fifty-seven adults; eight finger-device comparisons; OSM3 functional co-oximetry; device-specific sample counts are repeated observations rather than independent participants.	Methods and Results; p. 535	No prespecified single index device and no within-subject covariance are available.
POWERS_1989	Population: High; Index: Moderate; Reference: High	Ten healthy men; three simultaneous device/site configurations; synchronized radial samples and IL282 direct fractional HbO2; highest-work result gives one	Methods; Table 1; p. 301	A condition-specific estimate is structurally usable only after choosing one configuration; fractional HbO2 creates a reference-definition applicability concern.

		pair per participant for each correlated configuration.		
RIES_1985	Population: Moderate; Index: High; Reference: Low	Pulmonary-laboratory patients across three device phases; radial samples within five seconds and IL282 direct co-oximetry; repeated exercise samples overlap across phases and reported SEE is not paired-difference SD.	Methods; Tables 1-3; pp. 686-688	Exercise-only direction is available, but the variance and independence conditions for pooling are not.
RICE_1999	Population: High; Index: Moderate; Reference: Moderate	Fifteen competitive male cyclists; fixed ear device and synchronized radial sampling; the report names ABL520 for SaO2 but provides limited direct-method detail;	Methods; Fig. 4	External technical verification supported eligibility; the article's graphical result remains qualitative.

		comparison is graphical across rest and repeated exercise stages.		
LUNDGREN_2001	Population: High; Index: High; Reference: Moderate	Retrospective beryllium sensitization/chronic beryllium disease cardiopulmonary exercise testing cohort; two pulse-oximeter generations and two co-oximeter platforms are combined across eras; one maximum-exercise pair per participant in the beryllium sensitization estimate. The report identifies two co-oximeter generations and calls the result SaO2, but does not define whether the reported	Methods and Results; pp. 1702 and 1705 Methods, Exercise Physiology Evaluation (article p. 1704)	The beryllium sensitization estimate has a paired SD, but an unstratified mixed index technology limits a common target effect. The source confirms direct co-oximetry but leaves the functional/fractional target incompletely specified.

		saturation is functional or fractional.		
CAMPEDELLI_2024	Population: Moderate; Index: Moderate; Reference: Moderate	Consecutive invasive-cardiopulmonary exercise testing cohort; ABL90 direct co-oximetry and exact blood-draw timing; peak analysis has one pair per participant, but signal failure can change sensor site and 28 patients lack complete peak data; reported CI is labelled as a limit of agreement. The report identifies ABL90 Flex Plus co-oximetry and reports SaO2/oxyhaemoglobin alongside dyshemoglobins, but	Methods; Results; Fig. 2 Methods, SO2 measurements (PDF p. 3; article p. 4)	The point estimate is usable descriptively; the reported spread is not treated as a trusted paired-difference variance. The source confirms direct co-oximetry but leaves the functional/fractional target incompletely specified.

		does not explicitly define a functional or fractional saturation calculation.		
ROBERTS_1999	Population: High; Index: Moderate; Reference: Low	Only eight athlete volunteers formed the arterial-validation substudy; fixed forehead device and IL482 co-oximetry; 65 repeated pairs mix rest/exercise/recovery and saturation-threshold rows overlap.	Methods and Results; Fig. 2 and Table 5; p. 489	The minimum-SaO2 row is conditionally defined and must not be combined independently with the aggregate or threshold rows.

e-Table S6. Unchanged author-agreed item responses and source evidence

Both authors checked and agreed these 192 final responses. Separate independent forms are not available. Item functions below are interpretive labels, not new human ratings or validation of the framework; original aggregate labels are preserved only in the provenance archive. Item-type labels: E/R=eligibility or reference-construct reporting; B=potential selection/missingness/dependence mechanism; B/R=reporting or construct clarity that may relate to bias. These are interpretive functions, not new risk ratings.

Study ID	Author-year	Domain	Item	Signaling question	Final response	Rationale and study evidence	Evidence location	Item type
ASCHA_2018	Ascha et al., 2018	D1	D1Q1	Was enrollment consecutive/random or based on a clearly defined cohort without case-control enrichment?	Unclear	The archived report-level evidence does not establish this condition clearly.; Retrospective referral cohort; 631 of 751 had complete peak pairs; fixed forehead Nonin device with waveform/heart-rate synchronization; direct ABL800 co-oximetry; peak SpO2 recorded at arterial sampling.	Methods; Table 2	B
ASCHA_2018	Ascha et al., 2018	D1	D1Q2	Were exclusions and missing participants unlikely to depend on the index or reference result?	Yes	The archived report-level evidence supports the low-risk condition.; Retrospective referral cohort; 631 of 751 had complete peak pairs; fixed forehead Nonin device with waveform/heart-rate synchronization; direct ABL800 co-oximetry; peak SpO2 recorded at arterial sampling.	Methods; Table 2	B
ASCHA_2018	Ascha et al., 2018	D1	D1Q3	Was the analyzed comparison not restricted to a selectively chosen validation subgroup?	Yes	The archived report-level evidence supports the low-risk condition.; Retrospective referral cohort; 631 of 751 had complete peak pairs; fixed forehead Nonin device with waveform/heart-rate synchronization; direct ABL800 co-oximetry; peak SpO2 recorded at arterial sampling.	Methods; Table 2	B/R
ASCHA_2018	Ascha et al., 2018	D2	D2Q1	Were device, site, configuration/averaging, and changes sufficiently reported and stable?	Yes	The archived report-level evidence supports the low-risk condition.; Retrospective referral cohort; 631 of 751 had complete peak pairs; fixed forehead Nonin device with waveform/heart-rate synchronization; direct ABL800 co-oximetry; peak SpO2 recorded at arterial sampling.	Methods; Table 2	B/R
ASCHA_2018	Ascha et al., 2018	D2	D2Q2	Was the analyzed index the raw SpO2 rather than a post hoc corrected or secondary index?	Yes	The archived report-level evidence supports the low-risk condition.; Retrospective referral cohort; 631 of 751 had complete peak pairs; fixed forehead Nonin device with waveform/heart-rate synchronization; direct ABL800 co-oximetry; peak SpO2 recorded at arterial sampling.	Methods; Table 2	B/R
ASCHA_2018	Ascha et al., 2018	D2	D2Q3	Were signal-quality handling, failed readings, and index selection handled without evident result-dependent selection?	Yes	The archived report-level evidence supports the low-risk condition.; Retrospective referral cohort; 631 of 751 had complete peak pairs; fixed forehead Nonin device with waveform/heart-rate synchronization; direct ABL800 co-oximetry; peak SpO2 recorded at arterial sampling.	Methods; Table 2	B
ASCHA_2018	Ascha et al., 2018	D3	D3Q1	Was SaO2 obtained from a direct arterial sample using multiwavelength co-oximetry?	Yes	The archived report-level evidence supports the low-risk condition.; Retrospective referral cohort; 631 of 751 had complete peak pairs; fixed forehead Nonin device with waveform/heart-rate synchronization; direct ABL800 co-oximetry; peak SpO2 recorded at arterial sampling.	Methods; Table 2	E/R

Study ID	Author-year	Domain	Item	Signaling question	Final response	Rationale and study evidence	Evidence location	Item type
ASCHA_2018	Ascha et al., 2018	D3	D3Q2	Was the functional/fractional saturation target clear and internally noncontradictory?	Unclear	Direct arterial co-oximetry is confirmed, but the functional/fractional saturation target is not explicitly defined; the review-defined D3Q2 condition is therefore unclear.; Retrospective referral cohort; 631 of 751 had complete peak pairs; fixed forehead Nonin device with waveform/heart-rate synchronization; direct ABL800 co-oximetry; peak SpO2 recorded at arterial sampling. The report identifies ABL800 FLEX co-oximetry and calls the result SaO2, but does not define whether that saturation is functional or fractional.	Methods, Pulse Oximetry and Arterial Oxygenation Determinations (article p. 1993); Table 2	B/R
ASCHA_2018	Ascha et al., 2018	D3	D3Q3	Was the reference result generated independently of the index result?	Yes	The archived report-level evidence supports the low-risk condition.; Retrospective referral cohort; 631 of 751 had complete peak pairs; fixed forehead Nonin device with waveform/heart-rate synchronization; direct ABL800 co-oximetry; peak SpO2 recorded at arterial sampling.	Methods; Table 2	B
ASCHA_2018	Ascha et al., 2018	D4	D4Q1	Were SpO2 and SaO2 paired contemporaneously during exercise or within two minutes after cessation?	Yes	The archived report-level evidence supports the low-risk condition.; Retrospective referral cohort; 631 of 751 had complete peak pairs; fixed forehead Nonin device with waveform/heart-rate synchronization; direct ABL800 co-oximetry; peak SpO2 recorded at arterial sampling.	Methods; Table 2	B/R
ASCHA_2018	Ascha et al., 2018	D4	D4Q2	Were eligible pairs included representatively and missing pairs or failed readings explained?	Unclear	The archived report-level evidence does not establish this condition clearly.; Retrospective referral cohort; 631 of 751 had complete peak pairs; fixed forehead Nonin device with waveform/heart-rate synchronization; direct ABL800 co-oximetry; peak SpO2 recorded at arterial sampling.	Methods; Table 2	B
ASCHA_2018	Ascha et al., 2018	D4	D4Q3	Did the analysis account for repeated measures, multiple devices/sites, and mixed periods?	Yes	The archived report-level evidence supports the low-risk condition.; Retrospective referral cohort; 631 of 751 had complete peak pairs; fixed forehead Nonin device with waveform/heart-rate synchronization; direct ABL800 co-oximetry; peak SpO2 recorded at arterial sampling.	Methods; Table 2	B
SWIGRIS_2009	Swigris et al., 2009	D1	D1Q1	Was enrollment consecutive/random or based on a clearly defined cohort without case-control enrichment?	Unclear	The archived report-level evidence does not establish this condition clearly.; Retrospective single-centre systemic sclerosis-associated interstitial lung disease cohort at Denver altitude; pulse-oximeter and co-oximeter models changed over three decades and earlobe was used after inadequate finger signal; arterial samples were	Methods; Results; Fig. 1	B

Study ID	Author-year	Domain	Item	Signaling question	Final response	Rationale and study evidence	Evidence location	Item type
						analysed by co-oximetry; one maximal-exercise comparison per subject.		
SWIGRIS_ 2009	Swigris et al., 2009	D1	D1Q2	Were exclusions and missing participants unlikely to depend on the index or reference result?	Yes	The archived report-level evidence supports the low-risk condition.; Retrospective single-centre systemic sclerosis-associated interstitial lung disease cohort at Denver altitude; pulse-oximeter and co-oximeter models changed over three decades and earlobe was used after inadequate finger signal; arterial samples were analysed by co-oximetry; one maximal-exercise comparison per subject.	Methods; Results; Fig. 1	B
SWIGRIS_ 2009	Swigris et al., 2009	D1	D1Q3	Was the analyzed comparison not restricted to a selectively chosen validation subgroup?	Yes	The archived report-level evidence supports the low-risk condition.; Retrospective single-centre systemic sclerosis-associated interstitial lung disease cohort at Denver altitude; pulse-oximeter and co-oximeter models changed over three decades and earlobe was used after inadequate finger signal; arterial samples were analysed by co-oximetry; one maximal-exercise comparison per subject.	Methods; Results; Fig. 1	B/R
SWIGRIS_ 2009	Swigris et al., 2009	D2	D2Q1	Were device, site, configuration/averaging, and changes sufficiently reported and stable?	No	The archived report-level evidence documents a failure or contradiction for this condition.; Retrospective single-centre systemic sclerosis-associated interstitial lung disease cohort at Denver altitude; pulse-oximeter and co-oximeter models changed over three decades and earlobe was used after inadequate finger signal; arterial samples were analysed by co-oximetry; one maximal-exercise comparison per subject.	Methods; Results; Fig. 1	B/R
SWIGRIS_ 2009	Swigris et al., 2009	D2	D2Q2	Was the analyzed index the raw SpO2 rather than a post hoc corrected or secondary index?	Yes	The archived report-level evidence supports the low-risk condition.; Retrospective single-centre systemic sclerosis-associated interstitial lung disease cohort at Denver altitude; pulse-oximeter and co-oximeter models changed over three decades and earlobe was used after inadequate finger signal; arterial samples were analysed by co-oximetry; one maximal-exercise comparison per subject.	Methods; Results; Fig. 1	B/R
SWIGRIS_ 2009	Swigris et al., 2009	D2	D2Q3	Were signal-quality handling, failed readings, and index selection handled without evident result-dependent selection?	No	The archived report-level evidence documents a failure or contradiction for this condition.; Retrospective single-centre systemic sclerosis-associated interstitial lung disease cohort at Denver altitude; pulse-oximeter and co-oximeter models changed over three decades and earlobe	Methods; Results; Fig. 1	B

Study ID	Author-year	Domain	Item	Signaling question	Final response	Rationale and study evidence	Evidence location	Item type
						was used after inadequate finger signal; arterial samples were analysed by co-oximetry; one maximal-exercise comparison per subject.		
SWIGRIS_2009	Swigris et al., 2009	D3	D3Q1	Was SaO2 obtained from a direct arterial sample using multiwavelength co-oximetry?	Yes	The archived report-level evidence supports the low-risk condition.; Retrospective single-centre systemic sclerosis-associated interstitial lung disease cohort at Denver altitude; pulse-oximeter and co-oximeter models changed over three decades and earlobe was used after inadequate finger signal; arterial samples were analysed by co-oximetry; one maximal-exercise comparison per subject.	Methods; Results; Fig. 1	E/R
SWIGRIS_2009	Swigris et al., 2009	D3	D3Q2	Was the functional/fractional saturation target clear and internally noncontradictory?	Unclear	Direct arterial co-oximetry is confirmed, but the functional/fractional saturation target is not explicitly defined; the review-defined D3Q2 condition is therefore unclear.; Retrospective single-centre systemic sclerosis-associated interstitial lung disease cohort at Denver altitude; pulse-oximeter and co-oximeter models changed over three decades and earlobe was used after inadequate finger signal; arterial samples were analysed by co-oximetry; one maximal-exercise comparison per subject. The report states that arterial blood was analysed with a co-oximeter for SaO2, but does not define whether the reported saturation is functional or fractional.	Methods, Maximal cardiopulmonary exercise test (article p. 255); Fig. 1	B/R
SWIGRIS_2009	Swigris et al., 2009	D3	D3Q3	Was the reference result generated independently of the index result?	Yes	The archived report-level evidence supports the low-risk condition.; Retrospective single-centre systemic sclerosis-associated interstitial lung disease cohort at Denver altitude; pulse-oximeter and co-oximeter models changed over three decades and earlobe was used after inadequate finger signal; arterial samples were analysed by co-oximetry; one maximal-exercise comparison per subject.	Methods; Results; Fig. 1	B
SWIGRIS_2009	Swigris et al., 2009	D4	D4Q1	Were SpO2 and SaO2 paired contemporaneously during exercise or within two minutes after cessation?	Yes	The archived report-level evidence supports the low-risk condition.; Retrospective single-centre systemic sclerosis-associated interstitial lung disease cohort at Denver altitude; pulse-oximeter and co-oximeter models changed over three decades and earlobe was used after inadequate finger signal; arterial samples were analysed by co-oximetry; one maximal-exercise comparison per subject.	Methods; Results; Fig. 1	B/R

Study ID	Author-year	Domain	Item	Signaling question	Final response	Rationale and study evidence	Evidence location	Item type
SWIGRIS_ 2009	Swigris et al., 2009	D4	D4Q2	Were eligible pairs included representatively and missing pairs or failed readings explained?	Unclear	The archived report-level evidence does not establish this condition clearly.; Retrospective single-centre systemic sclerosis-associated interstitial lung disease cohort at Denver altitude; pulse-oximeter and co-oximeter models changed over three decades and earlobe was used after inadequate finger signal; arterial samples were analysed by co-oximetry; one maximal-exercise comparison per subject.	Methods; Results; Fig. 1	B
SWIGRIS_ 2009	Swigris et al., 2009	D4	D4Q3	Did the analysis account for repeated measures, multiple devices/sites, and mixed periods?	Yes	The archived report-level evidence supports the low-risk condition.; Retrospective single-centre systemic sclerosis-associated interstitial lung disease cohort at Denver altitude; pulse-oximeter and co-oximeter models changed over three decades and earlobe was used after inadequate finger signal; arterial samples were analysed by co-oximetry; one maximal-exercise comparison per subject.	Methods; Results; Fig. 1	B
ZEBALLO S_1991	Zeballos et al., 1991	D1	D1Q1	Was enrollment consecutive/random or based on a clearly defined cohort without case-control enrichment?	Unclear	The archived report-level evidence does not establish this condition clearly.; Young Black male recruits; specified ear devices and IL282 functional co-oximetry; published device-by-altitude estimates combine rest, repeated exercise stages, and recovery; condition-specific pair counts are unavailable.	Methods; pp. 1241-1243	B
ZEBALLO S_1991	Zeballos et al., 1991	D1	D1Q2	Were exclusions and missing participants unlikely to depend on the index or reference result?	Yes	The archived report-level evidence supports the low-risk condition.; Young Black male recruits; specified ear devices and IL282 functional co-oximetry; published device-by-altitude estimates combine rest, repeated exercise stages, and recovery; condition-specific pair counts are unavailable.	Methods; pp. 1241-1243	B
ZEBALLO S_1991	Zeballos et al., 1991	D1	D1Q3	Was the analyzed comparison not restricted to a selectively chosen validation subgroup?	Yes	The archived report-level evidence supports the low-risk condition.; Young Black male recruits; specified ear devices and IL282 functional co-oximetry; published device-by-altitude estimates combine rest, repeated exercise stages, and recovery; condition-specific pair counts are unavailable.	Methods; pp. 1241-1243	B/R
ZEBALLO S_1991	Zeballos et al., 1991	D2	D2Q1	Were device, site, configuration/averaging, and changes sufficiently reported and stable?	Yes	The archived report-level evidence supports the low-risk condition.; Young Black male recruits; specified ear devices and IL282 functional co-oximetry; published device-by-altitude estimates combine rest, repeated exercise stages, and	Methods; pp. 1241-1243	B/R

Study ID	Author-year	Domain	Item	Signaling question	Final response	Rationale and study evidence	Evidence location	Item type
						recovery; condition-specific pair counts are unavailable.		
ZEBALLOS_1991	Zeballos et al., 1991	D2	D2Q2	Was the analyzed index the raw SpO2 rather than a post hoc corrected or secondary index?	Yes	The archived report-level evidence supports the low-risk condition.; Young Black male recruits; specified ear devices and IL282 functional co-oximetry; published device-by-altitude estimates combine rest, repeated exercise stages, and recovery; condition-specific pair counts are unavailable.	Methods; pp. 1241-1243	B/R
ZEBALLOS_1991	Zeballos et al., 1991	D2	D2Q3	Were signal-quality handling, failed readings, and index selection handled without evident result-dependent selection?	Yes	The archived report-level evidence supports the low-risk condition.; Young Black male recruits; specified ear devices and IL282 functional co-oximetry; published device-by-altitude estimates combine rest, repeated exercise stages, and recovery; condition-specific pair counts are unavailable.	Methods; pp. 1241-1243	B
ZEBALLOS_1991	Zeballos et al., 1991	D3	D3Q1	Was SaO2 obtained from a direct arterial sample using multiwavelength co-oximetry?	Yes	The archived report-level evidence supports the low-risk condition.; Young Black male recruits; specified ear devices and IL282 functional co-oximetry; published device-by-altitude estimates combine rest, repeated exercise stages, and recovery; condition-specific pair counts are unavailable.	Methods; pp. 1241-1243	E/R
ZEBALLOS_1991	Zeballos et al., 1991	D3	D3Q2	Was the functional/fractional saturation target clear and internally noncontradictory?	Yes	The archived report-level evidence supports the low-risk condition.; Young Black male recruits; specified ear devices and IL282 functional co-oximetry; published device-by-altitude estimates combine rest, repeated exercise stages, and recovery; condition-specific pair counts are unavailable.	Methods; pp. 1241-1243	B/R
ZEBALLOS_1991	Zeballos et al., 1991	D3	D3Q3	Was the reference result generated independently of the index result?	Yes	The archived report-level evidence supports the low-risk condition.; Young Black male recruits; specified ear devices and IL282 functional co-oximetry; published device-by-altitude estimates combine rest, repeated exercise stages, and recovery; condition-specific pair counts are unavailable.	Methods; pp. 1241-1243	B
ZEBALLOS_1991	Zeballos et al., 1991	D4	D4Q1	Were SpO2 and SaO2 paired contemporaneously during exercise or within two minutes after cessation?	Yes	The archived report-level evidence supports the low-risk condition.; Young Black male recruits; specified ear devices and IL282 functional co-oximetry; published device-by-altitude estimates combine rest, repeated exercise stages, and	Methods; pp. 1241-1243	B/R

Study ID	Author-year	Domain	Item	Signaling question	Final response	Rationale and study evidence	Evidence location	Item type
						recovery; condition-specific pair counts are unavailable.		
ZEBALLOS_1991	Zeballos et al., 1991	D4	D4Q2	Were eligible pairs included representatively and missing pairs or failed readings explained?	Unclear	The archived report-level evidence does not establish this condition clearly.; Young Black male recruits; specified ear devices and IL282 functional co-oximetry; published device-by-altitude estimates combine rest, repeated exercise stages, and recovery; condition-specific pair counts are unavailable.	Methods; pp. 1241-1243	B
ZEBALLOS_1991	Zeballos et al., 1991	D4	D4Q3	Did the analysis account for repeated measures, multiple devices/sites, and mixed periods?	No	The archived report-level evidence documents a failure or contradiction for this condition.; Young Black male recruits; specified ear devices and IL282 functional co-oximetry; published device-by-altitude estimates combine rest, repeated exercise stages, and recovery; condition-specific pair counts are unavailable.	Methods; pp. 1241-1243	B
YAMAYA_2002	Yamaya et al., 2002	D1	D1Q1	Was enrollment consecutive/random or based on a clearly defined cohort without case-control enrichment?	Unclear	The archived report-level evidence does not establish this condition clearly.; Small mixed healthy/athlete/clinical sample; three device-site combinations; IL682 co-oximetry; reported estimates combine repeated rest/exercise and normoxia/hypoxia observations without recoverable within-person covariance.	Methods; p. 165	B
YAMAYA_2002	Yamaya et al., 2002	D1	D1Q2	Were exclusions and missing participants unlikely to depend on the index or reference result?	Yes	The archived report-level evidence supports the low-risk condition.; Small mixed healthy/athlete/clinical sample; three device-site combinations; IL682 co-oximetry; reported estimates combine repeated rest/exercise and normoxia/hypoxia observations without recoverable within-person covariance.	Methods; p. 165	B
YAMAYA_2002	Yamaya et al., 2002	D1	D1Q3	Was the analyzed comparison not restricted to a selectively chosen validation subgroup?	Yes	The archived report-level evidence supports the low-risk condition.; Small mixed healthy/athlete/clinical sample; three device-site combinations; IL682 co-oximetry; reported estimates combine repeated rest/exercise and normoxia/hypoxia observations without recoverable within-person covariance.	Methods; p. 165	B/R
YAMAYA_2002	Yamaya et al., 2002	D2	D2Q1	Were device, site, configuration/averaging, and changes sufficiently reported and stable?	Yes	The archived report-level evidence supports the low-risk condition.; Small mixed healthy/athlete/clinical sample; three device-site combinations; IL682 co-oximetry; reported estimates combine repeated rest/exercise and	Methods; p. 165	B/R

Study ID	Author-year	Domain	Item	Signaling question	Final response	Rationale and study evidence	Evidence location	Item type
						normoxia/hypoxia observations without recoverable within-person covariance.		
YAMAYA _2002	Yamaya et al., 2002	D2	D2Q2	Was the analyzed index the raw SpO2 rather than a post hoc corrected or secondary index?	Yes	The archived report-level evidence supports the low-risk condition.; Small mixed healthy/athlete/clinical sample; three device-site combinations; IL682 co-oximetry; reported estimates combine repeated rest/exercise and normoxia/hypoxia observations without recoverable within-person covariance.	Methods; p. 165	B/R
YAMAYA _2002	Yamaya et al., 2002	D2	D2Q3	Were signal-quality handling, failed readings, and index selection handled without evident result-dependent selection?	Unclear	The archived report-level evidence does not establish this condition clearly.; Small mixed healthy/athlete/clinical sample; three device-site combinations; IL682 co-oximetry; reported estimates combine repeated rest/exercise and normoxia/hypoxia observations without recoverable within-person covariance.	Methods; p. 165	B
YAMAYA _2002	Yamaya et al., 2002	D3	D3Q1	Was SaO2 obtained from a direct arterial sample using multiwavelength co-oximetry?	Yes	The archived report-level evidence supports the low-risk condition.; Small mixed healthy/athlete/clinical sample; three device-site combinations; IL682 co-oximetry; reported estimates combine repeated rest/exercise and normoxia/hypoxia observations without recoverable within-person covariance.	Methods; p. 165	E/R
YAMAYA _2002	Yamaya et al., 2002	D3	D3Q2	Was the functional/fractional saturation target clear and internally noncontradictory?	Yes	The archived report-level evidence supports the low-risk condition.; Small mixed healthy/athlete/clinical sample; three device-site combinations; IL682 co-oximetry; reported estimates combine repeated rest/exercise and normoxia/hypoxia observations without recoverable within-person covariance.	Methods; p. 165	B/R
YAMAYA _2002	Yamaya et al., 2002	D3	D3Q3	Was the reference result generated independently of the index result?	Yes	The archived report-level evidence supports the low-risk condition.; Small mixed healthy/athlete/clinical sample; three device-site combinations; IL682 co-oximetry; reported estimates combine repeated rest/exercise and normoxia/hypoxia observations without recoverable within-person covariance.	Methods; p. 165	B
YAMAYA _2002	Yamaya et al., 2002	D4	D4Q1	Were SpO2 and SaO2 paired contemporaneously during exercise or within two minutes after cessation?	Yes	The archived report-level evidence supports the low-risk condition.; Small mixed healthy/athlete/clinical sample; three device-site combinations; IL682 co-oximetry; reported estimates combine repeated rest/exercise and	Methods; p. 165	B/R

Study ID	Author-year	Domain	Item	Signaling question	Final response	Rationale and study evidence	Evidence location	Item type
						normoxia/hypoxia observations without recoverable within-person covariance.		
YAMAYA_2002	Yamaya et al., 2002	D4	D4Q2	Were eligible pairs included representatively and missing pairs or failed readings explained?	Unclear	The archived report-level evidence does not establish this condition clearly.; Small mixed healthy/athlete/clinical sample; three device-site combinations; IL682 co-oximetry; reported estimates combine repeated rest/exercise and normoxia/hypoxia observations without recoverable within-person covariance.	Methods; p. 165	B
YAMAYA_2002	Yamaya et al., 2002	D4	D4Q3	Did the analysis account for repeated measures, multiple devices/sites, and mixed periods?	No	The archived report-level evidence documents a failure or contradiction for this condition.; Small mixed healthy/athlete/clinical sample; three device-site combinations; IL682 co-oximetry; reported estimates combine repeated rest/exercise and normoxia/hypoxia observations without recoverable within-person covariance.	Methods; p. 165	B
HANSEN_1987	Hansen et al., 1987	D1	D1Q1	Was enrollment consecutive/random or based on a clearly defined cohort without case-control enrichment?	Yes	The archived report-level evidence supports the low-risk condition.; Consecutive clinical exercise-test patients; single Biox IIA ear device; IL282 four-wavelength co-oximetry; serial results are graphical group means with SE rather than an extractable paired agreement analysis.	Methods; p. 335	B
HANSEN_1987	Hansen et al., 1987	D1	D1Q2	Were exclusions and missing participants unlikely to depend on the index or reference result?	Yes	The archived report-level evidence supports the low-risk condition.; Consecutive clinical exercise-test patients; single Biox IIA ear device; IL282 four-wavelength co-oximetry; serial results are graphical group means with SE rather than an extractable paired agreement analysis.	Methods; p. 335	B
HANSEN_1987	Hansen et al., 1987	D1	D1Q3	Was the analyzed comparison not restricted to a selectively chosen validation subgroup?	Yes	The archived report-level evidence supports the low-risk condition.; Consecutive clinical exercise-test patients; single Biox IIA ear device; IL282 four-wavelength co-oximetry; serial results are graphical group means with SE rather than an extractable paired agreement analysis.	Methods; p. 335	B/R
HANSEN_1987	Hansen et al., 1987	D2	D2Q1	Were device, site, configuration/averaging, and changes sufficiently reported and stable?	Yes	The archived report-level evidence supports the low-risk condition.; Consecutive clinical exercise-test patients; single Biox IIA ear device; IL282 four-wavelength co-oximetry; serial results are graphical group means with SE rather than an extractable paired agreement analysis.	Methods; p. 335	B/R

Study ID	Author-year	Domain	Item	Signaling question	Final response	Rationale and study evidence	Evidence location	Item type
HANSEN_ 1987	Hansen et al., 1987	D2	D2Q2	Was the analyzed index the raw SpO2 rather than a post hoc corrected or secondary index?	Yes	The archived report-level evidence supports the low-risk condition.; Consecutive clinical exercise-test patients; single Biox IIA ear device; IL282 four-wavelength co-oximetry; serial results are graphical group means with SE rather than an extractable paired agreement analysis.	Methods; p. 335	B/R
HANSEN_ 1987	Hansen et al., 1987	D2	D2Q3	Were signal-quality handling, failed readings, and index selection handled without evident result-dependent selection?	Yes	The archived report-level evidence supports the low-risk condition.; Consecutive clinical exercise-test patients; single Biox IIA ear device; IL282 four-wavelength co-oximetry; serial results are graphical group means with SE rather than an extractable paired agreement analysis.	Methods; p. 335	B
HANSEN_ 1987	Hansen et al., 1987	D3	D3Q1	Was SaO2 obtained from a direct arterial sample using multiwavelength co-oximetry?	Yes	The archived report-level evidence supports the low-risk condition.; Consecutive clinical exercise-test patients; single Biox IIA ear device; IL282 four-wavelength co-oximetry; serial results are graphical group means with SE rather than an extractable paired agreement analysis.	Methods; p. 335	E/R
HANSEN_ 1987	Hansen et al., 1987	D3	D3Q2	Was the functional/fractional saturation target clear and internally noncontradictory?	Yes	The archived report-level evidence supports the low-risk condition.; Consecutive clinical exercise-test patients; single Biox IIA ear device; IL282 four-wavelength co-oximetry; serial results are graphical group means with SE rather than an extractable paired agreement analysis.	Methods; p. 335	B/R
HANSEN_ 1987	Hansen et al., 1987	D3	D3Q3	Was the reference result generated independently of the index result?	Yes	The archived report-level evidence supports the low-risk condition.; Consecutive clinical exercise-test patients; single Biox IIA ear device; IL282 four-wavelength co-oximetry; serial results are graphical group means with SE rather than an extractable paired agreement analysis.	Methods; p. 335	B
HANSEN_ 1987	Hansen et al., 1987	D4	D4Q1	Were SpO2 and SaO2 paired contemporaneously during exercise or within two minutes after cessation?	Yes	The archived report-level evidence supports the low-risk condition.; Consecutive clinical exercise-test patients; single Biox IIA ear device; IL282 four-wavelength co-oximetry; serial results are graphical group means with SE rather than an extractable paired agreement analysis.	Methods; p. 335	B/R
HANSEN_ 1987	Hansen et al., 1987	D4	D4Q2	Were eligible pairs included representatively and missing pairs or failed readings explained?	Yes	The archived report-level evidence supports the low-risk condition.; Consecutive clinical exercise-test patients; single Biox IIA ear device; IL282 four-wavelength co-oximetry; serial results are graphical group means with SE rather than an extractable paired agreement analysis.	Methods; p. 335	B

Study ID	Author-year	Domain	Item	Signaling question	Final response	Rationale and study evidence	Evidence location	Item type
HANSEN_ 1987	Hansen et al., 1987	D4	D4Q3	Did the analysis account for repeated measures, multiple devices/sites, and mixed periods?	Unclear	The archived report-level evidence does not establish this condition clearly.; Consecutive clinical exercise-test patients; single Biox IIA ear device; IL282 four-wavelength co-oximetry; serial results are graphical group means with SE rather than an extractable paired agreement analysis.	Methods; p. 335	B
BENOIT_1 997	Benoit et al., 1997	D1	D1Q1	Was enrollment consecutive/random or based on a clearly defined cohort without case-control enrichment?	Unclear	The archived report-level evidence does not establish this condition clearly.; Ten healthy men; radial arterial OSM3 co-oximetry; reported agreement uses a post-hoc corrected SpO2 equation rather than raw device output and contains repeated measurements in saturation strata.	Methods; p. 261	B
BENOIT_1 997	Benoit et al., 1997	D1	D1Q2	Were exclusions and missing participants unlikely to depend on the index or reference result?	Yes	The archived report-level evidence supports the low-risk condition.; Ten healthy men; radial arterial OSM3 co-oximetry; reported agreement uses a post-hoc corrected SpO2 equation rather than raw device output and contains repeated measurements in saturation strata.	Methods; p. 261	B
BENOIT_1 997	Benoit et al., 1997	D1	D1Q3	Was the analyzed comparison not restricted to a selectively chosen validation subgroup?	Yes	The archived report-level evidence supports the low-risk condition.; Ten healthy men; radial arterial OSM3 co-oximetry; reported agreement uses a post-hoc corrected SpO2 equation rather than raw device output and contains repeated measurements in saturation strata.	Methods; p. 261	B/R
BENOIT_1 997	Benoit et al., 1997	D2	D2Q1	Were device, site, configuration/averaging, and changes sufficiently reported and stable?	Yes	The archived report-level evidence supports the low-risk condition.; Ten healthy men; radial arterial OSM3 co-oximetry; reported agreement uses a post-hoc corrected SpO2 equation rather than raw device output and contains repeated measurements in saturation strata.	Methods; p. 261	B/R
BENOIT_1 997	Benoit et al., 1997	D2	D2Q2	Was the analyzed index the raw SpO2 rather than a post hoc corrected or secondary index?	No	The archived report-level evidence documents a failure or contradiction for this condition.; Ten healthy men; radial arterial OSM3 co-oximetry; reported agreement uses a post-hoc corrected SpO2 equation rather than raw device output and contains repeated measurements in saturation strata.	Methods; p. 261	B/R
BENOIT_1 997	Benoit et al., 1997	D2	D2Q3	Were signal-quality handling, failed readings, and index selection handled without evident result-dependent selection?	No	The archived report-level evidence documents a failure or contradiction for this condition.; Ten healthy men; radial arterial OSM3 co-oximetry; reported agreement uses a post-hoc corrected SpO2 equation rather than raw device output and	Methods; p. 261	B

Study ID	Author-year	Domain	Item	Signaling question	Final response	Rationale and study evidence	Evidence location	Item type
						contains repeated measurements in saturation strata.		
BENOIT_1997	Benoit et al., 1997	D3	D3Q1	Was SaO2 obtained from a direct arterial sample using multiwavelength co-oximetry?	Yes	The archived report-level evidence supports the low-risk condition.; Ten healthy men; radial arterial OSM3 co-oximetry; reported agreement uses a post-hoc corrected SpO2 equation rather than raw device output and contains repeated measurements in saturation strata.	Methods; p. 261	E/R
BENOIT_1997	Benoit et al., 1997	D3	D3Q2	Was the functional/fractional saturation target clear and internally noncontradictory?	Yes	The archived report-level evidence supports the low-risk condition.; Ten healthy men; radial arterial OSM3 co-oximetry; reported agreement uses a post-hoc corrected SpO2 equation rather than raw device output and contains repeated measurements in saturation strata.	Methods; p. 261	B/R
BENOIT_1997	Benoit et al., 1997	D3	D3Q3	Was the reference result generated independently of the index result?	Yes	The archived report-level evidence supports the low-risk condition.; Ten healthy men; radial arterial OSM3 co-oximetry; reported agreement uses a post-hoc corrected SpO2 equation rather than raw device output and contains repeated measurements in saturation strata.	Methods; p. 261	B
BENOIT_1997	Benoit et al., 1997	D4	D4Q1	Were SpO2 and SaO2 paired contemporaneously during exercise or within two minutes after cessation?	Yes	The archived report-level evidence supports the low-risk condition.; Ten healthy men; radial arterial OSM3 co-oximetry; reported agreement uses a post-hoc corrected SpO2 equation rather than raw device output and contains repeated measurements in saturation strata.	Methods; p. 261	B/R
BENOIT_1997	Benoit et al., 1997	D4	D4Q2	Were eligible pairs included representatively and missing pairs or failed readings explained?	Unclear	The archived report-level evidence does not establish this condition clearly.; Ten healthy men; radial arterial OSM3 co-oximetry; reported agreement uses a post-hoc corrected SpO2 equation rather than raw device output and contains repeated measurements in saturation strata.	Methods; p. 261	B
BENOIT_1997	Benoit et al., 1997	D4	D4Q3	Did the analysis account for repeated measures, multiple devices/sites, and mixed periods?	No	The archived report-level evidence documents a failure or contradiction for this condition.; Ten healthy men; radial arterial OSM3 co-oximetry; reported agreement uses a post-hoc corrected SpO2 equation rather than raw device output and contains repeated measurements in saturation strata.	Methods; p. 261	B

Study ID	Author-year	Domain	Item	Signaling question	Final response	Rationale and study evidence	Evidence location	Item type
MCGOVERN_1996	McGovern et al., 1996	D1	D1Q1	Was enrollment consecutive/random or based on a clearly defined cohort without case-control enrichment?	Unclear	The archived report-level evidence does not establish this condition clearly.; Eight severe-COPD participants; fixed Ohmeda finger device with signal-quality review; IL482 co-oximetry; 49 exercise pairs from eight participants with no recoverable clustering for the reported precision.	Methods and Results; p. 1153	B
MCGOVERN_1996	McGovern et al., 1996	D1	D1Q2	Were exclusions and missing participants unlikely to depend on the index or reference result?	Yes	The archived report-level evidence supports the low-risk condition.; Eight severe-COPD participants; fixed Ohmeda finger device with signal-quality review; IL482 co-oximetry; 49 exercise pairs from eight participants with no recoverable clustering for the reported precision.	Methods and Results; p. 1153	B
MCGOVERN_1996	McGovern et al., 1996	D1	D1Q3	Was the analyzed comparison not restricted to a selectively chosen validation subgroup?	Yes	The archived report-level evidence supports the low-risk condition.; Eight severe-COPD participants; fixed Ohmeda finger device with signal-quality review; IL482 co-oximetry; 49 exercise pairs from eight participants with no recoverable clustering for the reported precision.	Methods and Results; p. 1153	B/R
MCGOVERN_1996	McGovern et al., 1996	D2	D2Q1	Were device, site, configuration/averaging, and changes sufficiently reported and stable?	Yes	The archived report-level evidence supports the low-risk condition.; Eight severe-COPD participants; fixed Ohmeda finger device with signal-quality review; IL482 co-oximetry; 49 exercise pairs from eight participants with no recoverable clustering for the reported precision.	Methods and Results; p. 1153	B/R
MCGOVERN_1996	McGovern et al., 1996	D2	D2Q2	Was the analyzed index the raw SpO2 rather than a post hoc corrected or secondary index?	Yes	The archived report-level evidence supports the low-risk condition.; Eight severe-COPD participants; fixed Ohmeda finger device with signal-quality review; IL482 co-oximetry; 49 exercise pairs from eight participants with no recoverable clustering for the reported precision.	Methods and Results; p. 1153	B/R
MCGOVERN_1996	McGovern et al., 1996	D2	D2Q3	Were signal-quality handling, failed readings, and index selection handled without evident result-dependent selection?	Yes	The archived report-level evidence supports the low-risk condition.; Eight severe-COPD participants; fixed Ohmeda finger device with signal-quality review; IL482 co-oximetry; 49 exercise pairs from eight participants with no recoverable clustering for the reported precision.	Methods and Results; p. 1153	B
MCGOVERN_1996	McGovern et al., 1996	D3	D3Q1	Was SaO2 obtained from a direct arterial sample using multiwavelength co-oximetry?	Yes	The archived report-level evidence supports the low-risk condition.; Eight severe-COPD participants; fixed Ohmeda finger device with signal-quality review; IL482 co-oximetry; 49 exercise pairs from eight participants with no recoverable clustering for the reported precision.	Methods and Results; p. 1153	E/R

Study ID	Author-year	Domain	Item	Signaling question	Final response	Rationale and study evidence	Evidence location	Item type
MCGOVERN_1996	McGovern et al., 1996	D3	D3Q2	Was the functional/fractional saturation target clear and internally noncontradictory?	Yes	The archived report-level evidence supports the low-risk condition.; Eight severe-COPD participants; fixed Ohmeda finger device with signal-quality review; IL482 co-oximetry; 49 exercise pairs from eight participants with no recoverable clustering for the reported precision.	Methods and Results; p. 1153	B/R
MCGOVERN_1996	McGovern et al., 1996	D3	D3Q3	Was the reference result generated independently of the index result?	Yes	The archived report-level evidence supports the low-risk condition.; Eight severe-COPD participants; fixed Ohmeda finger device with signal-quality review; IL482 co-oximetry; 49 exercise pairs from eight participants with no recoverable clustering for the reported precision.	Methods and Results; p. 1153	B
MCGOVERN_1996	McGovern et al., 1996	D4	D4Q1	Were SpO2 and SaO2 paired contemporaneously during exercise or within two minutes after cessation?	Yes	The archived report-level evidence supports the low-risk condition.; Eight severe-COPD participants; fixed Ohmeda finger device with signal-quality review; IL482 co-oximetry; 49 exercise pairs from eight participants with no recoverable clustering for the reported precision.	Methods and Results; p. 1153	B/R
MCGOVERN_1996	McGovern et al., 1996	D4	D4Q2	Were eligible pairs included representatively and missing pairs or failed readings explained?	Yes	The archived report-level evidence supports the low-risk condition.; Eight severe-COPD participants; fixed Ohmeda finger device with signal-quality review; IL482 co-oximetry; 49 exercise pairs from eight participants with no recoverable clustering for the reported precision.	Methods and Results; p. 1153	B
MCGOVERN_1996	McGovern et al., 1996	D4	D4Q3	Did the analysis account for repeated measures, multiple devices/sites, and mixed periods?	No	The archived report-level evidence documents a failure or contradiction for this condition.; Eight severe-COPD participants; fixed Ohmeda finger device with signal-quality review; IL482 co-oximetry; 49 exercise pairs from eight participants with no recoverable clustering for the reported precision.	Methods and Results; p. 1153	B
CARONE_1997	Carone et al., 1997	D1	D1Q1	Was enrollment consecutive/random or based on a clearly defined cohort without case-control enrichment?	Unclear	The archived report-level evidence does not establish this condition clearly.; COPD patients receiving long-term oxygen therapy; fixed Biox ear device and IL282 co-oximetry; agreement set combines baseline, repeated walking time points, oxygen titration, and repeated tests; number of agreement pairs is not reported.	Methods and Results; p. 448	B
CARONE_1997	Carone et al., 1997	D1	D1Q2	Were exclusions and missing participants unlikely to depend on the index or reference result?	Yes	The archived report-level evidence supports the low-risk condition.; COPD patients receiving long-term oxygen therapy; fixed Biox ear device and IL282 co-oximetry; agreement set combines baseline, repeated walking time points, oxygen	Methods and Results; p. 448	B

Study ID	Author-year	Domain	Item	Signaling question	Final response	Rationale and study evidence	Evidence location	Item type
						titration, and repeated tests; number of agreement pairs is not reported.		
CARONE_ 1997	Carone et al., 1997	D1	D1Q3	Was the analyzed comparison not restricted to a selectively chosen validation subgroup?	Yes	The archived report-level evidence supports the low-risk condition.; COPD patients receiving long-term oxygen therapy; fixed Biox ear device and IL282 co-oximetry; agreement set combines baseline, repeated walking time points, oxygen titration, and repeated tests; number of agreement pairs is not reported.	Methods and Results; p. 448	B/R
CARONE_ 1997	Carone et al., 1997	D2	D2Q1	Were device, site, configuration/averaging, and changes sufficiently reported and stable?	Yes	The archived report-level evidence supports the low-risk condition.; COPD patients receiving long-term oxygen therapy; fixed Biox ear device and IL282 co-oximetry; agreement set combines baseline, repeated walking time points, oxygen titration, and repeated tests; number of agreement pairs is not reported.	Methods and Results; p. 448	B/R
CARONE_ 1997	Carone et al., 1997	D2	D2Q2	Was the analyzed index the raw SpO2 rather than a post hoc corrected or secondary index?	Yes	The archived report-level evidence supports the low-risk condition.; COPD patients receiving long-term oxygen therapy; fixed Biox ear device and IL282 co-oximetry; agreement set combines baseline, repeated walking time points, oxygen titration, and repeated tests; number of agreement pairs is not reported.	Methods and Results; p. 448	B/R
CARONE_ 1997	Carone et al., 1997	D2	D2Q3	Were signal-quality handling, failed readings, and index selection handled without evident result-dependent selection?	Yes	The archived report-level evidence supports the low-risk condition.; COPD patients receiving long-term oxygen therapy; fixed Biox ear device and IL282 co-oximetry; agreement set combines baseline, repeated walking time points, oxygen titration, and repeated tests; number of agreement pairs is not reported.	Methods and Results; p. 448	B
CARONE_ 1997	Carone et al., 1997	D3	D3Q1	Was SaO2 obtained from a direct arterial sample using multiwavelength co-oximetry?	Yes	The archived report-level evidence supports the low-risk condition.; COPD patients receiving long-term oxygen therapy; fixed Biox ear device and IL282 co-oximetry; agreement set combines baseline, repeated walking time points, oxygen titration, and repeated tests; number of agreement pairs is not reported.	Methods and Results; p. 448	E/R
CARONE_ 1997	Carone et al., 1997	D3	D3Q2	Was the functional/fractional saturation target clear and internally noncontradictory?	Yes	The archived report-level evidence supports the low-risk condition.; COPD patients receiving long-term oxygen therapy; fixed Biox ear device and IL282 co-oximetry; agreement set combines baseline, repeated walking time points, oxygen	Methods and Results; p. 448	B/R

Study ID	Author-year	Domain	Item	Signaling question	Final response	Rationale and study evidence	Evidence location	Item type
						titration, and repeated tests; number of agreement pairs is not reported.		
CARONE_ 1997	Carone et al., 1997	D3	D3Q3	Was the reference result generated independently of the index result?	Yes	The archived report-level evidence supports the low-risk condition.; COPD patients receiving long-term oxygen therapy; fixed Biox ear device and IL282 co-oximetry; agreement set combines baseline, repeated walking time points, oxygen titration, and repeated tests; number of agreement pairs is not reported.	Methods and Results; p. 448	B
CARONE_ 1997	Carone et al., 1997	D4	D4Q1	Were SpO2 and SaO2 paired contemporaneously during exercise or within two minutes after cessation?	Yes	The archived report-level evidence supports the low-risk condition.; COPD patients receiving long-term oxygen therapy; fixed Biox ear device and IL282 co-oximetry; agreement set combines baseline, repeated walking time points, oxygen titration, and repeated tests; number of agreement pairs is not reported.	Methods and Results; p. 448	B/R
CARONE_ 1997	Carone et al., 1997	D4	D4Q2	Were eligible pairs included representatively and missing pairs or failed readings explained?	Unclear	The archived report-level evidence does not establish this condition clearly.; COPD patients receiving long-term oxygen therapy; fixed Biox ear device and IL282 co-oximetry; agreement set combines baseline, repeated walking time points, oxygen titration, and repeated tests; number of agreement pairs is not reported.	Methods and Results; p. 448	B
CARONE_ 1997	Carone et al., 1997	D4	D4Q3	Did the analysis account for repeated measures, multiple devices/sites, and mixed periods?	No	The archived report-level evidence documents a failure or contradiction for this condition.; COPD patients receiving long-term oxygen therapy; fixed Biox ear device and IL282 co-oximetry; agreement set combines baseline, repeated walking time points, oxygen titration, and repeated tests; number of agreement pairs is not reported.	Methods and Results; p. 448	B
MARTIN_ 1992	Martin et al., 1992	D1	D1Q1	Was enrollment consecutive/random or based on a clearly defined cohort without case-control enrichment?	Unclear	The archived report-level evidence does not establish this condition clearly.; Eleven elite male cyclists; specified simultaneous ear/finger devices; IL282 directly measured fractional oxyhaemoglobin; 232 repeated exercise observations per probe and three correlated device-site outcomes.	Methods and Results; p. 456	B
MARTIN_ 1992	Martin et al., 1992	D1	D1Q2	Were exclusions and missing participants unlikely to depend on the index or reference result?	Yes	The archived report-level evidence supports the low-risk condition.; Eleven elite male cyclists; specified simultaneous ear/finger devices; IL282 directly measured fractional oxyhaemoglobin; 232 repeated exercise observations per probe and three correlated device-site outcomes.	Methods and Results; p. 456	B

Study ID	Author-year	Domain	Item	Signaling question	Final response	Rationale and study evidence	Evidence location	Item type
MARTIN_ 1992	Martin et al., 1992	D1	D1Q3	Was the analyzed comparison not restricted to a selectively chosen validation subgroup?	Yes	The archived report-level evidence supports the low-risk condition.; Eleven elite male cyclists; specified simultaneous ear/finger devices; IL282 directly measured fractional oxyhaemoglobin; 232 repeated exercise observations per probe and three correlated device-site outcomes.	Methods and Results; p. 456	B/R
MARTIN_ 1992	Martin et al., 1992	D2	D2Q1	Were device, site, configuration/averaging, and changes sufficiently reported and stable?	Yes	The archived report-level evidence supports the low-risk condition.; Eleven elite male cyclists; specified simultaneous ear/finger devices; IL282 directly measured fractional oxyhaemoglobin; 232 repeated exercise observations per probe and three correlated device-site outcomes.	Methods and Results; p. 456	B/R
MARTIN_ 1992	Martin et al., 1992	D2	D2Q2	Was the analyzed index the raw SpO2 rather than a post hoc corrected or secondary index?	Yes	The archived report-level evidence supports the low-risk condition.; Eleven elite male cyclists; specified simultaneous ear/finger devices; IL282 directly measured fractional oxyhaemoglobin; 232 repeated exercise observations per probe and three correlated device-site outcomes.	Methods and Results; p. 456	B/R
MARTIN_ 1992	Martin et al., 1992	D2	D2Q3	Were signal-quality handling, failed readings, and index selection handled without evident result-dependent selection?	Yes	The archived report-level evidence supports the low-risk condition.; Eleven elite male cyclists; specified simultaneous ear/finger devices; IL282 directly measured fractional oxyhaemoglobin; 232 repeated exercise observations per probe and three correlated device-site outcomes.	Methods and Results; p. 456	B
MARTIN_ 1992	Martin et al., 1992	D3	D3Q1	Was SaO2 obtained from a direct arterial sample using multiwavelength co-oximetry?	Yes	The archived report-level evidence supports the low-risk condition.; Eleven elite male cyclists; specified simultaneous ear/finger devices; IL282 directly measured fractional oxyhaemoglobin; 232 repeated exercise observations per probe and three correlated device-site outcomes.	Methods and Results; p. 456	E/R
MARTIN_ 1992	Martin et al., 1992	D3	D3Q2	Was the functional/fractional saturation target clear and internally noncontradictory?	Yes	The archived report-level evidence supports the low-risk condition.; Eleven elite male cyclists; specified simultaneous ear/finger devices; IL282 directly measured fractional oxyhaemoglobin; 232 repeated exercise observations per probe and three correlated device-site outcomes.	Methods and Results; p. 456	B/R
MARTIN_ 1992	Martin et al., 1992	D3	D3Q3	Was the reference result generated independently of the index result?	Yes	The archived report-level evidence supports the low-risk condition.; Eleven elite male cyclists; specified simultaneous ear/finger devices; IL282 directly measured fractional oxyhaemoglobin; 232 repeated exercise observations per probe and three correlated device-site outcomes.	Methods and Results; p. 456	B

Study ID	Author-year	Domain	Item	Signaling question	Final response	Rationale and study evidence	Evidence location	Item type
MARTIN_ 1992	Martin et al., 1992	D4	D4Q1	Were SpO2 and SaO2 paired contemporaneously during exercise or within two minutes after cessation?	Yes	The archived report-level evidence supports the low-risk condition.; Eleven elite male cyclists; specified simultaneous ear/finger devices; IL282 directly measured fractional oxyhaemoglobin; 232 repeated exercise observations per probe and three correlated device-site outcomes.	Methods and Results; p. 456	B/R
MARTIN_ 1992	Martin et al., 1992	D4	D4Q2	Were eligible pairs included representatively and missing pairs or failed readings explained?	Yes	The archived report-level evidence supports the low-risk condition.; Eleven elite male cyclists; specified simultaneous ear/finger devices; IL282 directly measured fractional oxyhaemoglobin; 232 repeated exercise observations per probe and three correlated device-site outcomes.	Methods and Results; p. 456	B
MARTIN_ 1992	Martin et al., 1992	D4	D4Q3	Did the analysis account for repeated measures, multiple devices/sites, and mixed periods?	No	The archived report-level evidence documents a failure or contradiction for this condition.; Eleven elite male cyclists; specified simultaneous ear/finger devices; IL282 directly measured fractional oxyhaemoglobin; 232 repeated exercise observations per probe and three correlated device-site outcomes.	Methods and Results; p. 456	B
BARTHEL EMY_1990	Barthélémy et al., 1990	D1	D1Q1	Was enrollment consecutive/random or based on a clearly defined cohort without case-control enrichment?	Unclear	The archived report-level evidence does not establish this condition clearly.; Fifty-seven adults; eight finger-device comparisons; OSM3 functional co-oximetry; device-specific sample counts are repeated observations rather than independent participants.	Methods and Results; p. 535	B
BARTHEL EMY_1990	Barthélémy et al., 1990	D1	D1Q2	Were exclusions and missing participants unlikely to depend on the index or reference result?	Yes	The archived report-level evidence supports the low-risk condition.; Fifty-seven adults; eight finger-device comparisons; OSM3 functional co-oximetry; device-specific sample counts are repeated observations rather than independent participants.	Methods and Results; p. 535	B
BARTHEL EMY_1990	Barthélémy et al., 1990	D1	D1Q3	Was the analyzed comparison not restricted to a selectively chosen validation subgroup?	Yes	The archived report-level evidence supports the low-risk condition.; Fifty-seven adults; eight finger-device comparisons; OSM3 functional co-oximetry; device-specific sample counts are repeated observations rather than independent participants.	Methods and Results; p. 535	B/R
BARTHEL EMY_1990	Barthélémy et al., 1990	D2	D2Q1	Were device, site, configuration/averaging, and changes sufficiently reported and stable?	Unclear	The archived report-level evidence does not establish this condition clearly.; Fifty-seven adults; eight finger-device comparisons; OSM3 functional co-oximetry; device-specific sample counts are repeated observations rather than independent participants.	Methods and Results; p. 535	B/R

**Study ID**	**Author-year**	**Domain**	**Item**	**Signaling question**	**Final response**	**Rationale and study evidence**	**Evidence location**	Item type
BARTHEL EMY_1990	Barthélémy et al., 1990	D2	D2Q2	Was the analyzed index the raw SpO2 rather than a post hoc corrected or secondary index?	Yes	The archived report-level evidence supports the low-risk condition.; Fifty-seven adults; eight finger-device comparisons; OSM3 functional co-oximetry; device-specific sample counts are repeated observations rather than independent participants.	Methods and Results; p. 535	B/R
BARTHEL EMY_1990	Barthélémy et al., 1990	D2	D2Q3	Were signal-quality handling, failed readings, and index selection handled without evident result-dependent selection?	Unclear	The archived report-level evidence does not establish this condition clearly.; Fifty-seven adults; eight finger-device comparisons; OSM3 functional co-oximetry; device-specific sample counts are repeated observations rather than independent participants.	Methods and Results; p. 535	B
BARTHEL EMY_1990	Barthélémy et al., 1990	D3	D3Q1	Was SaO2 obtained from a direct arterial sample using multiwavelength co-oximetry?	Yes	The archived report-level evidence supports the low-risk condition.; Fifty-seven adults; eight finger-device comparisons; OSM3 functional co-oximetry; device-specific sample counts are repeated observations rather than independent participants.	Methods and Results; p. 535	E/R
BARTHEL EMY_1990	Barthélémy et al., 1990	D3	D3Q2	Was the functional/fractional saturation target clear and internally noncontradictory?	Yes	The archived report-level evidence supports the low-risk condition.; Fifty-seven adults; eight finger-device comparisons; OSM3 functional co-oximetry; device-specific sample counts are repeated observations rather than independent participants.	Methods and Results; p. 535	B/R
BARTHEL EMY_1990	Barthélémy et al., 1990	D3	D3Q3	Was the reference result generated independently of the index result?	Yes	The archived report-level evidence supports the low-risk condition.; Fifty-seven adults; eight finger-device comparisons; OSM3 functional co-oximetry; device-specific sample counts are repeated observations rather than independent participants.	Methods and Results; p. 535	B
BARTHEL EMY_1990	Barthélémy et al., 1990	D4	D4Q1	Were SpO2 and SaO2 paired contemporaneously during exercise or within two minutes after cessation?	Yes	The archived report-level evidence supports the low-risk condition.; Fifty-seven adults; eight finger-device comparisons; OSM3 functional co-oximetry; device-specific sample counts are repeated observations rather than independent participants.	Methods and Results; p. 535	B/R
BARTHEL EMY_1990	Barthélémy et al., 1990	D4	D4Q2	Were eligible pairs included representatively and missing pairs or failed readings explained?	Unclear	The archived report-level evidence does not establish this condition clearly.; Fifty-seven adults; eight finger-device comparisons; OSM3 functional co-oximetry; device-specific sample counts are repeated observations rather than independent participants.	Methods and Results; p. 535	B

Study ID	Author-year	Domain	Item	Signaling question	Final response	Rationale and study evidence	Evidence location	Item type
BARTHELEMY_1990	Barthélémy et al., 1990	D4	D4Q3	Did the analysis account for repeated measures, multiple devices/sites, and mixed periods?	No	The archived report-level evidence documents a failure or contradiction for this condition.; Fifty-seven adults; eight finger-device comparisons; OSM3 functional co-oximetry; device-specific sample counts are repeated observations rather than independent participants.	Methods and Results; p. 535	B
POWERS_1989	Powers et al., 1989	D1	D1Q1	Was enrollment consecutive/random or based on a clearly defined cohort without case-control enrichment?	Unclear	The archived report-level evidence does not establish this condition clearly.; Ten healthy men; three simultaneous device/site configurations; synchronized radial samples and IL282 direct fractional HbO2; highest-work result gives one pair per participant for each correlated configuration.	Methods; Table 1; p. 301	B
POWERS_1989	Powers et al., 1989	D1	D1Q2	Were exclusions and missing participants unlikely to depend on the index or reference result?	Yes	The archived report-level evidence supports the low-risk condition.; Ten healthy men; three simultaneous device/site configurations; synchronized radial samples and IL282 direct fractional HbO2; highest-work result gives one pair per participant for each correlated configuration.	Methods; Table 1; p. 301	B
POWERS_1989	Powers et al., 1989	D1	D1Q3	Was the analyzed comparison not restricted to a selectively chosen validation subgroup?	Yes	The archived report-level evidence supports the low-risk condition.; Ten healthy men; three simultaneous device/site configurations; synchronized radial samples and IL282 direct fractional HbO2; highest-work result gives one pair per participant for each correlated configuration.	Methods; Table 1; p. 301	B/R
POWERS_1989	Powers et al., 1989	D2	D2Q1	Were device, site, configuration/averaging, and changes sufficiently reported and stable?	Yes	The archived report-level evidence supports the low-risk condition.; Ten healthy men; three simultaneous device/site configurations; synchronized radial samples and IL282 direct fractional HbO2; highest-work result gives one pair per participant for each correlated configuration.	Methods; Table 1; p. 301	B/R
POWERS_1989	Powers et al., 1989	D2	D2Q2	Was the analyzed index the raw SpO2 rather than a post hoc corrected or secondary index?	Yes	The archived report-level evidence supports the low-risk condition.; Ten healthy men; three simultaneous device/site configurations; synchronized radial samples and IL282 direct fractional HbO2; highest-work result gives one pair per participant for each correlated configuration.	Methods; Table 1; p. 301	B/R

Study ID	Author-year	Domain	Item	Signaling question	Final response	Rationale and study evidence	Evidence location	Item type
POWERS_ 1989	Powers et al., 1989	D2	D2Q3	Were signal-quality handling, failed readings, and index selection handled without evident result-dependent selection?	Yes	The archived report-level evidence supports the low-risk condition.; Ten healthy men; three simultaneous device/site configurations; synchronized radial samples and IL282 direct fractional HbO2; highest-work result gives one pair per participant for each correlated configuration.	Methods; Table 1; p. 301	B
POWERS_ 1989	Powers et al., 1989	D3	D3Q1	Was SaO2 obtained from a direct arterial sample using multiwavelength co-oximetry?	Yes	The archived report-level evidence supports the low-risk condition.; Ten healthy men; three simultaneous device/site configurations; synchronized radial samples and IL282 direct fractional HbO2; highest-work result gives one pair per participant for each correlated configuration.	Methods; Table 1; p. 301	E/R
POWERS_ 1989	Powers et al., 1989	D3	D3Q2	Was the functional/fractional saturation target clear and internally noncontradictory?	Yes	The archived report-level evidence supports the low-risk condition.; Ten healthy men; three simultaneous device/site configurations; synchronized radial samples and IL282 direct fractional HbO2; highest-work result gives one pair per participant for each correlated configuration.	Methods; Table 1; p. 301	B/R
POWERS_ 1989	Powers et al., 1989	D3	D3Q3	Was the reference result generated independently of the index result?	Yes	The archived report-level evidence supports the low-risk condition.; Ten healthy men; three simultaneous device/site configurations; synchronized radial samples and IL282 direct fractional HbO2; highest-work result gives one pair per participant for each correlated configuration.	Methods; Table 1; p. 301	B
POWERS_ 1989	Powers et al., 1989	D4	D4Q1	Were SpO2 and SaO2 paired contemporaneously during exercise or within two minutes after cessation?	Yes	The archived report-level evidence supports the low-risk condition.; Ten healthy men; three simultaneous device/site configurations; synchronized radial samples and IL282 direct fractional HbO2; highest-work result gives one pair per participant for each correlated configuration.	Methods; Table 1; p. 301	B/R
POWERS_ 1989	Powers et al., 1989	D4	D4Q2	Were eligible pairs included representatively and missing pairs or failed readings explained?	Yes	The archived report-level evidence supports the low-risk condition.; Ten healthy men; three simultaneous device/site configurations; synchronized radial samples and IL282 direct fractional HbO2; highest-work result gives one pair per participant for each correlated configuration.	Methods; Table 1; p. 301	B

Study ID	Author-year	Domain	Item	Signaling question	Final response	Rationale and study evidence	Evidence location	Item type
POWERS_1989	Powers et al., 1989	D4	D4Q3	Did the analysis account for repeated measures, multiple devices/sites, and mixed periods?	Unclear	The archived report-level evidence does not establish this condition clearly.; Ten healthy men; three simultaneous device/site configurations; synchronized radial samples and IL282 direct fractional HbO2; highest-work result gives one pair per participant for each correlated configuration.	Methods; Table 1; p. 301	B
RIES_1985	Ries et al., 1985	D1	D1Q1	Was enrollment consecutive/random or based on a clearly defined cohort without case-control enrichment?	Unclear	The archived report-level evidence does not establish this condition clearly.; Pulmonary-laboratory patients across three device phases; radial samples within five seconds and IL282 direct co-oximetry; repeated exercise samples overlap across phases and reported SEE is not paired-difference SD.	Methods; Tables 1-3; pp. 686-688	B
RIES_1985	Ries et al., 1985	D1	D1Q2	Were exclusions and missing participants unlikely to depend on the index or reference result?	Yes	The archived report-level evidence supports the low-risk condition.; Pulmonary-laboratory patients across three device phases; radial samples within five seconds and IL282 direct co-oximetry; repeated exercise samples overlap across phases and reported SEE is not paired-difference SD.	Methods; Tables 1-3; pp. 686-688	B
RIES_1985	Ries et al., 1985	D1	D1Q3	Was the analyzed comparison not restricted to a selectively chosen validation subgroup?	Yes	The archived report-level evidence supports the low-risk condition.; Pulmonary-laboratory patients across three device phases; radial samples within five seconds and IL282 direct co-oximetry; repeated exercise samples overlap across phases and reported SEE is not paired-difference SD.	Methods; Tables 1-3; pp. 686-688	B/R
RIES_1985	Ries et al., 1985	D2	D2Q1	Were device, site, configuration/averaging, and changes sufficiently reported and stable?	Unclear	The archived report-level evidence does not establish this condition clearly.; Pulmonary-laboratory patients across three device phases; radial samples within five seconds and IL282 direct co-oximetry; repeated exercise samples overlap across phases and reported SEE is not paired-difference SD.	Methods; Tables 1-3; pp. 686-688	B/R
RIES_1985	Ries et al., 1985	D2	D2Q2	Was the analyzed index the raw SpO2 rather than a post hoc corrected or secondary index?	Yes	The archived report-level evidence supports the low-risk condition.; Pulmonary-laboratory patients across three device phases; radial samples within five seconds and IL282 direct co-oximetry; repeated exercise samples overlap across phases and reported SEE is not paired-difference SD.	Methods; Tables 1-3; pp. 686-688	B/R
RIES_1985	Ries et al., 1985	D2	D2Q3	Were signal-quality handling, failed readings, and index selection handled without evident result-dependent selection?	Yes	The archived report-level evidence supports the low-risk condition.; Pulmonary-laboratory patients across three device phases; radial samples within five seconds and IL282 direct co-oximetry;	Methods; Tables 1-3; pp. 686-688	B

Study ID	Author-year	Domain	Item	Signaling question	Final response	Rationale and study evidence	Evidence location	Item type
						repeated exercise samples overlap across phases and reported SEE is not paired-difference SD.		
RIES_1985	Ries et al., 1985	D3	D3Q1	Was SaO2 obtained from a direct arterial sample using multiwavelength co-oximetry?	Yes	The archived report-level evidence supports the low-risk condition.; Pulmonary-laboratory patients across three device phases; radial samples within five seconds and IL282 direct co-oximetry; repeated exercise samples overlap across phases and reported SEE is not paired-difference SD.	Methods; Tables 1-3; pp. 686-688	E/R
RIES_1985	Ries et al., 1985	D3	D3Q2	Was the functional/fractional saturation target clear and internally noncontradictory?	Yes	The archived report-level evidence supports the low-risk condition.; Pulmonary-laboratory patients across three device phases; radial samples within five seconds and IL282 direct co-oximetry; repeated exercise samples overlap across phases and reported SEE is not paired-difference SD.	Methods; Tables 1-3; pp. 686-688	B/R
RIES_1985	Ries et al., 1985	D3	D3Q3	Was the reference result generated independently of the index result?	Yes	The archived report-level evidence supports the low-risk condition.; Pulmonary-laboratory patients across three device phases; radial samples within five seconds and IL282 direct co-oximetry; repeated exercise samples overlap across phases and reported SEE is not paired-difference SD.	Methods; Tables 1-3; pp. 686-688	B
RIES_1985	Ries et al., 1985	D4	D4Q1	Were SpO2 and SaO2 paired contemporaneously during exercise or within two minutes after cessation?	Yes	The archived report-level evidence supports the low-risk condition.; Pulmonary-laboratory patients across three device phases; radial samples within five seconds and IL282 direct co-oximetry; repeated exercise samples overlap across phases and reported SEE is not paired-difference SD.	Methods; Tables 1-3; pp. 686-688	B/R
RIES_1985	Ries et al., 1985	D4	D4Q2	Were eligible pairs included representatively and missing pairs or failed readings explained?	Unclear	The archived report-level evidence does not establish this condition clearly.; Pulmonary-laboratory patients across three device phases; radial samples within five seconds and IL282 direct co-oximetry; repeated exercise samples overlap across phases and reported SEE is not paired-difference SD.	Methods; Tables 1-3; pp. 686-688	B
RIES_1985	Ries et al., 1985	D4	D4Q3	Did the analysis account for repeated measures, multiple devices/sites, and mixed periods?	No	The archived report-level evidence documents a failure or contradiction for this condition.; Pulmonary-laboratory patients across three device phases; radial samples within five seconds and IL282 direct co-oximetry; repeated exercise samples overlap across phases and reported SEE is not paired-difference SD.	Methods; Tables 1-3; pp. 686-688	B

Study ID	Author-year	Domain	Item	Signaling question	Final response	Rationale and study evidence	Evidence location	Item type
RICE_1999	Rice et al., 1999	D1	D1Q1	Was enrollment consecutive/random or based on a clearly defined cohort without case-control enrichment?	Unclear	The archived report-level evidence does not establish this condition clearly.; Fifteen competitive male cyclists; fixed ear device and synchronized radial sampling; the report names ABL520 for SaO2 but provides limited direct-method detail; comparison is graphical across rest and repeated exercise stages.	Methods; Fig. 4	B
RICE_1999	Rice et al., 1999	D1	D1Q2	Were exclusions and missing participants unlikely to depend on the index or reference result?	Yes	The archived report-level evidence supports the low-risk condition.; Fifteen competitive male cyclists; fixed ear device and synchronized radial sampling; the report names ABL520 for SaO2 but provides limited direct-method detail; comparison is graphical across rest and repeated exercise stages.	Methods; Fig. 4	B
RICE_1999	Rice et al., 1999	D1	D1Q3	Was the analyzed comparison not restricted to a selectively chosen validation subgroup?	Yes	The archived report-level evidence supports the low-risk condition.; Fifteen competitive male cyclists; fixed ear device and synchronized radial sampling; the report names ABL520 for SaO2 but provides limited direct-method detail; comparison is graphical across rest and repeated exercise stages.	Methods; Fig. 4	B/R
RICE_1999	Rice et al., 1999	D2	D2Q1	Were device, site, configuration/averaging, and changes sufficiently reported and stable?	Yes	The archived report-level evidence supports the low-risk condition.; Fifteen competitive male cyclists; fixed ear device and synchronized radial sampling; the report names ABL520 for SaO2 but provides limited direct-method detail; comparison is graphical across rest and repeated exercise stages.	Methods; Fig. 4	B/R
RICE_1999	Rice et al., 1999	D2	D2Q2	Was the analyzed index the raw SpO2 rather than a post hoc corrected or secondary index?	Yes	The archived report-level evidence supports the low-risk condition.; Fifteen competitive male cyclists; fixed ear device and synchronized radial sampling; the report names ABL520 for SaO2 but provides limited direct-method detail; comparison is graphical across rest and repeated exercise stages.	Methods; Fig. 4	B/R
RICE_1999	Rice et al., 1999	D2	D2Q3	Were signal-quality handling, failed readings, and index selection handled without evident result-dependent selection?	Yes	The archived report-level evidence supports the low-risk condition.; Fifteen competitive male cyclists; fixed ear device and synchronized radial sampling; the report names ABL520 for SaO2 but provides limited direct-method detail; comparison is graphical across rest and repeated exercise stages.	Methods; Fig. 4	B

Study ID	Author-year	Domain	Item	Signaling question	Final response	Rationale and study evidence	Evidence location	Item type
RICE_1999	Rice et al., 1999	D3	D3Q1	Was SaO2 obtained from a direct arterial sample using multiwavelength co-oximetry?	Yes	The archived report-level evidence supports the low-risk condition.; Fifteen competitive male cyclists; fixed ear device and synchronized radial sampling; the report names ABL520 for SaO2 but provides limited direct-method detail; comparison is graphical across rest and repeated exercise stages.	Methods; Fig. 4	E/R
RICE_1999	Rice et al., 1999	D3	D3Q2	Was the functional/fractional saturation target clear and internally noncontradictory?	Unclear	The archived report-level evidence does not establish this condition clearly.; Fifteen competitive male cyclists; fixed ear device and synchronized radial sampling; the report names ABL520 for SaO2 but provides limited direct-method detail; comparison is graphical across rest and repeated exercise stages.	Methods; Fig. 4	B/R
RICE_1999	Rice et al., 1999	D3	D3Q3	Was the reference result generated independently of the index result?	Yes	The archived report-level evidence supports the low-risk condition.; Fifteen competitive male cyclists; fixed ear device and synchronized radial sampling; the report names ABL520 for SaO2 but provides limited direct-method detail; comparison is graphical across rest and repeated exercise stages.	Methods; Fig. 4	B
RICE_1999	Rice et al., 1999	D4	D4Q1	Were SpO2 and SaO2 paired contemporaneously during exercise or within two minutes after cessation?	Yes	The archived report-level evidence supports the low-risk condition.; Fifteen competitive male cyclists; fixed ear device and synchronized radial sampling; the report names ABL520 for SaO2 but provides limited direct-method detail; comparison is graphical across rest and repeated exercise stages.	Methods; Fig. 4	B/R
RICE_1999	Rice et al., 1999	D4	D4Q2	Were eligible pairs included representatively and missing pairs or failed readings explained?	Unclear	The archived report-level evidence does not establish this condition clearly.; Fifteen competitive male cyclists; fixed ear device and synchronized radial sampling; the report names ABL520 for SaO2 but provides limited direct-method detail; comparison is graphical across rest and repeated exercise stages.	Methods; Fig. 4	B
RICE_1999	Rice et al., 1999	D4	D4Q3	Did the analysis account for repeated measures, multiple devices/sites, and mixed periods?	Unclear	The archived report-level evidence does not establish this condition clearly.; Fifteen competitive male cyclists; fixed ear device and synchronized radial sampling; the report names ABL520 for SaO2 but provides limited direct-method detail; comparison is graphical across rest and repeated exercise stages.	Methods; Fig. 4	B

Study ID	Author-year	Domain	Item	Signaling question	Final response	Rationale and study evidence	Evidence location	Item type
LUNDGREN_2001	Lundgren et al., 2001	D1	D1Q1	Was enrollment consecutive/random or based on a clearly defined cohort without case-control enrichment?	Unclear	The archived report-level evidence does not establish this condition clearly.; Retrospective beryllium sensitization/chronic beryllium disease cardiopulmonary exercise testing cohort; two pulse-oximeter generations and two co-oximeter platforms are combined across eras; one maximum-exercise pair per participant in the beryllium sensitization estimate.	Methods and Results; pp. 1702 and 1705	B
LUNDGREN_2001	Lundgren et al., 2001	D1	D1Q2	Were exclusions and missing participants unlikely to depend on the index or reference result?	Yes	The archived report-level evidence supports the low-risk condition.; Retrospective beryllium sensitization/chronic beryllium disease cardiopulmonary exercise testing cohort; two pulse-oximeter generations and two co-oximeter platforms are combined across eras; one maximum-exercise pair per participant in the beryllium sensitization estimate.	Methods and Results; pp. 1702 and 1705	B
LUNDGREN_2001	Lundgren et al., 2001	D1	D1Q3	Was the analyzed comparison not restricted to a selectively chosen validation subgroup?	Yes	The archived report-level evidence supports the low-risk condition.; Retrospective beryllium sensitization/chronic beryllium disease cardiopulmonary exercise testing cohort; two pulse-oximeter generations and two co-oximeter platforms are combined across eras; one maximum-exercise pair per participant in the beryllium sensitization estimate.	Methods and Results; pp. 1702 and 1705	B/R
LUNDGREN_2001	Lundgren et al., 2001	D2	D2Q1	Were device, site, configuration/averaging, and changes sufficiently reported and stable?	No	The archived report-level evidence documents a failure or contradiction for this condition.; Retrospective beryllium sensitization/chronic beryllium disease cardiopulmonary exercise testing cohort; two pulse-oximeter generations and two co-oximeter platforms are combined across eras; one maximum-exercise pair per participant in the beryllium sensitization estimate.	Methods and Results; pp. 1702 and 1705	B/R
LUNDGREN_2001	Lundgren et al., 2001	D2	D2Q2	Was the analyzed index the raw SpO2 rather than a post hoc corrected or secondary index?	Yes	The archived report-level evidence supports the low-risk condition.; Retrospective beryllium sensitization/chronic beryllium disease cardiopulmonary exercise testing cohort; two pulse-oximeter generations and two co-oximeter platforms are combined across eras; one maximum-exercise pair per participant in the beryllium sensitization estimate.	Methods and Results; pp. 1702 and 1705	B/R
LUNDGREN_2001	Lundgren et al., 2001	D2	D2Q3	Were signal-quality handling, failed readings, and index selection handled without evident result-dependent selection?	Unclear	The archived report-level evidence does not establish this condition clearly.; Retrospective beryllium sensitization/chronic beryllium disease cardiopulmonary exercise testing cohort; two	Methods and Results; pp. 1702 and 1705	B

Study ID	Author-year	Domain	Item	Signaling question	Final response	Rationale and study evidence	Evidence location	Item type
						pulse-oximeter generations and two co-oximeter platforms are combined across eras; one maximum-exercise pair per participant in the beryllium sensitization estimate.		
LUNDGREN_2001	Lundgren et al., 2001	D3	D3Q1	Was SaO2 obtained from a direct arterial sample using multiwavelength co-oximetry?	Yes	The archived report-level evidence supports the low-risk condition.; Retrospective beryllium sensitization/chronic beryllium disease cardiopulmonary exercise testing cohort; two pulse-oximeter generations and two co-oximeter platforms are combined across eras; one maximum-exercise pair per participant in the beryllium sensitization estimate.	Methods and Results; pp. 1702 and 1705	E/R
LUNDGREN_2001	Lundgren et al., 2001	D3	D3Q2	Was the functional/fractional saturation target clear and internally noncontradictory?	Unclear	Direct arterial co-oximetry is confirmed, but the functional/fractional saturation target is not explicitly defined; the review-defined D3Q2 condition is therefore unclear.; Retrospective beryllium sensitization/chronic beryllium disease cardiopulmonary exercise testing cohort; two pulse-oximeter generations and two co-oximeter platforms are combined across eras; one maximum-exercise pair per participant in the beryllium sensitization estimate. The report identifies two co-oximeter generations and calls the result SaO2, but does not define whether the reported saturation is functional or fractional.	Methods, Exercise Physiology Evaluation (article p. 1704)	B/R
LUNDGREN_2001	Lundgren et al., 2001	D3	D3Q3	Was the reference result generated independently of the index result?	Yes	The archived report-level evidence supports the low-risk condition.; Retrospective beryllium sensitization/chronic beryllium disease cardiopulmonary exercise testing cohort; two pulse-oximeter generations and two co-oximeter platforms are combined across eras; one maximum-exercise pair per participant in the beryllium sensitization estimate.	Methods and Results; pp. 1702 and 1705	B
LUNDGREN_2001	Lundgren et al., 2001	D4	D4Q1	Were SpO2 and SaO2 paired contemporaneously during exercise or within two minutes after cessation?	Yes	The archived report-level evidence supports the low-risk condition.; Retrospective beryllium sensitization/chronic beryllium disease cardiopulmonary exercise testing cohort; two pulse-oximeter generations and two co-oximeter platforms are combined across eras; one maximum-exercise pair per participant in the beryllium sensitization estimate.	Methods and Results; pp. 1702 and 1705	B/R

Study ID	Author-year	Domain	Item	Signaling question	Final response	Rationale and study evidence	Evidence location	Item type
LUNDGREN_2001	Lundgren et al., 2001	D4	D4Q2	Were eligible pairs included representatively and missing pairs or failed readings explained?	Yes	The archived report-level evidence supports the low-risk condition.; Retrospective beryllium sensitization/chronic beryllium disease cardiopulmonary exercise testing cohort; two pulse-oximeter generations and two co-oximeter platforms are combined across eras; one maximum-exercise pair per participant in the beryllium sensitization estimate.	Methods and Results; pp. 1702 and 1705	B
LUNDGREN_2001	Lundgren et al., 2001	D4	D4Q3	Did the analysis account for repeated measures, multiple devices/sites, and mixed periods?	Unclear	The archived report-level evidence does not establish this condition clearly.; Retrospective beryllium sensitization/chronic beryllium disease cardiopulmonary exercise testing cohort; two pulse-oximeter generations and two co-oximeter platforms are combined across eras; one maximum-exercise pair per participant in the beryllium sensitization estimate.	Methods and Results; pp. 1702 and 1705	B
CAMPEDELLI_2024	Campedelli et al., 2024	D1	D1Q1	Was enrollment consecutive/random or based on a clearly defined cohort without case-control enrichment?	Yes	The archived report-level evidence supports the low-risk condition.; Consecutive invasive-cardiopulmonary exercise testing cohort; ABL90 direct co-oximetry and exact blood-draw timing; peak analysis has one pair per participant, but signal failure can change sensor site and 28 patients lack complete peak data; reported CI is labelled as a limit of agreement.	Methods; Results; Fig. 2	B
CAMPEDELLI_2024	Campedelli et al., 2024	D1	D1Q2	Were exclusions and missing participants unlikely to depend on the index or reference result?	Yes	The archived report-level evidence supports the low-risk condition.; Consecutive invasive-cardiopulmonary exercise testing cohort; ABL90 direct co-oximetry and exact blood-draw timing; peak analysis has one pair per participant, but signal failure can change sensor site and 28 patients lack complete peak data; reported CI is labelled as a limit of agreement.	Methods; Results; Fig. 2	B
CAMPEDELLI_2024	Campedelli et al., 2024	D1	D1Q3	Was the analyzed comparison not restricted to a selectively chosen validation subgroup?	Yes	The archived report-level evidence supports the low-risk condition.; Consecutive invasive-cardiopulmonary exercise testing cohort; ABL90 direct co-oximetry and exact blood-draw timing; peak analysis has one pair per participant, but signal failure can change sensor site and 28 patients lack complete peak data; reported CI is labelled as a limit of agreement.	Methods; Results; Fig. 2	B/R
CAMPEDELLI_2024	Campedelli et al., 2024	D2	D2Q1	Were device, site, configuration/averaging, and changes sufficiently reported and stable?	Unclear	The archived report-level evidence does not establish this condition clearly.; Consecutive invasive-cardiopulmonary exercise testing cohort; ABL90 direct co-oximetry and exact blood-draw	Methods; Results; Fig. 2	B/R

Study ID	Author-year	Domain	Item	Signaling question	Final response	Rationale and study evidence	Evidence location	Item type
						timing; peak analysis has one pair per participant, but signal failure can change sensor site and 28 patients lack complete peak data; reported CI is labelled as a limit of agreement.		
CAMPEDELLI_2024	Campedelli et al., 2024	D2	D2Q2	Was the analyzed index the raw SpO2 rather than a post hoc corrected or secondary index?	Yes	The archived report-level evidence supports the low-risk condition.; Consecutive invasive-cardiopulmonary exercise testing cohort; ABL90 direct co-oximetry and exact blood-draw timing; peak analysis has one pair per participant, but signal failure can change sensor site and 28 patients lack complete peak data; reported CI is labelled as a limit of agreement.	Methods; Results; Fig. 2	B/R
CAMPEDELLI_2024	Campedelli et al., 2024	D2	D2Q3	Were signal-quality handling, failed readings, and index selection handled without evident result-dependent selection?	Yes	The archived report-level evidence supports the low-risk condition.; Consecutive invasive-cardiopulmonary exercise testing cohort; ABL90 direct co-oximetry and exact blood-draw timing; peak analysis has one pair per participant, but signal failure can change sensor site and 28 patients lack complete peak data; reported CI is labelled as a limit of agreement.	Methods; Results; Fig. 2	B
CAMPEDELLI_2024	Campedelli et al., 2024	D3	D3Q1	Was SaO2 obtained from a direct arterial sample using multiwavelength co-oximetry?	Yes	The archived report-level evidence supports the low-risk condition.; Consecutive invasive-cardiopulmonary exercise testing cohort; ABL90 direct co-oximetry and exact blood-draw timing; peak analysis has one pair per participant, but signal failure can change sensor site and 28 patients lack complete peak data; reported CI is labelled as a limit of agreement.	Methods; Results; Fig. 2	E/R
CAMPEDELLI_2024	Campedelli et al., 2024	D3	D3Q2	Was the functional/fractional saturation target clear and internally noncontradictory?	Unclear	Direct arterial co-oximetry is confirmed, but the functional/fractional saturation target is not explicitly defined; the review-defined D3Q2 condition is therefore unclear.; Consecutive invasive-cardiopulmonary exercise testing cohort; ABL90 direct co-oximetry and exact blood-draw timing; peak analysis has one pair per participant, but signal failure can change sensor site and 28 patients lack complete peak data; reported CI is labelled as a limit of agreement. The report identifies ABL90 Flex Plus co-oximetry and reports SaO2/oxyhaemoglobin alongside dyshemoglobins, but does not explicitly define a functional or fractional saturation calculation.	Methods, SO2 measurements (PDF p. 3; article p. 4)	B/R

Study ID	Author-year	Domain	Item	Signaling question	Final response	Rationale and study evidence	Evidence location	Item type
CAMPEDELLI_2024	Campedelli et al., 2024	D3	D3Q3	Was the reference result generated independently of the index result?	Yes	The archived report-level evidence supports the low-risk condition.; Consecutive invasive-cardiopulmonary exercise testing cohort; ABL90 direct co-oximetry and exact blood-draw timing; peak analysis has one pair per participant, but signal failure can change sensor site and 28 patients lack complete peak data; reported CI is labelled as a limit of agreement.	Methods; Results; Fig. 2	B
CAMPEDELLI_2024	Campedelli et al., 2024	D4	D4Q1	Were SpO2 and SaO2 paired contemporaneously during exercise or within two minutes after cessation?	Yes	The archived report-level evidence supports the low-risk condition.; Consecutive invasive-cardiopulmonary exercise testing cohort; ABL90 direct co-oximetry and exact blood-draw timing; peak analysis has one pair per participant, but signal failure can change sensor site and 28 patients lack complete peak data; reported CI is labelled as a limit of agreement.	Methods; Results; Fig. 2	B/R
CAMPEDELLI_2024	Campedelli et al., 2024	D4	D4Q2	Were eligible pairs included representatively and missing pairs or failed readings explained?	Unclear	The archived report-level evidence does not establish this condition clearly.; Consecutive invasive-cardiopulmonary exercise testing cohort; ABL90 direct co-oximetry and exact blood-draw timing; peak analysis has one pair per participant, but signal failure can change sensor site and 28 patients lack complete peak data; reported CI is labelled as a limit of agreement.	Methods; Results; Fig. 2	B
CAMPEDELLI_2024	Campedelli et al., 2024	D4	D4Q3	Did the analysis account for repeated measures, multiple devices/sites, and mixed periods?	Yes	The archived report-level evidence supports the low-risk condition.; Consecutive invasive-cardiopulmonary exercise testing cohort; ABL90 direct co-oximetry and exact blood-draw timing; peak analysis has one pair per participant, but signal failure can change sensor site and 28 patients lack complete peak data; reported CI is labelled as a limit of agreement.	Methods; Results; Fig. 2	B
ROBERTS_1999	Roberts et al., 1999	D1	D1Q1	Was enrollment consecutive/random or based on a clearly defined cohort without case-control enrichment?	No	The archived report-level evidence documents a failure or contradiction for this condition.; Only eight athlete volunteers formed the arterial-validation substudy; fixed forehead device and IL482 co-oximetry; 65 repeated pairs mix rest/exercise/recovery and saturation-threshold rows overlap.	Methods and Results; Fig. 2 and Table 5; p. 489	B
ROBERTS_1999	Roberts et al., 1999	D1	D1Q2	Were exclusions and missing participants unlikely to depend on the index or reference result?	Unclear	The archived report-level evidence does not establish this condition clearly.; Only eight athlete volunteers formed the arterial-validation substudy; fixed forehead device and IL482 co-oximetry; 65	Methods and Results; Fig. 2 and Table 5; p. 489	B

Study ID	Author-year	Domain	Item	Signaling question	Final response	Rationale and study evidence	Evidence location	Item type
						repeated pairs mix rest/exercise/recovery and saturation-threshold rows overlap.		
ROBERTS _1999	Roberts et al., 1999	D1	D1Q3	Was the analyzed comparison not restricted to a selectively chosen validation subgroup?	No	The archived report-level evidence documents a failure or contradiction for this condition.; Only eight athlete volunteers formed the arterial-validation substudy; fixed forehead device and IL482 co-oximetry; 65 repeated pairs mix rest/exercise/recovery and saturation-threshold rows overlap.	Methods and Results; Fig. 2 and Table 5; p. 489	B/R
ROBERTS _1999	Roberts et al., 1999	D2	D2Q1	Were device, site, configuration/averaging, and changes sufficiently reported and stable?	Yes	The archived report-level evidence supports the low-risk condition.; Only eight athlete volunteers formed the arterial-validation substudy; fixed forehead device and IL482 co-oximetry; 65 repeated pairs mix rest/exercise/recovery and saturation-threshold rows overlap.	Methods and Results; Fig. 2 and Table 5; p. 489	B/R
ROBERTS _1999	Roberts et al., 1999	D2	D2Q2	Was the analyzed index the raw SpO2 rather than a post hoc corrected or secondary index?	Yes	The archived report-level evidence supports the low-risk condition.; Only eight athlete volunteers formed the arterial-validation substudy; fixed forehead device and IL482 co-oximetry; 65 repeated pairs mix rest/exercise/recovery and saturation-threshold rows overlap.	Methods and Results; Fig. 2 and Table 5; p. 489	B/R
ROBERTS _1999	Roberts et al., 1999	D2	D2Q3	Were signal-quality handling, failed readings, and index selection handled without evident result-dependent selection?	Yes	The archived report-level evidence supports the low-risk condition.; Only eight athlete volunteers formed the arterial-validation substudy; fixed forehead device and IL482 co-oximetry; 65 repeated pairs mix rest/exercise/recovery and saturation-threshold rows overlap.	Methods and Results; Fig. 2 and Table 5; p. 489	B
ROBERTS _1999	Roberts et al., 1999	D3	D3Q1	Was SaO2 obtained from a direct arterial sample using multiwavelength co-oximetry?	Yes	The archived report-level evidence supports the low-risk condition.; Only eight athlete volunteers formed the arterial-validation substudy; fixed forehead device and IL482 co-oximetry; 65 repeated pairs mix rest/exercise/recovery and saturation-threshold rows overlap.	Methods and Results; Fig. 2 and Table 5; p. 489	E/R
ROBERTS _1999	Roberts et al., 1999	D3	D3Q2	Was the functional/fractional saturation target clear and internally noncontradictory?	Yes	The archived report-level evidence supports the low-risk condition.; Only eight athlete volunteers formed the arterial-validation substudy; fixed forehead device and IL482 co-oximetry; 65 repeated pairs mix rest/exercise/recovery and saturation-threshold rows overlap.	Methods and Results; Fig. 2 and Table 5; p. 489	B/R

Study ID	Author-year	Domain	Item	Signaling question	Final response	Rationale and study evidence	Evidence location	Item type
ROBERTS _1999	Roberts et al., 1999	D3	D3Q3	Was the reference result generated independently of the index result?	Yes	The archived report-level evidence supports the low-risk condition.; Only eight athlete volunteers formed the arterial-validation substudy; fixed forehead device and IL482 co-oximetry; 65 repeated pairs mix rest/exercise/recovery and saturation-threshold rows overlap.	Methods and Results; Fig. 2 and Table 5; p. 489	B
ROBERTS _1999	Roberts et al., 1999	D4	D4Q1	Were SpO2 and SaO2 paired contemporaneously during exercise or within two minutes after cessation?	Yes	The archived report-level evidence supports the low-risk condition.; Only eight athlete volunteers formed the arterial-validation substudy; fixed forehead device and IL482 co-oximetry; 65 repeated pairs mix rest/exercise/recovery and saturation-threshold rows overlap.	Methods and Results; Fig. 2 and Table 5; p. 489	B/R
ROBERTS _1999	Roberts et al., 1999	D4	D4Q2	Were eligible pairs included representatively and missing pairs or failed readings explained?	Unclear	The archived report-level evidence does not establish this condition clearly.; Only eight athlete volunteers formed the arterial-validation substudy; fixed forehead device and IL482 co-oximetry; 65 repeated pairs mix rest/exercise/recovery and saturation-threshold rows overlap.	Methods and Results; Fig. 2 and Table 5; p. 489	B
ROBERTS _1999	Roberts et al., 1999	D4	D4Q3	Did the analysis account for repeated measures, multiple devices/sites, and mixed periods?	No	The archived report-level evidence documents a failure or contradiction for this condition.; Only eight athlete volunteers formed the arterial-validation substudy; fixed forehead device and IL482 co-oximetry; 65 repeated pairs mix rest/exercise/recovery and saturation-threshold rows overlap.	Methods and Results; Fig. 2 and Table 5; p. 489	B
