# Supplementary material for: Pulse Oximetry Versus Arterial Co-Oximetry During Exercise: A Systematic Review of Agreement and Sensor Technology Reporting Gaps
Source: Biosensors (Basel). 2026 Oct 9;16(10):565. doi: 10.3390/bios16100565 (PMC13702209; doi:10.3390/bios16100565)
Supplement: Supplementary file 1 [file biosensors-16-00565-s001.zip › Supplementary_S3_Methodological_Appraisal.pdf]

Supplementary File S3: Descriptive methodological appraisal

Pulse Oximetry Versus Arterial Co-oximetry During Exercise: A Systematic Review of Agreement and Sensor-Technology Reporting Gaps

Both authors checked and agreed the final 192 item responses for the 16 included studies. Separate independent rating forms were not retained. The historical 12-item framework drew on QUADAS-2 domains, but QUADAS-2 concerns diagnostic accuracy rather than continuous agreement. We therefore use the retained evidence as a descriptive methodological appraisal, distinguishing potential bias mechanisms, reporting or construct ambiguity, and eligibility checks. We do not calculate an overall risk-of-bias grade or use an any-No algorithm in the current synthesis. Missing reporting indicates uncertainty rather than proof of bias. Supplementary Files S2 and S3 provide the unchanged author-agreed responses, source locations and an interpretive codebook; original aggregate categories are retained only in the provenance archive. This descriptive framework is not an externally validated continuous-agreement appraisal tool.

Interpretation rules

Selection and missingness: describe the actual sampling frame, analysed denominator and exclusions; missing information is uncertainty, not proof of bias.

Index test: distinguish raw output from post hoc correction and describe changed devices/sites; do not conflate transportability with internal bias.

Reference: direct co-oximetry eligibility is distinct from clarity of functional or fractional saturation. Do not treat eligibility as an overall risk grade.

Timing and dependence: distinguish contemporaneous peak pairs from repeated stages, multiple correlated devices and mixed periods. Unavailable covariance limits synthesis.

Reporting: optical or processing omissions describe incomplete disclosure, not device failure. GRRAS is a reporting checklist, not a validated risk-of-bias tool.

Provenance: the agreed 192 item responses and evidence locations are retained unchanged. Interpretation labels are editorial classification, not newly fabricated independent author ratings.

Study-specific evidence

Study	Source limitation	Location
ASCHA_2018	Complete-pair restriction could affect representativeness, but the retained outcome is one	Methods; Table 2 Methods, Pulse Oximetry and Arterial Oxygenation Determinations (article p. 1993); Table 2

	contemporaneous peak pair per analysed participant. The source confirms direct co-oximetry but leaves the functional/fractional target incompletely specified.	
SWIGRIS_2009	Device/site heterogeneity precludes a stable index-test estimate even though the reference is direct. The source confirms direct co-oximetry but leaves the functional/fractional target incompletely specified.	Methods; Results; Fig. 1 Methods, Maximal cardiopulmonary exercise test (article p. 255); Fig. 1
ZEBALLOS_1991	No study outcome is restricted to the protocol's exercise-only analytical unit.	Methods; pp. 1241-1243
YAMAYA_2002	The all-data primary estimates cannot be treated as independent participant-level observations.	Methods; p. 165
HANSEN_1987	Non-extractability limits precision synthesis but does not itself establish biased measurement.	Methods; p. 335
BENOIT_1997	Retained only as technology-history/sensitivity evidence; not valid for the raw-SpO2 primary comparison.	Methods; p. 261
MCGOVERN_1996	The raw device result is eligible, but repeated pairs cannot be treated as 49 independent observations.	Methods and Results; p. 1153
CARONE_1997	The 47 sixth-minute clinical-cutoff pairs are not the Bland-Altman agreement denominator.	Methods and Results; p. 448
MARTIN_1992	Direct fractional HbO2 is not silently interchangeable with functional SaO2 for the review's primary target.	Methods and Results; p. 456
BARTHELEMY_1990	No prespecified single index device and no within-subject covariance are available.	Methods and Results; p. 535
POWERS_1989	A condition-specific estimate is structurally usable only after choosing one configuration; fractional HbO2 creates a reference-definition applicability	Methods; Table 1; p. 301

	concern.	
RIES_1985	Exercise-only direction is available, but the variance and independence conditions for pooling are not.	Methods; Tables 1-3; pp. 686-688
RICE_1999	External technical verification supported eligibility; the article's graphical result remains qualitative.	Methods; Fig. 4
LUNDGREN_2001	The beryllium sensitization estimate has a paired SD, but an unstratified mixed index technology limits a common target effect. The source confirms direct co-oximetry but leaves the functional/fractional target incompletely specified.	Methods and Results; pp. 1702 and 1705 Methods, Exercise Physiology Evaluation (article p. 1704)
CAMPEDELLI_2024	The point estimate is usable descriptively; the reported spread is not treated as a trusted paired-difference variance. The source confirms direct co-oximetry but leaves the functional/fractional target incompletely specified.	Methods; Results; Fig. 2 Methods, SO2 measurements (PDF p. 3; article p. 4)
ROBERTS_1999	The minimum-SaO2 row is conditionally defined and must not be combined independently with the aggregate or threshold rows.	Methods and Results; Fig. 2 and Table 5; p. 489
