# Supplementary material for: Pulse Oximetry Versus Arterial Co-Oximetry During Exercise: A Systematic Review of Agreement and Sensor Technology Reporting Gaps
Source: Biosensors (Basel). 2026 Oct 9;16(10):565. doi: 10.3390/bios16100565 (PMC13702209; doi:10.3390/bios16100565)
Supplement: Supplementary file 1 [file biosensors-16-00565-s001.zip › Supplementary_S4_eTables_S7–S10.pdf]

Supplementary File S4

Pulse Oximetry Versus Arterial Co-oximetry During Exercise: A Systematic Review of Agreement and Sensor-Technology Reporting Gaps

Shu Xie; Xueming Wen

e-Table S7. Outcome compatibility gate

Defined in the operational protocol before quantitative synthesis. Review eligibility and pooling compatibility are separate: all included rows met the direct arterial co-oximetry reference criterion, whereas the pooling column evaluates construct exchangeability within a common target.

Study	Representative outcome	Eligible timing	Direct co-oximetry reference eligible for review	Independent/ resolved unit	Paired variance usable	Reference construct compatible for pooling	Clinical/technical stratum	Current synthesis route; not a risk grade	Study-level route	Primary blocking reason
ASCHA_ 2018	ASCHA_2018_P EAK	Yes	Yes	Yes	Yes	Conditional	Forehead; contemporary clinical cardiopulmonary exercise testing referral cohort	Explorato ry mean-bias only	Descriptive study-level candidate	No common three-study device-site-population target; the source-reported arterial saturation construct is not specified as functional or fractional.

**Study**	**Representative outcome**	**Eligible timing**	**Direct co-oximetry reference eligible for review**	**Independent/ resolved unit**	**Paired variance usable**	**Reference construct compatible for pooling**	**Clinical/technical stratum**	Current synthesis route; not a risk grade	**Study-level route**	**Primary blocking reason**
SWIGRIS_2009	SWIGRIS_2009_MAX_EXERCISE	Yes	Yes	Yes	Yes	Conditional	Finger primary with earlobe backup; systemic sclerosis-associated interstitial lung disease; Denver altitude; changing devices	Exploratory mean-bias only	Descriptive study-level candidate	Changing index/reference platforms, site backup, altitude-specific context, and an incompletely specified arterial saturation construct prevent a stable common target.
ZEBALLOS_1991	ZEBALLOS_1991_HP_SEA_LEVEL	No	Yes	No	Conditional	Yes	Historic earlobe devices; young male recruits; normoxia/hypoxia	Descriptive synthesis only	SWiM narrative	Published estimates combine rest, exercise, and recovery with repeated observations.

Study	Representative outcome	Eligible timing	Direct co-oximetry reference eligible for review	Independent/ resolved unit	Paired variance usable	Reference construct compatible for pooling	Clinical/technical stratum	Current synthesis route; not a risk grade	Study-level route	Primary blocking reason
YAMAYA_2002	YAMAYA_2002_RS10_FOREHEAD	No	Yes	No	No	Yes	Multiple sites/devices; healthy/athlete/clinical participants; normoxia/hypoxia	Descriptive synthesis only	SWiM narrative	All-data estimates combine rest/exercise and oxygen conditions; within-person covariance is not recoverable.
HANSEN_1987	HANSEN_1987_BIOX_TIMECOURSE	Conditional	Yes	No	No	Yes	Historic earlobe device; clinical exercise-test patients	Descriptive synthesis only	SWiM narrative	Exercise time-course comparison is graphical only and not a numeric paired agreement estimate.

Study	Representative outcome	Eligible timing	Direct co-oximetry reference eligible for review	Independent/ resolved unit	Paired variance usable	Reference construct compatible for pooling	Clinical/technical stratum	Current synthesis route; not a risk grade	Study-level route	Primary blocking reason
BENOIT_ 1997	BENOIT_1997_C ORRECTED_LO W_SAO2	Condition al	Yes	No	No	Yes	Earlobe; healthy men; normoxia/hypoxia	Descripti ve synthesis only	Secondary technology-history evidence	Reported index result is post-hoc corrected SpO2 rather than raw device output.
MCGOVE RN_1996	MCGOVERN_19 96_RAW_SPO2	Yes	Yes	No	No	Yes	Index finger; severe COPD; progressive cycling	Descripti ve synthesis only	SWiM narrative	49 repeated pairs in eight participants have no recoverable clustering for a study weight.

Study	Representative outcome	Eligible timing	Direct co-oximetry reference eligible for review	Independent/ resolved unit	Paired variance usable	Reference construct compatible for pooling	Clinical/technical stratum	Current synthesis route; not a risk grade	Study-level route	Primary blocking reason
CARONE_1997	CARONE_1997_BIOX_ALL_TIMEPOINTS	No	Yes	No	No	Yes	Earlobe; COPD on long-term oxygen therapy; walking with oxygen titration	Descriptive synthesis only	SWiM narrative	Agreement set mixes baseline/repeated walking/titration and has no agreement-set pair count.
MARTIN_1992	MARTIN_1992_FINGER_EXERCISE	Yes	Yes	No	No	No	Multiple ear/finger sites; elite male cyclists	Descriptive synthesis only	SWiM narrative	Repeated correlated observations are unresolved. The direct fractional co-oximetry endpoint is eligible for this review but is not interchangeable with functional or incompletely specified saturation strata for pooling.

Study	Representative outcome	Eligible timing	Direct co-oximetry reference eligible for review	Independent/ resolved unit	Paired variance usable	Reference construct compatible for pooling	Clinical/technical stratum	Current synthesis route; not a risk grade	Study-level route	Primary blocking reason
BARTHELEMY_1990	BARTHELEMY_1990_NELLCOR_75_T00	Yes	Yes	No	No	Yes	Eight historic finger devices; moderate cycle exercise	Descriptive synthesis only	SWiM narrative	Device rows comprise repeated observations and correlated multi-device measurements with no covariance.
POWERS_1989	POWERS_1989_OHMEDA3700_EAR_HIGHEST	Yes	Yes	Conditional	Conditional	No	Ear/finger configurations; healthy men; normoxia/hypoxia	Descriptive synthesis only	Conditional sensitivity evidence	Three simultaneous configurations are correlated. The direct fractional co-oximetry endpoint is eligible for this review but is not interchangeable with functional or incompletely specified saturation strata for pooling.

Study	Representative outcome	Eligible timing	Direct co-oximetry reference eligible for review	Independent/ resolved unit	Paired variance usable	Reference construct compatible for pooling	Clinical/technical stratum	Current synthesis route; not a risk grade	Study-level route	Primary blocking reason
RIES_1985	RIES_1985_HP_ALL_PHASES_EXERCISE	Yes	Yes	No	No	Yes	Historic earlobe devices; pulmonary-laboratory patients	Descriptive synthesis only	SWiM narrative	Repeated phase-overlapping observations and regression SEE do not yield a paired-difference variance.
RICE_1999	RICE_1999_EAR_GRAPHICAL_EXERCISE	No	Yes	No	No	Conditional	Earlobe; competitive male cyclists; incremental cycling	Descriptive synthesis only	SWiM narrative	Comparison is graphical across rest and repeated exercise; no numeric effect or denominator is extracted.

Study	Representative outcome	Eligible timing	Direct co-oximetry reference eligible for review	Independent/ resolved unit	Paired variance usable	Reference construct compatible for pooling	Clinical/technical stratum	Current synthesis route; not a risk grade	Study-level route	Primary blocking reason
LUNDGREN_2001	LUNDGREN_2001_BES_MAX	Yes	Yes	Yes	Yes	Conditional	Earlobe; beryllium sensitization clinical cardiopulmonary exercise testing; two historical index generations	Exploratory mean-bias only	Descriptive study-level candidate	Both index and reference platforms changed over the accrual era; the beryllium sensitization population, earlobe target, and incompletely specified arterial saturation construct are not exchangeable with at least two other candidates.
CAMPEDELLI_2024	CAMPEDELLI_2024_PEAK_PRIMARY	Yes	Yes	Yes	Yes	Conditional	Finger primarily with earlobe/forehead fallback; invasive clinical cardiopulmonary exercise testing	Exploratory mean-bias only	Descriptive study-level candidate	Paired variance is recoverable, but sensor-site fallback, the distinct invasive-cardiopulmonary exercise testing population, and an incompletely specified functional/fractional endpoint leave no three-study exchangeable target.
ROBERTS_1999	ROBERTS_1999_MINIMUM_SAO2	Conditional	Yes	Conditional	Conditional	Yes	Forehead; athletes; supramaximal cycling	Descriptive synthesis only	Conditional sensitivity evidence	Minimum-SaO2 selection and overlapping threshold rows make the result non-representative and non-independent.

e-Table S8. Peak/maximal compatibility addendum

Three peak/maximal cohorts provide reported paired SDs; a fourth is conditional on reconstruction from an ordinary paired-mean CI. The post hoc sensitivity analysis is presented in Supplementary File S8, not as individual agreement or a common clinical correction.

Study	Population	Index device and site	Exercise time point	Arterial co-oximetry endpoint	Paired SD status	Decisive reason not combined
Ascha 2018	Mixed clinical cardiopulmonary exercise testing referrals; 631 complete peak pairs	Nonin Avant 4000; forehead	Peak sample during final 1-2 minutes before stopping	ABL800 source-reported arterial SaO2; functional/fractional construct not specified	Usable paired SD 2.75 percentage points	No common set of at least three exchangeable device-site-population targets; forehead contemporary referral cohort differs materially from the other candidates
Swigris 2009	Systemic-sclerosis-associated interstitial lung disease; 83 participants	Changing pulse-oximeter platforms; mostly finger with earlobe backup	Maximum exercise	Source-reported arterial SaO2 from co-oximeter plus blood-gas analyzer; functional/fractional construct not specified	Usable paired SD 4.00 percentage points	Changing index/reference platforms, site backup, altitude-specific clinical context, and source-specific methodological limitations prevent a stable common target
Lundgren 2001	Beryllium-sensitized subgroup; 67 participants	Two historical pulse-oximeter generations; earlobe	Maximum exercise	Source-reported arterial SaO2 from two co-oximeter generations; functional/fractional construct not specified	Usable paired SD 4.60 percentage points	Both index and reference platforms changed over the accrual era; the beryllium sensitization population and earlobe target are not exchangeable with at least two other candidates
Campedelli 2024	Invasive-cardiopulmonary exercise testing referrals; 268 complete peak pairs	Stryker system; finger with earlobe/forehead fallback	Peak exercise	ABL90 Flex Plus source-reported SaO2/oxyhaemoglobin; functional/fractional calculation not explicitly defined	Paired SD approximately 7.19 percentage points, reconstructed from the reported 95% CI of the paired mean and n=268	Despite a recoverable paired SD, sensor-site fallback, a distinct invasive-cardiopulmonary exercise testing population, and an incompletely specified functional/fractional endpoint prevent membership in a three-study exchangeable technical-clinical stratum

e-Table S9. SWiM direction-and-magnitude evidence profile

Four structured descriptive synthesis contexts; exploratory mean-bias pooling is separately identified in Supplementary File S8.

Synthesis group	Studies	Representative outcomes	Signed bias (SpO2 minus arterial endpoint)	Precision/agreement reporting	Why not pooled	Interpretation without formal certainty grades	Rationale
Peak/maximal clinical cardiopulmonary exercise testing; one reported pair per participant	ASCHA_2018 | SWIGRIS_2009 | LUNDGREN_2001 | CAMPEDELLI_2024	ASCHA_2018_PEAK | SWIGRIS_2009_MAX_EXERCISE | LUNDGREN_2001_BES_MAX | CAMPEDELLI_2024_PEAK_PRIMARY	Ascha +2.63; Swigris +1.50; Lundgren beryllium sensitization -0.88; Campedelli -0.44	Ascha SD 2.75 and limits of agreement -2.9 to +8.1; Swigris SD 4.00; Lundgren SD 4.60; Campedelli reconstructed SD approximately 7.19 and sign-harmonized 95% CI -1.31 to +0.42	No pooled primary target; post hoc exploratory mean-bias sensitivity is reported separately in	No formal certainty grade;	Different clinical and technology contexts, incompletely specified saturation constructs and only

Synthesis group	Studies	Representative outcomes	Signed bias (SpO2 minus arterial endpoint)	Precision/agreement reporting	Why not pooled	Interpretation without formal certainty grades	Rationale
					Supplementary File S8. The conditional fourth SD is not directly reported.	see source limitations and Supplementar	one report with individual LoA prevent a general interchangeability conclusion.

<table>
<tr><th>Synthesis group</th><th>Studies</th><th>Representative outcomes</th><th>Signed bias (SpO2 minus arterial endpoint)</th><th>Precision/agreement reporting</th><th>Why not pooled</th><th>Interpretation without formal certainty grades</th><th>Rationale</th></tr>
<tr><td></td><td></td><td></td><td></td><td></td><td></td><td>y File S8.</td><td></td></tr>
<tr><td>Exercise-only historical device comparisons with repeated observations</td><td>MCGOVERN_1996 | MARTIN_1992 | BARTHELEMY_1990 | POWERS_1989 | RIES_1985</td><td>MCGOVERN_1996_RAW_SPO2 | MARTIN_1992_FINGER_EXERCISE | BARTHELEMY_1990_NELLCOR_75_100 | POWERS_1989_OHMEDA3700_EAR_HIGHEST | RIES_1985_HP_ALL_PHASES_EXERCISE</td><td>McGovern -1.70; Martin device/site range -0.59 to +0.87; Barthélémy device range -3.26 to +0.89; Powers highest-work range -2.77 to -0.87; Ries +0.30 to +0.90</td><td>Reported SDs for selected rows; Ries reports regression SEE rather than paired-difference SD</td><td>Repeated within-person pairs, simultaneous devices, and overlapping phases create unresolved dependence; the direct fractional endpoints in Martin and Powers are eligible for the review but are not interchangeable with functional or incompletely specified saturation strata for pooling.</td><td>No formal certainty grade; see</td><td>Unavailable within-person covariance, multiple correlated configurations and nonidentical saturation constructs</td></tr>
</table>

**Synthesis group**	**Studies**	**Representative outcomes**	**Signed bias (SpO2 minus arterial endpoint)**	**Precision/agreement reporting**	**Why not pooled**	Interpretation without formal certainty grades	**Rationale**
						source limitations and Supplementary File S8.	limit the effective independent information.

Synthesis group	Studies	Representative outcomes	Signed bias (SpO2 minus arterial endpoint)	Precision/agreement reporting	Why not pooled	Interpretation without formal certainty grades	Rationale
Mixed rest/exercise/recovery or condition-combined comparisons	ZEBALLOS_1991 | YAMAYA_2002 | CARONE_1997 | ROBERTS_1999	ZEBALLOS_1991_HP_SEA_LEVEL | YAMAYA_2002_RS10_FOREHEAD | CARONE_1997_BIOX_ALL_TIMEPOINTS | ROBERTS_1999_A	Zeballos HP/Biox and altitude estimates range -4.8 to +9.8; Yamaya device range -2.0 to +0.3; Carone -0.67; Roberts aggregate +0.10	SDs/limits available for selected summaries, but they reflect mixed timepoints or repeated observations	Primary review timing is not separable; conditions/sites/devices vary; pair denominators and dependence are incomplete.	No formal certainty grade; see source limitat	Mixed timepoints and conditions do not isolate the target exercise window; exercise-only denominator and dependence may be unrecoverable.

Synthesis group	Studies	Representative outcomes	Signed bias (SpO2 minus arterial endpoint)	Precision/agreement reporting	Why not pooled	Interpretation without formal certainty grades	Rationale
		LL_REST_EXERCISE				ions and Supplementary File S8.	
Qualitative or secondary-index evidence	HANSEN_1987 | BENOIT_1997 | RICE_1999	HANSEN_1987_BIOX_TIMECOURSE | BENOIT_1997_CORRECTED_LOW_SAO2 | RICE_1999_EAR_GRAPHICAL_EXERCISE	Hansen graphical only; Benoit corrected SpO2 +1.80 to +2.05; Rice reports SpO2 higher than temperature-adjusted SaO2 across exercise intensities	No extractable raw paired estimate for Hansen/Rice; Benoit is a post-hoc corrected index	Graphical-only reporting, post-hoc correction, or mixed repeated data makes quantitative synthesis inappropriate.	No formal	Graphical, corrected-index or qualitative

Synthesis group	Studies	Representative outcomes	Signed bias (SpO2 minus arterial endpoint)	Precision/agreement reporting	Why not pooled	Interpretation without formal certainty grades	Rationale
						certainty grade; see source limitations and	results do not provide comparable raw paired estimates with usable uncertainty.

Synthesis group	Studies	Representative outcomes	Signed bias (SpO2 minus arterial endpoint)	Precision/agreement reporting	Why not pooled	Interpretation without formal certainty grades	Rationale
						Supplementary File S8.	

e-Table S10. Narrative interpretation limitations, without formal certainty grades

The historical GRADE-informed matrix is archived for provenance. Current interpretation does not mechanically inherit aggregate categories from an unvalidated appraisal framework.

Context	Study / reporting limitations	Indirectness / inconsistency	Imprecision and interpretive boundary
Peak/maximal independent pairs	Device/site changes and incomplete arterial-construct definition	Clinical populations and technology contexts differ	Only 3 reported-SD cohorts; conditional fourth; average bias cannot represent individual LoA
Repeated exercise observations	Unavailable within-person covariance; multiple devices or stages	Correlated device/site estimates and mixed saturation targets	Do not count outcome rows as independent studies or use raw pair counts as independent participant n
Mixed rest/exercise or indirect agreement	Exercise-only estimate or denominator not recoverable	Mixed periods cannot isolate exercise agreement	Narrative evidence; no exercise-specific pooled precision
Qualitative / graphical support	Paired bias or variance not extractable	Selected populations and older devices limit transportability	Supports context only; not an estimate of individual interchangeability
