# Supplementary material for: Pulse Oximetry Versus Arterial Co-Oximetry During Exercise: A Systematic Review of Agreement and Sensor Technology Reporting Gaps
Source: Biosensors (Basel). 2026 Oct 9;16(10):565. doi: 10.3390/bios16100565 (PMC13702209; doi:10.3390/bios16100565)
Supplement: Supplementary file 1 [file biosensors-16-00565-s001.zip › Supplementary_S5_Citation_Summary.pdf]

Supplementary File S5

Pulse Oximetry Versus Arterial Co-oximetry During Exercise: A Systematic Review of Agreement and Sensor-Technology Reporting Gaps

Shu Xie; Xueming Wen

e-Table S11. Citation-search disposition summary

The complete 1506-row ledger is provided in Supplementary File S6. No new record was promoted to retrieval after two-reviewer consensus.

Citation-search category	Records, No.	Reader-facing disposition
Unique citation-graph records	1506	All deduplicated backward and forward citation-search records
Already represented in database corpus	164	No new full-text action required
Database overlap: exact DOI	145	Matched an existing database-corpus record by DOI
Database overlap: exact title	19	Matched an existing database-corpus record by title
New records independently screened	1342	Two-reviewer title/abstract screening with consensus disposition
Promoted to full-text retrieval	0	No additional report was promoted
Excluded: no eligible pulse-oximetry index	710	Title/abstract did not establish an eligible pulse-oximetry index
Excluded: no eligible exercise time point	461	No dynamic-exercise or immediate-recovery comparison
Excluded: ineligible report type	137	Not an eligible full research report
Excluded: no direct arterial co-oximetry reference	34	No paired direct arterial co-oximetry reference was established
