# Supplementary material for: Pulse Oximetry Versus Arterial Co-Oximetry During Exercise: A Systematic Review of Agreement and Sensor Technology Reporting Gaps
Source: Biosensors (Basel). 2026 Oct 9;16(10):565. doi: 10.3390/bios16100565 (PMC13702209; doi:10.3390/bios16100565)
Supplement: Supplementary file 1 [file biosensors-16-00565-s001.zip › Supplementary_S6_Source_Data/author_agreed_original/Received_17study_appraisal_original/04_Additional_3_Risk_of_Bias.pdf]

Additional file 3. Risk-of-bias judgments

Agreement of Pulse Oximetry With Arterial Co-oximetry During and Immediately After Exercise in Adults: A Systematic Review

Xueming Wen; Shu Xie

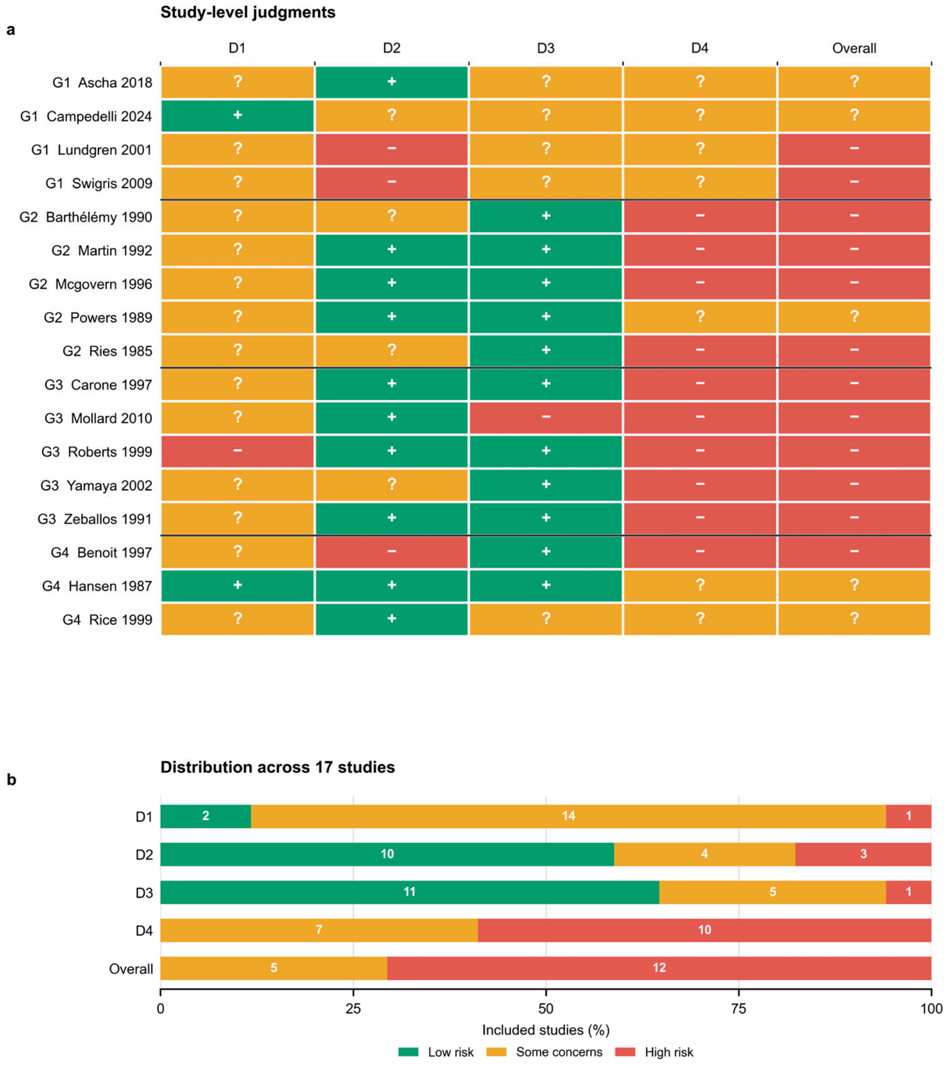

D1, participant selection and spectrum; D2, index-test conduct; D3, reference standard; D4, flow, timing, and unit of analysis. Symbols: + low risk; ? some concerns; − high risk. Applicability is assessed separately.

e-Figure 1 | Adapted QUADAS-2-informed risk-of-bias judgments. a, Study-level judgments across participant selection and spectrum (D1), index-test conduct (D2), reference standard (D3), and flow, timing, and unit of analysis (D4), followed by the overall judgment. Studies are ordered within the four non-pooled synthesis groups. **b,** Distribution of judgments across the 17 included studies; percentages are accompanied by exact counts. The 12 signaling questions and decision algorithm were review-defined rather than standard QUADAS-2 output. Applicability is assessed separately and is not shown. Both reviewers performed the risk-of-bias assessment and agreed the final judgments; the framework and 204 signaling-question responses are provided in Additional file 1 (e-Appendix 3) and Additional file 2 (e-Table 6).
