# Supplementary material for: Pulse Oximetry Versus Arterial Co-Oximetry During Exercise: A Systematic Review of Agreement and Sensor Technology Reporting Gaps
Source: Biosensors (Basel). 2026 Oct 9;16(10):565. doi: 10.3390/bios16100565 (PMC13702209; doi:10.3390/bios16100565)
Supplement: Supplementary file 1 [file biosensors-16-00565-s001.zip › Supplementary_S7_Technology_Audit.pdf]

Supplementary File S7. Technology-reporting audit

Purpose. This editor-requested post hoc audit evaluates what the 16 finally included exercise reports disclosed about the optical biosensor and its signal-processing context. It is a reporting-completeness audit, not a performance score or causal feature analysis.

Coding rule. Reported = device-specific operational detail; Partial = the domain was mentioned without sufficient device-specific detail; NR = not reported. Numerical emitter wavelengths were required for Reported. No unreported property was inferred from a model name, external manual, patent, or current manufacturer material.

e-Table S12. Domain-level reporting summary. Counts of Reported, Partial, and Not Reported (NR) classifications across 10 technology-reporting domains in the 16 included studies, with operational coding criteria.

Domain	Reported	Partial	NR	Operational criterion
Device/model	14	2	0	Manufacturer/model or device generation
Optical geometry	2	0	14	Transmission, reflectance, or explicit emitter-detector arrangement
Sensor site	15	1	0	Anatomical measurement site
Wavelengths	0	4	12	Numerical emitter wavelengths required for Reported
Averaging/update	2	3	11	Display update interval, time constant, or averaging window
Motion handling	6	4	6	Fixation-independent motion rule, filter, or artifact assessment
Perfusion/signal quality	11	3	2	Perfusion intervention, indicator, threshold, or quality criterion
Fixation/light control	8	0	8	Probe fixation, cable restraint, or ambient-light shielding
Algorithm/processing	3	5	8	Calibration, algorithm, filter, correction, or post-processing
Failure/missingness	7	3	6	Failed readings, rejected waves, or missing-data denominator

e-Table S13. Study-level status matrix. Reporting status for each of the 10 domains in each included study, comprising 160 study-domain judgments. R = Reported; P = Partial; NR = Not Reported.

Study	Ref	Year	Device/model	Optical geometry	Sensor site	Wavelengths	Averaging/update	Motion handling	Perfusion/signal quality	Fixation/light control	Algorithm/processing	Failure/missingness
RIES_1985	14	1985	R	NR	R	NR	P	P	R	R	P	NR
HANSEN_1987	26	1987	R	NR	R	NR	NR	NR	R	R	NR	P
POWERS_1989	21	1989	R	NR	R	NR	NR	R	NR	R	NR	NR
BARTHELEMY_1990	20	1990	R	NR	R	P	NR	R	P	NR	P	R
ZEBALLOS_1991	15	1991	R	NR	R	P	NR	NR	P	R	P	NR
MARTIN_1992	16	1992	R	NR	R	NR	NR	P	P	NR	NR	R
MCGOVERN_1996	17	1996	R	NR	R	P	R	P	R	NR	R	R
CARONE_1997	23	1997	R	NR	R	NR	NR	R	R	R	NR	NR
BENOIT_1997	28	1997	R	R	R	NR	NR	NR	R	NR	R	R
RICE_1999	27	1999	R	NR	R	P	R	P	R	NR	R	NR
ROBERTS_1999	25	1999	R	R	R	NR	P	R	NR	R	P	R
LUNDGREN_2001	18	2001	R	NR	R	NR	NR	NR	R	NR	NR	NR
YAMAYA_2002	22	2002	R	NR	R	NR	P	R	R	R	P	R

Study	Ref	Year	Device/model	Optical geometry	Sensor site	Wavelengths	Averaging/update	Motion handling	Perfusion/signal quality	Fixation/light control	Algorithm/processing	Failure/missingness
SWIGRIS_2009	1	2009	P	NR	R	NR	NR	NR	R	NR	NR	P
ASCHA_2018	3	2018	R	NR	R	NR	NR	R	R	R	NR	P
CAMPEDELLI_2024	19	2024	P	NR	P	NR	NR	NR	R	NR	NR	R

Abbreviations: R, Reported; P, Partial; NR, not reported. The matrix contains 160 study-domain judgments.

e-Table S14. Source-located technology extraction. Extracted device, sensor-site, and technology-reporting details for the included studies, with source-text locators and audit notes. Unreported properties were not inferred from device model names or external materials.

Study (reference; year)	Device and site	Technology and signal detail	Evidence locator and audit note
RIES_1985 ([14]; 1985)	Device/model [Reported]: Hewlett-Packard 47201A and Biox IIA ear oximeters<br>Site [Reported]: Earlobe	Optical geometry [NR]: Optical geometry not stated<br>Wavelengths [NR]: No pulse-oximeter wavelength values or counts stated<br>Averaging/update [Partial]: Biox used in fast-response mode; no time constant stated<br>Motion handling [Partial]: Tape/headband and manufacturer retainer were described, without an explicit motion test<br>Perfusion/signal quality [Reported]: Ear rubbing plus Hewlett-Packard 47205A perfusion indicator<br>Fixation/light control [Reported]: Cover, gauze, tape/headband, and ear-probe retainer<br>Algorithm/processing [Partial]: Fast-response mode and calibration checks stated; internal algorithm not described<br>Failure/missingness [NR]: Signal-failure denominator not stated	RIES_1985.txt lines 112-136<br>Audit note: Do not infer optical geometry or wavelengths from the model name
HANSEN_1987 ([26]; 1987)	Device/model [Reported]: Biox IIA<br>Site [Reported]: Earlobe	Optical geometry [NR]: Optical ear probe stated; transmission/reflectance not stated<br>Wavelengths [NR]: No pulse-oximeter wavelength information<br>Averaging/update [NR]: Continuous output stated; averaging or update interval not stated<br>Motion handling [NR]: No prospective motion-artifact rule stated<br>Perfusion/signal quality [Reported]: Ear rubbing and a perfusion monitor were used<br>Fixation/light control [Reported]: Ear retainer or tape<br>Algorithm/processing [NR]: No pulse-oximeter signal-processing algorithm described<br>Failure/missingness [Partial]: Low-perfusion alarm behavior discussed, but no signal-failure count	HANSEN_1987.txt lines 15-20 and 55-60<br>Audit note: Four-wavelength wording refers to the arterial co-oximeter, not the pulse oximeter
POWERS_1989 ([21]; 1989)	Device/model [Reported]: Ohmeda 3700 finger and ear configurations; Biox IIA ear configuration<br>Site [Reported]: Index finger and both ears	Optical geometry [NR]: Transmission/reflectance not stated<br>Wavelengths [NR]: No pulse-oximeter wavelength information<br>Averaging/update [NR]: No pulse-oximeter averaging or update interval<br>Motion handling [Reported]: Ear cables were fixed by headband and the finger was splinted to reduce movement<br>Perfusion/signal quality [NR]: No perfusion index or signal-quality threshold stated<br>Fixation/light control [Reported]: Headband cable fixation and splinted index finger<br>Algorithm/processing [NR]: No pulse-oximeter algorithm or calibration detail<br>Failure/missingness [NR]: No signal-failure denominator stated	POWERS_1989.txt lines 53-81<br>Audit note: Reference co-oximeter was four-wavelength; this was not assigned to the pulse oximeters
BARTHELEMY_1990 ([20]; 1990)	Device/model [Reported]: Eight named finger pulse oximeters<br>Site [Reported]: Finger	Optical geometry [NR]: Transmission/reflectance not reported for individual devices<br>Wavelengths [Partial]: General two-wavelength pulse-oximetry principle stated; device-specific wavelengths	BARTHELEMY_1990.txt lines 108-164 and 316-384<br>Audit note: General sensing principle was not treated as device-specific wavelength disclosure

Study (reference; year)	Device and site	Technology and signal detail	Evidence locator and audit note
		absent<br>Averaging/update [NR]: No pulse-oximeter averaging or update interval<br>Motion handling [Reported]: Exercise was used to reproduce movement artifacts; invalid pulse waves were tracked<br>Perfusion/signal quality [Partial]: Low regional flow was discussed, but no perfusion-handling protocol was reported<br>Fixation/light control [NR]: Probe fixation and ambient-light control not reported<br>Algorithm/processing [Partial]: Nellcor ECG synchronization and device rejection of invalid pulse waves were reported without algorithm details<br>Failure/missingness [Reported]: Failure to display SpO2 and rejected pulse waves were recorded	
ZEBALLOS_1991 ([15]; 1991)	Device/model [Reported]: Hewlett-Packard 47201A and Biox IIA<br>Site [Reported]: Both earlobes	Optical geometry [NR]: Transmission/reflectance not stated<br>Wavelengths [Partial]: HP was described as using eight wavelengths; numerical wavelengths and Biox values were absent<br>Averaging/update [NR]: No pulse-oximeter averaging or update interval<br>Motion handling [NR]: Secure supports were used, but motion handling was not explicitly evaluated<br>Perfusion/signal quality [Partial]: Ears were rubbed with alcohol; no perfusion metric was reported<br>Fixation/light control [Reported]: HP support/headband, Biox retainer, and darkened laboratory<br>Algorithm/processing [Partial]: HP internal calibration for pigmentation was described at a high level<br>Failure/missingness [NR]: No signal-failure denominator stated	ZEBALLOS_1991.txt lines 126-143 and 257-300<br>Audit note: Eight-wavelength detail was reported only for HP and did not include wavelengths in nm
MARTIN_1992 ([16]; 1992)	Device/model [Reported]: Three Ohmeda 3740 units<br>Site [Reported]: Both earlobes and one finger	Optical geometry [NR]: Transmission/reflectance not stated<br>Wavelengths [NR]: No pulse-oximeter wavelength information<br>Averaging/update [NR]: No pulse-oximeter averaging or update interval<br>Motion handling [Partial]: Loss of signal during exercise was reported, but no motion-control protocol was stated<br>Perfusion/signal quality [Partial]: Signal loss was quantified; no perfusion intervention or index was reported<br>Fixation/light control [NR]: Probe fixation not reported<br>Algorithm/processing [NR]: No pulse-oximeter algorithm or processing rule stated<br>Failure/missingness [Reported]: Twenty-eight samples excluded; about 90% of missing readings arose from ear-probe signal loss	MARTIN_1992.txt lines 18-35 and 91-99<br>Audit note: Signal loss was not automatically attributed to motion or perfusion
MCGOVERN_1996 ([17]; 1996)	Device/model [Reported]: Ohmeda 3700<br>Site [Reported]: Index finger opposite arterial line	Optical geometry [NR]: Transmission/reflectance not stated<br>Wavelengths [Partial]: Two-wavelength operation stated; numerical wavelengths absent<br>Averaging/update [Reported]: Rolling 6-12 s display average<br>Motion handling [Partial]: Waveform and signal checks were used; direct motion-suppression method not reported<br>Perfusion/signal quality [Reported]: Signal intensity, dicrotic notch, and pulse-rate/ECG concordance assessed<br>Fixation/light control [NR]: Probe placement stated; fixation design not reported<br>Algorithm/processing [Reported]: Self-calibration and an explicit post hoc COHb-correction equation were reported<br>Failure/missingness [Reported]: Adequate signal and dicrotic notch obtained for all included exercise points	MCGOVERN_1996.txt lines 74-104 and 176-238<br>Audit note: Rolling average was a display characteristic reported by the source
CARONE_1997 ([23]; 1997)	Device/model [Reported]: Biox 3760<br>Site [Reported]: Earlobe	Optical geometry [NR]: Transmission/reflectance not explicitly stated<br>Wavelengths [NR]: No pulse-oximeter wavelength information<br>Averaging/update [NR]: No pulse-oximeter averaging or update interval<br>Motion handling [Reported]: Stabilizer, cable routing,	CARONE_1997.txt lines 88-117<br>Audit note: The phrase transmission cable was not treated as proof of transmission-mode optics

Study (reference; year)	Device and site	Technology and signal detail	Evidence locator and audit note
		headband, and dark cloth addressed movement and ambient light<br>Perfusion/signal quality [Reported]: Earlobe massaged with alcohol for 30 s to increase perfusion<br>Fixation/light control [Reported]: Ear stabilizer, routed cable, elastic headband, and dark cloth<br>Algorithm/processing [NR]: No pulse-oximeter algorithm or processing rule stated<br>Failure/missingness [NR]: Signal-failure denominator not reported	
BENOIT_1997 ([28]; 1997)	Device/model [Reported]: Datex Satlite<br>Site [Reported]: Earlobe	Optical geometry [Reported]: Pulsed-to-fixed transmitted-light ratio explicitly reported<br>Wavelengths [NR]: No pulse-oximeter wavelength information<br>Averaging/update [NR]: No pulse-oximeter averaging or update interval<br>Motion handling [NR]: No explicit motion-artifact protocol<br>Perfusion/signal quality [Reported]: Vasodilator ointment and a pulsed-to-fixed signal-ratio threshold were used<br>Fixation/light control [NR]: Ear probe stated; fixation not described<br>Algorithm/processing [Reported]: Published post hoc correction equation was applied before comparison<br>Failure/missingness [Reported]: Five hypoxic samples excluded for low absorption signal; <10% ratio threshold	BENOIT_1997.txt lines 80-125<br>Audit note: Corrected SpO2 was retained as a secondary-index result, not raw-device performance
RICE_1999 ([27]; 1999)	Device/model [Reported]: Criticare US-504<br>Site [Reported]: Earlobe	Optical geometry [NR]: Transmission/reflectance not stated<br>Wavelengths [Partial]: Two-wavelength sensor stated; numerical wavelengths absent<br>Averaging/update [Reported]: Monitor output was summarized as 30 s averages<br>Motion handling [Partial]: R-wave coordination was used; no direct motion-suppression test was reported<br>Perfusion/signal quality [Reported]: Earlobe pretreated with a rubefacient and signal coordinated with ECG<br>Fixation/light control [NR]: Sensor applied to earlobe; fixation not described<br>Algorithm/processing [Reported]: External 30 s averaging was specified; proprietary device algorithm not described<br>Failure/missingness [NR]: Signal-failure denominator not reported	RICE_1999.txt lines 152-160<br>Audit note: The 30 s average was produced from monitor output in the study computer
ROBERTS_1999 ([25]; 1999)	Device/model [Reported]: Nellcor N200 with RS-10 sensor<br>Site [Reported]: Forehead	Optical geometry [Reported]: Reflectance forehead sensor<br>Wavelengths [NR]: No numerical pulse-oximeter wavelength information<br>Averaging/update [Partial]: Displayed value recorded every 5 s; internal averaging not stated<br>Motion handling [Reported]: Headband and controlled cable handling were used to reduce motion artifact<br>Perfusion/signal quality [NR]: Perfusion loss was discussed, but no prospective perfusion threshold was used<br>Fixation/light control [Reported]: Headband and technician-held interface cable<br>Algorithm/processing [Partial]: SatMaster software recorded real-time output; proprietary processing not described<br>Failure/missingness [Reported]: Four exercise points invalidated because of signal loss	ROBERTS_1999.txt lines 126-157 and 332-344<br>Audit note: Five-second recording interval was not relabelled as an averaging interval
LUNDGREN_2001 ([18]; 2001)	Device/model [Reported]: Ohmeda 3740 (1985-1993) and SensorMedics Sat-Trak (1993-1998)<br>Site [Reported]: Earlobe	Optical geometry [NR]: Transmission/reflectance not stated<br>Wavelengths [NR]: No pulse-oximeter wavelength information<br>Averaging/update [NR]: No pulse-oximeter averaging or update interval<br>Motion handling [NR]: No explicit motion-artifact protocol<br>Perfusion/signal quality [Reported]: Earlobe rubbed to improve perfusion<br>Fixation/light control [NR]: Probe fixation not reported<br>Algorithm/processing [NR]: Device changes were reported; internal algorithms were not	LUNDGREN_2001.txt lines 140-168<br>Audit note: Two device eras and two reference-platform eras were mixed in one cohort

Study (reference; year)	Device and site	Technology and signal detail	Evidence locator and audit note
		Failure/missingness [NR]: Signal-failure denominator not reported	
YAMAYA_2002 ([22]; 2002)	Device/model [Reported]: Nellcor N-395/RS-10, Nellcor N-395/D-25, and Ivy 2000/Masimo SET LNOP-Adt<br>Site [Reported]: Forehead and digits	Optical geometry [NR]: Forehead and digit sensor designs stated; transmission/reflectance not explicitly stated<br>Wavelengths [NR]: No numerical pulse-oximeter wavelength information<br>Averaging/update [Partial]: Three devices recorded over a synchronized 30 s acquisition window; internal averaging not stated<br>Motion handling [Reported]: Opaque shielding, forehead sweatband, and pulse-rate/ECG signal-quality analysis<br>Perfusion/signal quality [Reported]: Weak-signal and altered-digital-perfusion performance addressed; heart-rate discordance flagged<br>Fixation/light control [Reported]: Opaque shield lightly taped over digit sensors; forehead sensor secured with sweatband<br>Algorithm/processing [Partial]: Signal-detection algorithms were compared at a high level; proprietary details absent<br>Failure/missingness [Reported]: Poor pulse-rate detection identified in device-specific observations and analyzed with/without exclusion	YAMAYA_2002.txt lines 85-124 and 224-281<br>Audit note: Thirty-second acquisition window was not treated as a stated internal averaging constant
SWIGRIS_2009 ([1]; 2009)	Device/model [Partial]: Peripheral oximeters changed across three decades; models not identified<br>Site [Reported]: Index finger primarily; earlobe backup	Optical geometry [NR]: Transmission/reflectance not stated<br>Wavelengths [NR]: No pulse-oximeter wavelength information<br>Averaging/update [NR]: No pulse-oximeter averaging or update interval<br>Motion handling [NR]: No explicit motion-artifact rule<br>Perfusion/signal quality [Reported]: Adequate pulse signal required; earlobe substituted when finger signal was inadequate<br>Fixation/light control [NR]: Site-substitution rule reported; fixation not described<br>Algorithm/processing [NR]: Changing devices acknowledged; algorithms not identified<br>Failure/missingness [Partial]: >95% used finger probes; fallback frequency only approximately reported	SWIGRIS_2009.txt lines 83-87 and 109-113<br>Audit note: Model-level attribution was impossible because device identities were absent
ASCHA_2018 ([3]; 2018)	Device/model [Reported]: Nonin Avant 4000<br>Site [Reported]: Forehead	Optical geometry [NR]: Forehead probe stated; reflectance/transmission not explicitly stated<br>Wavelengths [NR]: No pulse-oximeter wavelength information<br>Averaging/update [NR]: No pulse-oximeter averaging or update interval<br>Motion handling [Reported]: Two probes, waveform inspection, dicrotic notch, heart-rate synchronization, and forehead site<br>Perfusion/signal quality [Reported]: Waveform and pulse concordance were required; forehead hypoperfusion discussed as a residual risk<br>Fixation/light control [Reported]: Two forehead probes secured with an elastic band<br>Algorithm/processing [NR]: No pulse-oximeter algorithm or calibration detail<br>Failure/missingness [Partial]: Most reliable of two signals selected; signal-rejection denominator not reported	ASCHA_2018.txt lines 77-81 and 125-131<br>Audit note: No optical geometry was inferred from the forehead site
CAMPEDELLI_2024 ([19]; 2024)	Device/model [Partial]: Stryker Sustainability Solutions pulse oximeter; model not reported<br>Site [Partial]: Index finger planned; earlobe or forehead backup; final site not recorded	Optical geometry [NR]: Transmission/reflectance not stated<br>Wavelengths [NR]: No pulse-oximeter wavelength information<br>Averaging/update [NR]: No pulse-oximeter averaging or update interval<br>Motion handling [NR]: Motion artifact discussed, but no prospective suppression rule was reported<br>Perfusion/signal quality [Reported]: Probe moved when SpO2 was inconsistent; missingness associated with Raynaud/scleroderma and race<br>Fixation/light control [NR]: Probe design and fixation not reported<br>Algorithm/processing [NR]: No device algorithm or	CAMPEDELLI_2024.txt lines 216-236 and 580-597<br>Audit note: Manufacturer name without model was coded Partial; actual site per measurement was unavailable

Study (reference; year)	Device and site	Technology and signal detail	Evidence locator and audit note
		processing rule stated<br>Failure/missingness [Reported]: SpO2 missing in 18% of all stages and 8.4% of peak stages	

Machine-readable companion: Supplementary_S7_Technology_Audit_Source.csv (inside Supplementary File S6). Figure 2 is generated from the same audited judgments.
