# Supplementary material for: Pulse Oximetry Versus Arterial Co-Oximetry During Exercise: A Systematic Review of Agreement and Sensor Technology Reporting Gaps
Source: Biosensors (Basel). 2026 Oct 9;16(10):565. doi: 10.3390/bios16100565 (PMC13702209; doi:10.3390/bios16100565)
Supplement: Supplementary file 1 [file biosensors-16-00565-s001.zip › Supplementary_S8_Revision_Analyses.pdf]

Supplementary File S8: Revision analyses and search accounting

Pulse Oximetry Versus Arterial Co-oximetry During Exercise: A Systematic Review of Agreement and Sensor-Technology Reporting Gaps

A. Post hoc peak/maximal mean-bias sensitivity

The primary synthesis describes paired mean differences in percentage points, paired SDs and individual limits of agreement where reported. The original review-specific compatibility rule required three independent studies within a common clinical and measurement stratum; this is not a universal statistical requirement. SWiM-guided direction-and-magnitude synthesis avoids significance vote counting. We added a post hoc exploratory random-effects mean-bias analysis of three independent peak/maximal cohorts with reported paired SDs, and a four-cohort sensitivity analysis adding the conditional 2024 SD. One estimate per cohort was used; correlated devices, stages and overlapping subgroups were not treated as independent studies. Sampling variance was SD squared divided by the participant count, with REML between-study variance and modified Hartung–Knapp confidence intervals using k−1 degrees of freedom. Disease, device/site and arterial-construct differences limit the interpretation of the average. Pooling mean bias does not estimate individual agreement or a transferable correction factor. With only three or four heterogeneous cohorts, meta-regression and small-study tests were not performed. Supplementary File S8 reports inputs, assumptions and reproducible calculations.

Cohort	Participant n	SpO2−arterial bias (pp)	Paired SD (pp)	Status
LUNDGREN_2001_BES_MAX	67	-0.88	4.6	Reported paired SD
ASCHA_2018_PEAK	631	2.63	2.75	Reported paired SD
SWIGRIS_2009_MAX_EXERCISE	83	1.5	4.0	Reported paired SD
CAMPEDELLI_2024_PEAK_PRIMARY	268	-0.44	7.19	Conditional CI reconstruction

Denominators follow the locked extraction: one selected peak/maximal comparison per participant. In particular the Swigris analysis uses the reported cohort denominator of 83; an independently reported effective peak-pair denominator was not recovered. Results depend on that assumption. Overlapping Campedelli subgroup rows are not added.

The exploratory reported-SD analysis of three peak/maximal cohorts gave a mean bias of 1.14 percentage points (modified Hartung–Knapp 95% CI -3.29 to 5.57; Q-based I^2 95.3%). Adding the conditional Campedelli SD gave 0.75 (95% CI -1.90 to 3.41; I^2 96.4%). These small-k

estimates are post hoc, clinically heterogeneous and dependent on reported cohort denominators; they do not resolve saturation-construct ambiguity or identify individual limits of agreement. The four-study result additionally assumes an ordinary paired-mean confidence interval for the 2024 reconstruction. No common clinical correction is recommended (Supplementary File S8).

For an ordinary two-sided paired-mean t interval, SDdifference=(upper−lower)×sqrt(n)/(2×t0.975,n−1). With n=268 and bounds −0.42 to 1.31 in the source sign convention, the reconstructed SD is approximately 7.19 pp. This does not require a separately known correlation when the interval is already for paired differences. It is not valid if the interval estimates another statistic, is adjusted, bootstrap-based or otherwise dependence-adjusted. The source interval method is unconfirmed; this value is excluded from the reported-SD analysis and must not be interpreted as reported individual LoA.

The REML optimizer was cross-checked with SciPy bounded minimization. The modified Hartung–Knapp standard error is the maximum of the usual random-effects standard error and the residual-scaled Hartung–Knapp standard error. The confidence interval uses a t quantile with k−1 degrees of freedom. Q-based I^2 is descriptive and highly uncertain with small k. Meta-regression would be underpowered and confounded with only three or four cohorts.

B. Embase accounting and author-confirmed screening

Searches #225 and #207 are different queries, not interchangeable exports. Their union is an audit working set, not a final search yield. Platform counts include a separate clinical-trial registry tab. Do not report all 1,427 or 1,518 results as article records.

Record set	Count	Meaning
225 articles displayed	1196	1190 recoverable unique identifiers; six unavailable records due to author-confirmed export failure, not screening exclusions
225 trial records displayed	231	231 exported IDs; separate from article reports
207 articles displayed	1255	1247 complete article identifiers recovered; export is incomplete
207 trial records displayed	263	262 exported IDs
Cross-query article union	1254	Not the count of either search
Author-confirmed initial screening queue	725	Title/abstract screening, not 725 full texts
Audited new queue records	711	Subtract 9 old-corpus links and 5 internal duplicate records
Other distinct author-excluded reports	7	Outside the initial queue
Audited title/abstract exclusion subset	718	711+7; not a complete four-database flow

The final supplementary Embase search (#225, 29 September 2026) displayed 1196 article records. The supplied article export contained 1190 recoverable unique identifiers. The authors confirmed that the six-record shortfall resulted from an export failure. These six records were not available for screening and were not counted as duplicates, title/abstract exclusions or full-text exclusions. The authors confirmed completion of screening of the supplied records. Search #207 is retained only as audit history and is not added to the final search denominator. The 231 clinical-trial registry records are reported separately from article records. Figure 1 retains the original database/citation-search flow. The supplementary search is reported here with its explicit export-stage loss; a fully screened merged four-database flow is not claimed. The 718 distinct title/abstract exclusions remain an audited subset, not the entire exported article set. No missing records or full-text decisions are invented.

C. Version and exclusion provenance

The author-confirmed Mollard exclusion reduces the original eligible set from 17 studies/49 outcomes to 16 studies/46 outcomes, and the agreed appraisal from 204 to 192 items. Martin 2015 and Hansen 2026 remain excluded according to the supplied author decisions; they are not silently added to the formal evidence set. The two-author agreed 17-study archive is preserved verbatim with SHA-256 in Supplementary File S6.

Final author decisions, 30 September 2026: search #225 (1196 articles) is the final supplementary search. Search #207 is historical only. Chailleux 1989 was obtained and excluded under E04 according to both authors, giving eight not retrieved, 34 assessed and 18 excluded in the original search branch. Game 2003 was already included in the three later-linked duplicate records and is not deducted again. Both authors approved the substantive revision. On 1 October 2026 the authors confirmed that the six-identifier shortfall resulted from export failure. Six records were unavailable for screening; screening of supplied records was complete. This explanation is author-reported, not an independently reproduced platform diagnostic.
